# Synthesis of 5-(Aryl)amino-1,2,3-triazole-containing 2,1,3-Benzothiadiazoles via Azide–Nitrile Cycloaddition Followed by Buchwald–Hartwig Reaction

**DOI:** 10.3390/molecules29092151

**Published:** 2024-05-06

**Authors:** Pavel S. Gribanov, Anna N. Philippova, Maxim A. Topchiy, Dmitry A. Lypenko, Artem V. Dmitriev, Sergey D. Tokarev, Alexander F. Smol’yakov, Alexey N. Rodionov, Andrey F. Asachenko, Sergey N. Osipov

**Affiliations:** 1 A.N. Nesmeyanov Institute of Organoelement Compounds, Russian Academy of Sciences, 28/1 Vavilova Str., 119334 Moscow, Russia; anyfil96@gmail.com (A.N.P.); tokarev@ineos.ac.ru (S.D.T.); rengenhik@gmail.com (A.F.S.); rodalex@ineos.ac.ru (A.N.R.); 2 A.V. Topchiev Institute of Petrochemical Synthesis, Russian Academy of Sciences, Leninskiy Prospect 29, 119991 Moscow, Russia; maxtopchiy@yandex.ru (M.A.T.); aasachenko@gmail.com (A.F.A.); 3 A.N. Frumkin Institute of Physical Chemistry and Electrochemistry, Russian Academy of Sciences, Leninsky Prospect 31, Bld. 4, 119071 Moscow, Russia; dalypenko@gmail.com (D.A.L.); oleduff@mail.ru (A.V.D.)

**Keywords:** benzothiadiazole, triazole, cross-coupling, amination, luminophore, OLED

## Abstract

An efficient access to the novel 5-(aryl)amino-1,2,3-triazole-containing 2,1,3-benzothiadiazole derivatives has been developed. The method is based on 1,3-dipolar azide–nitrile cycloaddition followed by Buchwald–Hartwig cross-coupling to afford the corresponding *N*-aryl and *N*,*N*-diaryl substituted 5-amino-1,2,3-triazolyl 2,1,3-benzothiadiazoles under NHC-Pd catalysis. The one-pot diarylative Pd-catalyzed heterocyclization opens the straightforward route to triazole-linked carbazole-benzothiadiazole D-A systems. The optical and electrochemical properties of the compound obtained were investigated to estimate their potential application as emissive layers in OLED devises. The quantum yield of photoluminescence (PLQY) of the synthesized D-A derivatives depends to a large extent on electron-donating strengths of donor (D) component, reaching in some cases the values closed to 100%. Based on the most photoactive derivative and wide bandgap host material mCP, a light-emitting layer of OLED was made. The device showed a maximum brightness of 8000 cd/m^2^ at an applied voltage of 18 V. The maximum current efficiency of the device reaches a value of 3.29 cd/A.

## 1. Introduction

Polyheteroaromatic compounds containing donor–acceptor (D-A) units linked through π-conjugation have been drawing considerable attention due to their unique electrochemical and photochemical properties. They have found many important applications in organic electronics and luminescence materials [1,2,3]. In the past decade, various electron-deficient (hetero)aromatics have been widely used as acceptor blocks in developing advanced functional materials. Among them, 2,1,3-benzothiadiazole (BTD) derivatives are recognized as privileged building blocks for assembling various optoelectronic devices [4], e.g., organic light-emitting diodes (OLEDs) [5,6], field effect transistors (OFETs) [7,8,9], solar cells (OSCs) [10,11,12,13,14], luminescent solar concentrators (LSCs) [15], as well as fluorescent tags for molecular and cellular imaging [16,17,18,19]. The successes of these benzothiadiazole-based D-A molecules are mainly beneficial from their ability to provide for intramolecular charge-transfer effects. The nature of donor and acceptor components and the linker between them are particularly crucial factors that affect the properties of such compounds [20]. The introduction of rigid chromophores with twisted molecular geometry or additional substituents into the linker can prevent unfavorable π-aggregates in the solid state, which often causes fluorescence quenching. Therefore, the development of new benzothiadiazole-based molecules with extended π-conjugation is currently of great interest.

The aromatic triazole ring is a versatile and readily installed linkage that has been used to extend the conjugation between different aromatic systems. Usually, it can be easily achieved via copper-catalyzed azide-alkyne cycloaddition, well known as “click” reaction. The beneficial effect of 1,2,3-triazole-linked BTD derivatives has been demonstrated for some dyes with a high photo and chemical stability in their excited states, which is a very desirable feature for both the technological and biological applications of new fluorophores [18,21,22,23,24,25].

**Scheme 1:**
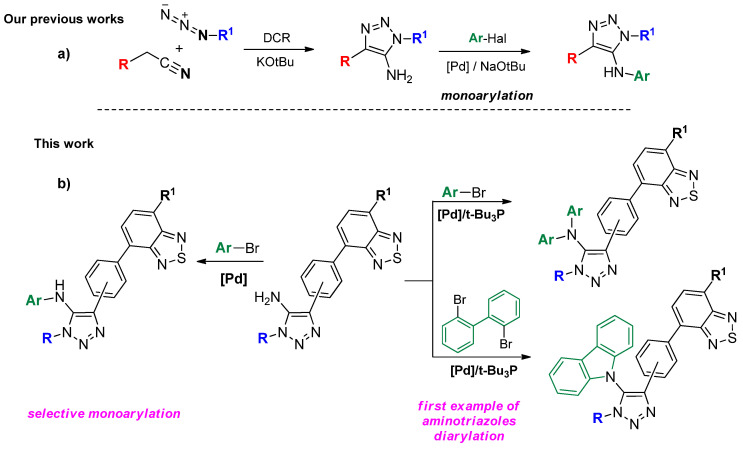
Synthetic approaches to: (**a**) 5-amino-1,2,3-triazoles and 5-arylamino-1,2,3-triazoles; (**b**) BTD-containing *N*-aryl- and *N*,*N*-diarylsubstituted 5-amino-1,2,3-triazoles.

An alternative route to 1,2,3-triazole core includes dipolar [3 + 2]-cycloaddition reaction (DCR) between azides and monosubstituted acetonitriles furnishing the corresponding 5-amino substituted products [26,27,28,29,30], which are impossible to obtain using classical azide–alkyne ligation. The presence of the amino group in these compounds provides a unique opportunity to tune their properties by introducing various functional substituents. Based on DCR methodology, we have recently developed an efficient pathway to polyarylated 5-amino-1,2,3-triazoles [31,32] (Scheme 1a). Now, we wish to report on the first application of this approach in combination with subsequent Buchwald–Hartwig reaction to the synthesis of novel 5-(aryl)amino-1,2,3-triazole-linked 2,1,3-benzothiadiazole derivatives (Scheme 1b).

## 2. Results

### 2.1. Synthesis

**Scheme 2:**
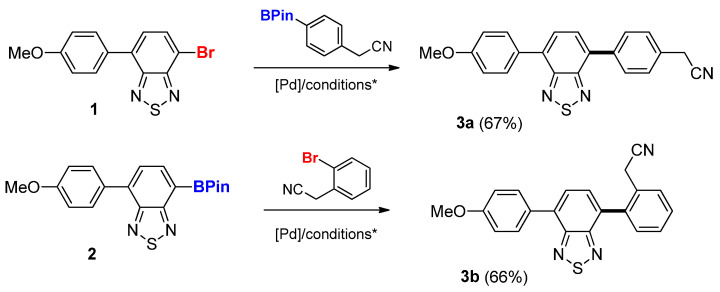
Synthesis of acetonitrile containing BTDs **3a** and **3b**. * Reaction conditions: **1** or **2** (1 equiv.), nitrile (1.2 equiv.), NaHCO_3_ (3 equiv.), Pd(PPh_3_)_2_Cl_2_ (5 mol.%), dioxane/H_2_O, 100 °C, 6 h.

Starting nitrile **3a** was synthesized by the Pd-catalyzed cross-coupling reaction of readily available *mono*-Br-substituted BTD **1** [33,34] with boropinacolate derivative of 2-phenylacetonitrile under standard Suzuki conditions. The reversed approach has proved to be more effective for the preparation of nitrile **3b**. In this case, coupling between specially prepared 4-BPin-substituted BTD **2** (see Section 3.2) and *ortho*-Br 2-phenylacetonitrile led to the formation of the desired BTD-containing nitrile **3b** in an acceptable yield using the same catalytic system (Scheme 2).

Then, following our previous experience [31] and literature precedents [29], we performed the pre-optimization of the dipolar [3 + 2]-cycloaddition (DCR) for the reaction of BTD-nitrile **3a** with benzyl azide by screening simple catalysts (K_2_CO_3_, Cs_2_CO_3_, KOtBu) and solvents (DMF, DMSO) under moderate heating (40–80 °C). As a result, we found that the best yield of the desired product **4a** (94%) can be achieved using 3.0 equiv. of azide, 50 mol.% of KOtBu in DMSO solution at 70 °C for 3 h. With a lower catalyst loading of catalyst (20–30 mol.%) or azide (1–2 equiv.), the reaction became less efficient with respect to rate and yield.

**Scheme 3:**
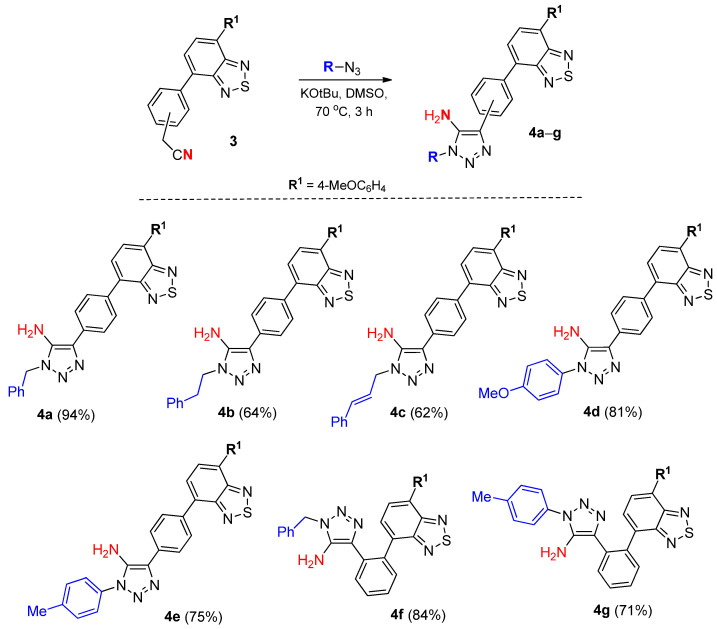
Synthesis of BTD-containing 5-amino-1,2,3-triazoles **4a**–**g**. Reaction conditions: **3a** or **3b** (3 mmol), aryl (alkyl) azide (3 equiv.), KOtBu (0.5 equiv.), DMSO (8 mL), 70 °C, 3 h.

These conditions were further applied for the cycloaddition of nitriles **3a** and **3b** with different aliphatic and aromatic azides to afford the corresponding 5-amino-1,2,3-triazole-linked BTDs **4a**–**g** in good to excellent yields (Scheme 3).

**Scheme 4:**
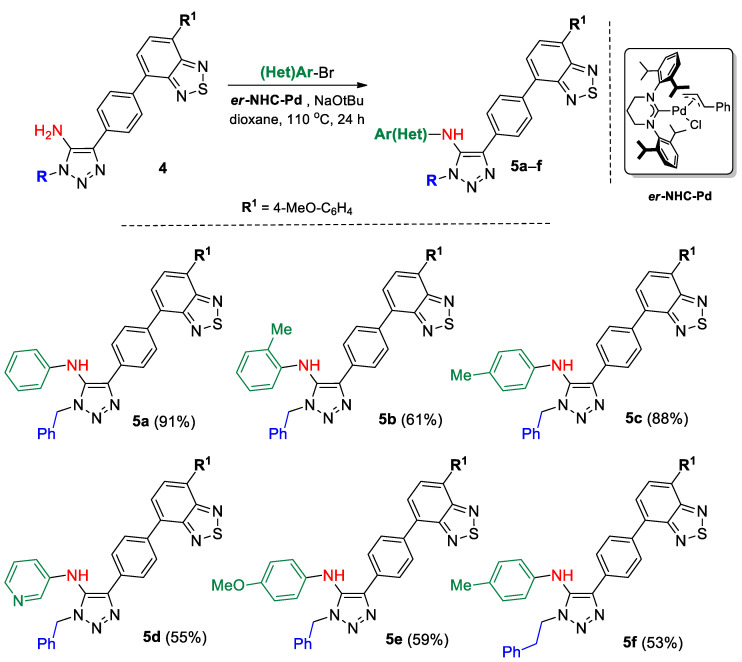
Buchwald–Hartwig cross-coupling synthesis of *N*-monosubstituted arylamino-1,2,3-triazole-2,1,3-benzothiadiazoles **5a**–**f**. Reaction conditions: **4** (0.2 mmol), (het)aryl-Br (5 equiv.), (THP-Dipp)Pd(cinn)Cl (5 mol.%), NaOtBu (3 equiv.), 1,4-dioxane (1.0 mL), 110 °C, 24 h.

The Buchwald–Hartwig amination (BHA) is one of the most efficient cross-coupling reactions to access a wide range of *N*-mono- and *N*,*N*-disubstituted aryl amines. Despite noticeable advances in this area [35,36,37,38,39], coupling heteroaromatic amines with arylhalides is still a challenging transformation that often requires time-consuming searches for optimal conditions and catalytic systems [40,41,42,43,44,45,46,47]. In our previous study, we revealed that palladium complex with expanded-ring NHC ligand (THP-Dipp)Pd(cinn)Cl (*er*-NHC-Pd) is the most competent catalyst in the BHA reaction for low-reactive 5-amino-1,2,3-triazoles [32]. Now, we investigated its activity in the BHA of amino-triazoles **4** with different (het)aryl bromides. First, we checked the reaction of **4a** with phenyl bromide using the same conditions, namely, equal amounts of the reagents were heated in 1,4-dioxane at 110 °C (oil bath temperature) in the presence of 2 mol.% of *er*-NHC-Pd catalyst and 3.0 equiv. of sodium *tert*-butoxide for 24 h. As a result, the desired cross-coupling product **5a** was isolated in 58% yield. The increase in the catalyst loading up to 5 mol.% and the amount of phenyl bromide up to 5 equiv. allowed reaching the full conversion of the starting amine **4a** and, as a consequence, improving the yield of **5a** to 91% (Scheme 4). With these conditions in hand, a series of *N*-aryl substituted 5-amino-1,2,3-triazole-linked BTDs **5a**–**f** was synthesized in good to excellent yields (Scheme 4). It should be noted that *N*,*N*-diarylated products were not detected in the reaction mixtures despite using a large excess of amine component. This phenomenon can be rationalized by the unique feature of Pd-catalyst with bulky NHC ligand [48,49].

**Scheme 5:**
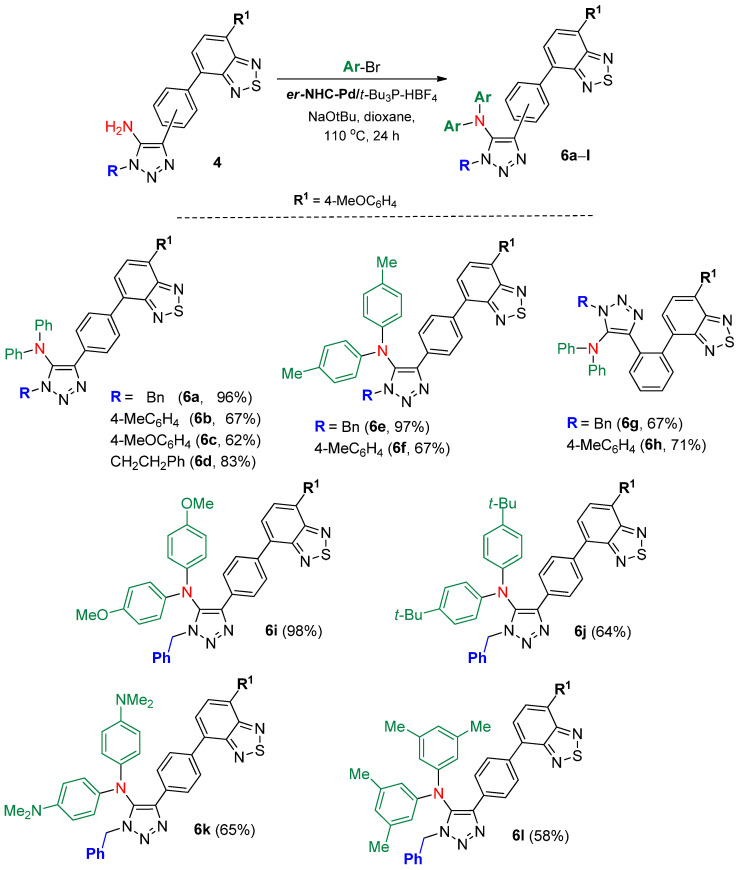
The Buchwald–Hartwig cross-coupling synthesis of *N*,*N*-disubstituted-arylamino-1,2,3-triazole-2,1,3-benzothiadiazoles **6a**–**l**. Reaction conditions: 5-amino-1,2,3-triazole-2,1,3-benzothiadiazole **4** (0.2 mmol), aryl-Br (5 equiv.), (THP-Dipp)Pd(cinn)Cl (5 mol.%), *t*-Bu_3_P-HBF_4_ (10 mol.%), NaOtBu (3 equiv.), 1,4-dioxane (1.0 mL), 110 °C, 24 h.

It was previously shown that the introduction of auxiliary ligands to NHC-Pd complexes can provide new beneficial features in their catalytic activity [50,51,52,53]. We recently established [54] that a combination of *er*-NHC-Pd with phosphine ligand can efficiently catalyze the arylation both of primary and secondary amines to produce the corresponding triaryl amines. This finding has prompted us to investigate the double Buchwald–Hartwig coupling of NH_2_-containing BTD compounds **4** with aryl bromides using a combination of the *er*-NHC-Pd complex with commercially available *tert*-butyl phosphine (protected as the BF_4_ salt). Thus, we initially tested a catalytic activity of *er*-NHC-Pd/*t*-Bu_3_P·HBF_4_ (5/10 mol.%) in the model reaction of **4a** with phenyl bromide. Fortunately, we revealed that the reaction smoothly proceeds in 1,4-dioxane at 110 °C in the presence of 3.0 equiv. of sodium *tert*-butoxide and goes to completion for 24 h, furnishing the desired *N*,*N*-diphenyl derivative **6a** in excellent yield and selectivity. These conditions were further applied for the preparation of a series of the corresponding *N*,*N*-diarylated 5-amino-1,2,3-triazol-linked BTDs **6a**–**l** in high yields (Scheme 5).

It is worth noting that this reaction demonstrates the first example of a double Buchwald–Hartwig cross-coupling reaction of 5-amino-1,2,3-triazoles with aryl halogenides. 

Given the great value of electron–donor carbazole block in the development of advanced optoelectronic materials [55,56,57,58], as well as to be inspired by the results described above, we decided to study a possibility to perform one-pot diarylative cyclization of primary aminogroup of **4** with 2,2′-dibromobiaryls under found catalytic conditions. 

**Scheme 6:**
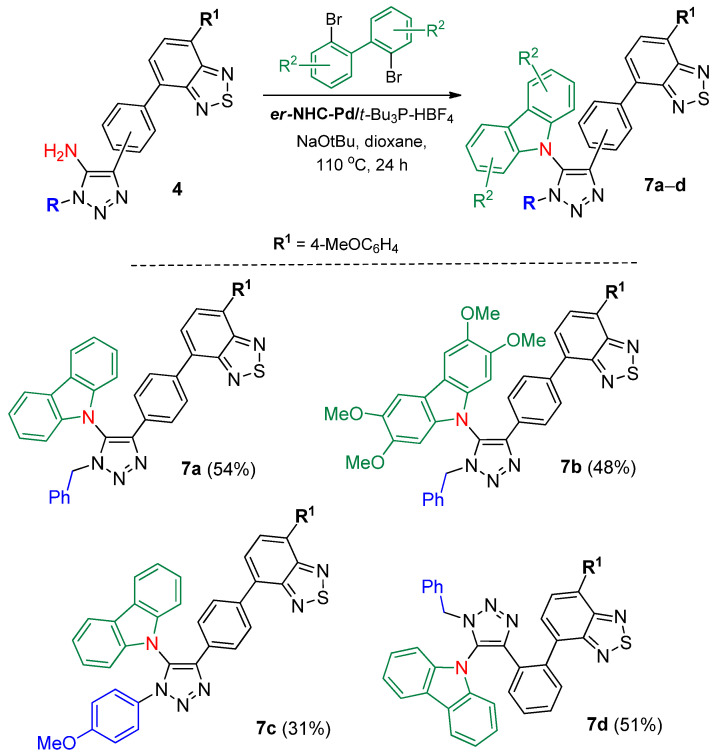
One-pot diarylative cyclization synthesis of **7a**–**d**. Reaction conditions: 5-amino-1,2,3-triazole-2,1,3-benzothiadiazole **4** (0.2 mmol), 2,2′-dibromobiphenyl (1.0 equiv.), (THP-Dipp)Pd(cinn)Cl (5 mol.%), *t*-Bu_3_P-HBF_4_ (10 mol.%), NaOtBu (3 equiv.), 1,4-dioxane (1.0 mL), 110 °C, 24 h.

As it was expected, the same catalytic system *er*-NHC-Pd/*t*-Bu_3_P·HBF_4_ (5/10 mol.%) has proved to be sufficiently active for the direct installation of a carbazole block into BTD derivatives **4a**–**d**. The reactions have been accomplished under heating in dioxane solution in the presence of *t*-BuONa (3 equiv.), affording the desired products **7a**–**d** in acceptable yields (Scheme 6).

**Figure 1:**
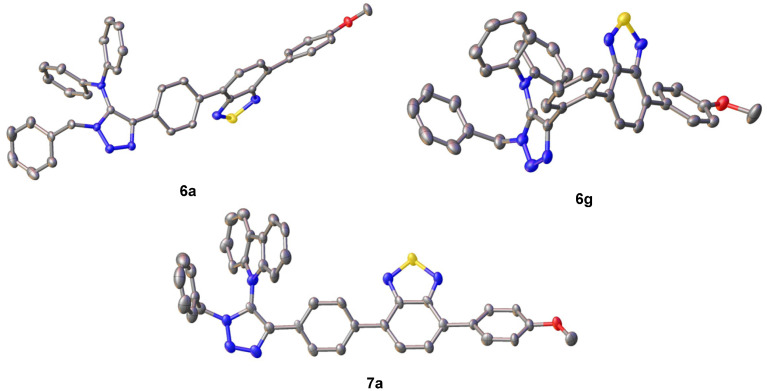
Structure of **6a** (CCDC 2345715), **6g** (CCDC 2345713), and **7a** (CCDC 2345714).

All synthesized compounds isolated in analytically pure form via flash chromatography were fully characterized by means of standard physicochemical methods (see Supplementary Materials). In addition, single crystals of good quality were obtained from **6a**, **6g**, and **7a** for X-ray analysis (Figure 1).

### 2.2. Optical and Electrochemical Investigation

We performed the initial estimation of photophysical properties of each type of the prepared compounds (**4b**, **5f**, **6d**, **6g**, and **7a**). Optical and electrochemical data are collected in Table 1. 

**Figure 2:**
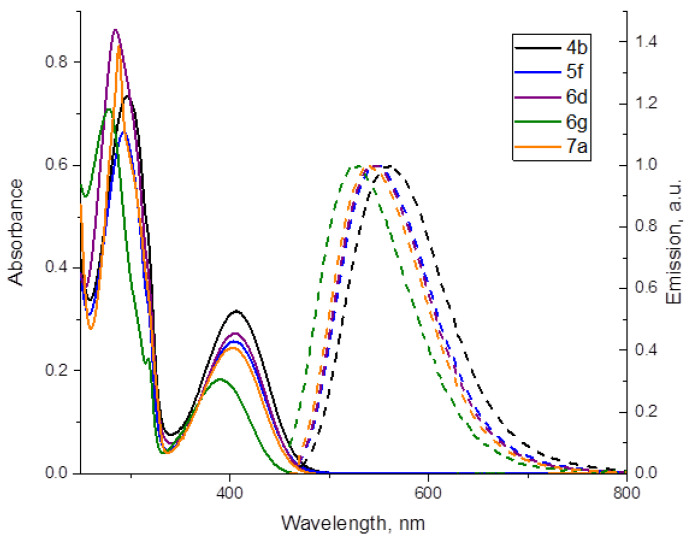
Electronic absorption and emission spectra of **4b**, **5f**, **6d**, **6g**, and **7a** in dichloromethane, C = 2 × 10^−5^ M.

All compounds exhibit intense absorption bands at the edge of the visible region (Figure 2). For all compounds except **6g**, the absorption maxima are almost the same (scatter 404–407 nm). The compounds have strong emissions with maxima at 529–562 nm. Compounds **4b** and **5f** show significant quantum yields (86–89%), but the quantum yields of **6d** and **7a** are close to 100%, making them the most promising candidates for creating light-emitting OLED layers. Compound **6g** has a reduced quantum yield compared to the rest of the series (although still substantial). The differences in the optical properties of **6g** are clearly due to a change in the position of the triazole moiety in the benzene ring.

Voltammograms of the compounds are presented in ESI. All compounds show a reversible reduction in the region of −0.86 V relative to the selected reference silver chloride electrode. This similarity is due to the similar localization and energy of LUMO on the acceptor fragment of benzothiadiazole, common to all compounds. The irreversible oxidation of compounds is observed in the region of 1.2–1.5 V relative to the reference electrode. The differences are due to the different structures of donor substituents.

### 2.3. Electroluminescent Properties of ***6d***

**Figure 3:**
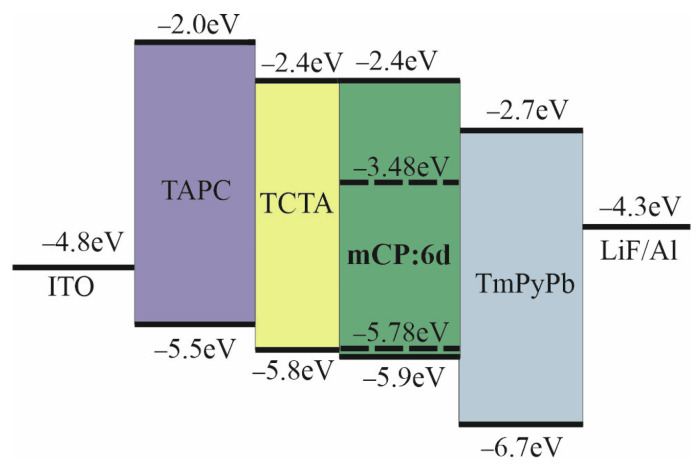
Energy band diagram of the OLED structure with the co-deposited EML layer **6d**/mCP.

Most of the synthesized compounds have a high photoluminescence quantum yield (PLQY) of over 80% in dichloromethane solution (DCM). Since compound **6d** exhibits photoluminescence with the high PLQY of 95%, its electroluminescent properties were investigated in an OLED device with the following structure ITO/TAPC (53 nm)/TCTA (9 nm)/**6d** (15 wt.%):mCP (27 nm)/TPYMB (30 nm)/LiF (1 nm)/Al (80) nm. It contained a double-hole transport layer (HTL) consisting of 1,1-bis[(di-4-tolylamino) phenyl] cyclohexane (TAPC) and 4,4,4-tris(*N*-carbazolyl)triphenylamine (TCTA). The insertion of the thin layer of TCTA improved the hole-injection ability because of the step-wise hole injection from TAPC and TCTA to mCP (Figure 3), thereby ensuring a balance of charge carriers in the light-emitting layer (EML). In addition, it prevents the formation of exciplexes between the TAPC and the EML compounds [59]. 

Tris-(2,4,6-trimethyl-3-(pyridin-3-yl)phenyl) borane (3TPYMB) serves here as an electron transport layer (ETL), ITO is the anode and LiF/Al is the cathode. The light-emitting layer (EML) consists of a wide bandgap host material *N*,*N*-dicarbazolyl-3,5-benzene (mCP) and additive of dye **6d**. EMLs with a dye content in the mCP matrix from 10 to 18 wt.% were investigated and optimal characteristics were obtained for OLED with a dye content of 15 wt.% (Table 2). All layers included in the OLED structures were formed using the method of thermal vacuum evaporation (TVE). OLED with EML based on pure **6d** compound has also been produced. It demonstrated maximum brightness and a current efficiency two times lower than that of structures with co-deposited EML layers. Apparently, this is due to the concentration quenching of excited states.

**Figure 4:**
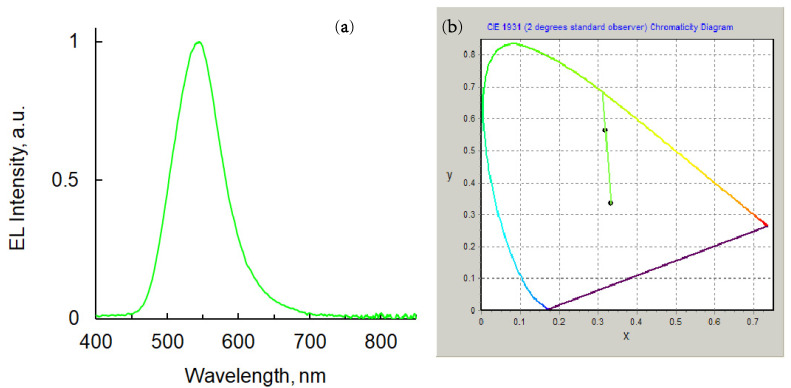
(**a**) Normalized EL spectrum; (**b**) the CIE chromaticity diagram of OLED based on co-deposited EML layer **6d**/mCP.

The EL band of the device with a co-deposited **6d**/mCP EML layer has a maximum at 555 nm (Figure 4a) and is shifted by 9 nm to longer wavelengths relative to the PL spectrum in a DCM solution (Table 1). The OLED emission color is green-white unsaturated and the CIE chromaticity coordinates do not lie on the locus boundary (Figure 4b) since the FWHM of the EL band is 79 nm. The broad PL and EL spectra arise as a result of charge transfer (CT) between the donor and acceptor fragments of dye **6d**.

**Figure 5:**
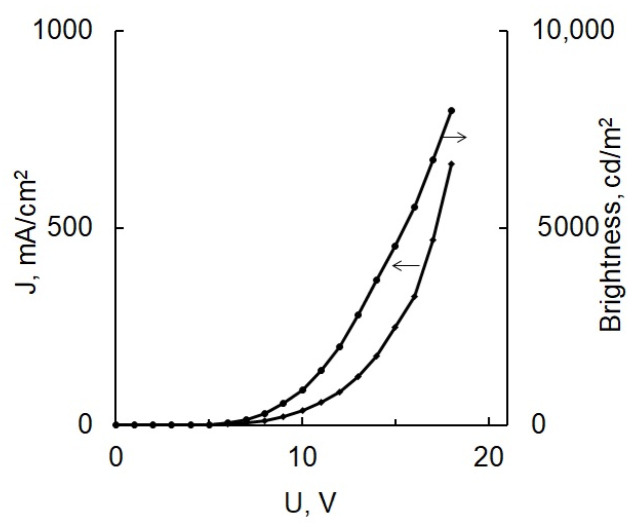
Current density–voltage dependence and voltage–brightness characteristics of the OLED based on EML layer **6d**/mCP.

As can be clearly seen from Figure 3, the HOMO and LUMO energy levels of compound **6d** measured by CVA are well matched to the levels of mCP, which provides a balanced injection of electrons and holes into the EML and the onset voltage does not exceed 4 V. Since the HOMO levels of **6d** and mCP are very close to each other, the **6d** molecules are shallow traps and do not interfere with the transport of holes in the light-emitting layer. The maximum brightness of an OLED with the EML layer of **6d**/mCP reaches 8000 cd/m^2^ at an applied voltage of 18 V (Figure 5). The maximum current efficiency of the device reaches a value of 3.29 cd/A (Table 2).

## 3. Materials and Methods

### 3.1. General Information

All reagents were used as purchased from Sigma-Aldrich (Munich, Germany). BTD **1** [33], (THP-Dipp)Pd(cinn)Cl [60], aryl azides [61], benzyl azide, and (3-azidoprop-1-enyl)benzene [62], 2-(azidoethyl)benzene [63], 2,2’-dibromobiphenyl, and 2,2’-dibromo-4,4’,5,5’-tetramethoxybiphenyl [54] were synthesized according to published procedures. Analytical data were in accordance with the literature data. Analytical TLC was performed with Merck silica gel 60 F 254 plates (Darmstadt, Germany); visualization was accomplished with UV light or iodine vapors. Chromatography was carried out using Merck silica gel (Kieselgel 60, 0.063–0.200 mm) and petroleum ether/ethyl acetate as an eluent. The ^1^H and ^13^C NMR spectra were recorded on a Varian Inova 400 spectrometer (Varian, Palo Alto, CA, USA) operating at 400 and 101 MHz, respectively. Chemical shifts are given in ppm using residual solvent signals as internal standards. High-resolution mass spectra were recorded on an LCMS-9030 device (Shimadzu, Japan) by electrospray ionization–mass spectrometry (ESI-MS). 

### 3.2. Preparation and Characterization of Starting Compounds ***2***, ***3a*** and ***3b***

*4-(4-Methoxyphenyl)-7-(4,4,5,5-tetramethyl-1,3,2-dioxaborolan-2-yl)benzo[c][1,2,5]thiadiazole* (**2**). Under argon in a Schlenk tube with a magnetic stirring bar, **1** (321 mg, 1.0 mmol, 1.0 equiv.), KOAc (294 mg, 3.0 mmol, 3 equiv.) and bis(pinacolato)diboron (279 mg, 1.1 mmol, 1.1 equiv.) were added followed by dry dioxane (15 mL). The solution was degassed by argon before adding PdCl_2_(dppf) (14 mg, 0.02 equiv.). Then, the reaction mixture was stirred at 95 °C (oil bath temperature) for 24 h. After cooling to room temperature, the mixture was poured into water and extracted with dichloromethane (3 × 10 mL). The combined organic phases were washed with brine, dried over anhydrous MgSO_4_, filtered, and concentrated under reduced pressure. Purification by chromatography (eluent–hexane: ethyl acetate 5:1) gave **2** (233 mg, 63%) as a white solid. ^1^H NMR (400 MHz, chloroform-d) δ 8.23 (d, *J* = 7.0 Hz, 1H), 7.91 (d, *J* = 8.3 Hz, 2H), 7.64 (d, *J* = 7.0 Hz, 1H), 7.04 (d, *J* = 8.3 Hz, 2H), 3.86 (s, 3H), 1.44 (s, 12H). ^13^C NMR (101 MHz, chloroform-d) δ 160.1, 158.2, 153.4, 139.3, 136.9, 130.7, 129.8, 126.3, 114.1, 84.3, 55.4, 25.0, missing one carbon (C-B) due to interaction with the boron atom. HRMS (ESI) of C_19_H_21_BN_2_O_3_S, *m*/*z*: calcd for [M+H]^+^: 369.1442, found: 369.1448.
*2-(4-(7-(4-Methoxyphenyl)benzo[c][1,2,5]thiadiazol-4-yl)phenyl)acetonitrile* (**3a**). The title compound was synthesized according to the literature procedure [33] from **1** and 2-(4-(4,4,5,5-tetramethyl-1,3,2-dioxaborolan-2-yl)phenyl)acetonitrile as a yellow solid (67% yield). ^1^H NMR (400 MHz, chloroform-d) δ 7.98 (d, *J* = 8.3 Hz, 2H), 7.94 (d, *J* = 8.9 Hz, 2H), 7.75 (d, *J* = 4.1 Hz, 2H), 7.51 (d, *J* = 8.4 Hz, 2H), 7.09 (d, *J* = 8.9 Hz, 2H), 3.90 (s, 3H), 3.85 (s, 2H). ^13^C NMR (101 MHz, chloroform-d) δ 160.1, 154.3, 154.1, 137.5, 133.6, 131.7, 130.6, 130.0, 129.9, 128.4, 128.3, 127.3, 117.8, 114.3, 110.1, 55.5, 23.6. HRMS (ESI) of C_21_H_15_N_3_OS, *m*/*z*: calcd for [M+H]^+^ 358.1008, found: 358.1009.
*2-(2-(7-(4-Methoxyphenyl)benzo[c][1,2,5]thiadiazol-4-yl)phenyl)acetonitrile* (**3b**). The title compound was synthesized according to the literature procedure [33] from **2** and 2-(2-bromophenyl)acetonitrile as a yellow solid (66% yield). ^1^H NMR (400 MHz, chloroform-d) δ 7.96 (d, *J* = 8.7 Hz, 2H), 7.77 (d, *J* = 7.2 Hz, 1H), 7.68 (d, *J* = 7.6 Hz, 1H), 7.63 (d, *J* = 7.2 Hz, 1H), 7.54 (t, *J* = 7.5 Hz, 1H), 7.49 (t, *J* = 7.2 Hz, 1H), 7.44 (d, *J* = 7.3 Hz, 1H), 7.11 (d, *J* = 8.7 Hz, 2H), 3.91 (s, 3H), 3.66 (s, 2H). ^13^C NMR (101 MHz, chloroform-d) δ 160.2, 154.3, 153.7, 137.4, 134.3, 131.3, 131.3, 130.7, 130.5, 129.6, 129.4, 129.2, 129.1, 128.5, 127.1, 118.1, 114.3, 55.6, 22.7. HRMS (ESI) of C_21_H_15_N_3_OS, *m*/*z*: calcd for [M+H]^+^ 358.1009, found: 358.1005.

### 3.3. Preparation and Characterization of Novel Compounds

#### 3.3.1. General Procedure A for Preparation of BTD-Containing 5-Amino-1,2,3-triazoles **4a**–**g** via Dipolar Azide–Nitrile Cycloaddition (DCR)

A 25 mL round-bottom flask, equipped with a magnetic stir bar was charged with 3 mmol of **3a** or **3b**, 3.0 equiv. of corresponding azide, DMSO (8 mL). The resulting mixture was placed into a water bath (room temperature) and 0.5 equiv. of powdered potassium tert-butoxide was added portionwise. The reaction mixture was allowed to stir for 3 h at 70 °C. Upon completion, the mixture was poured into water and extracted with dichloromethane (3 × 10 mL). The combined organic phases were washed with brine, dried over MgSO_4_, filtered, and concentrated under reduced pressure. Purification by chromatography (eluent–hexane: ethyl acetate 1:1) yielded an analytically pure product.

*1-Benzyl-4-(4-(7-(4-methoxyphenyl)benzo[c][1,2,5]thiadiazol-4-yl)phenyl)-1H-1,2,3-triazol-5-amine* (**4a**). Compound **4a** was synthesized according to the general procedure A (yield 94%) as a yellow solid. ^1^H NMR (400 MHz, chloroform-d) δ 8.00 (d, *J* = 8.2 Hz, 2H), 7.89 (d, *J* = 8.7 Hz, 2H), 7.79 (d, *J* = 8.2 Hz, 2H), 7.75 (d, *J* = 7.4 Hz, 1H), 7.69 (d, *J* = 7.2 Hz, 1H), 7.39–7.31 (m, 4H), 7.24 (d, *J* = 5.4 Hz, 1H), 7.05 (d, *J* = 8.5 Hz, 2H), 5.45 (s, 2H), 3.86 (s, 3H), 3.76 (s, 2H). ^13^C NMR (101 MHz, chloroform-d) δ 160.0, 154.3, 154.2, 137.7, 136.1, 134.2, 133.1, 132.1, 131.6, 130.6, 130.0, 129.9 (d, *J* = 7.8 Hz), 129.4, 128.8, 128.2, 127.4, 127.4, 125.8, 114.2, 55.5, 50.9. HRMS (ESI) of C_28_H_23_N_6_OS, *m*/*z*: calcd for [M+H]^+^ 491.1649, found: 491.1652.
*4-(4-(7-(4-Methoxyphenyl)benzo[c][1,2,5]thiadiazol-4-yl)phenyl)-1-phenethyl-1H-1,2,3-triazol-5-amine* (**4b**). Compound **4b** was synthesized according to the general procedure A (yield 64%) as a yellow solid. ^1^H NMR (400 MHz, chloroform-d) δ 8.03 (d, *J* = 8.5 Hz, 2H), 7.94 (d, *J* = 8.8 Hz, 2H), 7.82–7.74 (m, 4H), 7.31 (q, *J* = 5.9 Hz, 3H), 7.14–7.08 (m, 4H), 4.46 (t, *J* = 6.6 Hz, 2H), 3.91 (s, 3H), 3.24 (t, *J* = 6.5 Hz, 2H), 3.09 (s, 2H). ^13^C NMR (101 MHz, chloroform-d) δ 160.0, 154.3, 154.2, 138.0, 137.8, 136.1, 133.1, 132.2, 131.8, 131.0, 130.6, 130.0, 129.8, 129.2, 129.1, 128.2, 127.4, 126.0, 114.3, 55.5, 48.4, 36.5. HRMS (ESI) of C_29_H_24_N_6_OS, *m*/*z*: calcd for [M+H]^+^ 505.1805, found: 505.1806.
*1-Cinnamyl-4-(4-(7-(4-methoxyphenyl)benzo[c][1,2,5]thiadiazol-4-yl)phenyl)-1H-1,2,3-triazol-5-amine* (**4c**). Compound **4c** was synthesized according to the general procedure A (yield 62%) as a red solid. ^1^H NMR (400 MHz, chloroform-d) δ 8.07 (d, *J* = 8.3 Hz, 2H), 7.94 (d, *J* = 8.8 Hz, 2H), 7.87 (d, *J* = 8.3 Hz, 2H), 7.81 (d, *J* = 7.3 Hz, 1H), 7.76 (s, 1H), 7.41–7.38 (m, 2H), 7.36–7.33 (m, 2H), 7.32–7.30 (m, 1H), 7.09 (d, *J* = 8.8 Hz, 2H), 6.65 (d, *J* = 15.9 Hz, 1H), 6.40–6.34 (m, 1H), 5.10 (d, *J* = 6.2 Hz, 2H), 4.00 (s, 2H), 3.90 (s, 3H). ^13^C NMR (101 MHz, chloroform-d) δ 160.0, 154.3, 154.2, 137.9, 136.2, 135.5, 134.4, 133.1, 132.2, 131.7, 131.0, 130.6, 130.0, 129.9, 128.9, 128.7, 128.2, 127.5, 126.8, 125.9, 121.9, 114.3, 55.5, 49.4. HRMS (ESI) of C_30_H_24_N_6_OS, *m*/*z*: calcd for [M+H]_+_ 517.1805, found: 517.1807.
*1-(4-Methoxyphenyl)-4-(4-(7-(4-methoxyphenyl)benzo[c][1,2,5]thiadiazol-4-yl)phenyl)-1H-1,2,3-triazol-5-amine* (**4d**). Compound **4d** was synthesized according to the general procedure A (yield 81%) as a red solid. ^1^H NMR (400 MHz, DMSO-*d_6_*) δ 8.11 (d, *J* = 8.4 Hz, 2H), 8.00 (t, *J* = 7.9 Hz, 5H), 7.91 (d, *J* = 7.2 Hz, 1H), 7.54 (d, *J* = 8.6 Hz, 2H), 7.20–7.10 (m, 4H), 5.83 (s, 2H), 3.86 (s, 6H). ^13^C NMR (101 MHz, DMSO-*d_6_*) δ 159.6, 159.5, 153.5, 153.4, 139.7, 134.2, 132.6, 132.0, 131.7, 131.2, 130.3, 129.3, 129.2, 129.2, 129.0, 128.0, 128.80, 127.6, 127.5, 126.6, 124.7, 55.6, 55.3. HRMS (ESI) of C_28_H_22_N_6_O_2_S, *m*/*z*: calcd for [M+H]^+^ 507.1598, found: 507.1593.
*4-(4-(7-(4-Methoxyphenyl)benzo[c][1,2,5]thiadiazol-4-yl)phenyl)-1-p-tolyl-1H-1,2,3-triazol-5-amine* (**4e**). Compound **4e** was synthesized according to the general procedure A (yield 75%) as a red solid. ^1^H NMR (400 MHz, chloroform-d) δ 8.08 (d, *J* = 7.8 Hz, 2H), 7.93 (t, *J* = 6.6 Hz, 4H), 7.81 (d, *J* = 7.2 Hz, 1H), 7.74 (d, *J* = 7.4 Hz, 1H), 7.50 (d, *J* = 7.8 Hz, 2H), 7.38 (d, *J* = 7.8 Hz, 2H), 7.09 (d, *J* = 8.2 Hz, 2H), 4.15 (s, 2H), 3.90 (s, 3H), 2.46 (s, 3H). ^13^C NMR (101 MHz, chloroform-d) δ 160.0, 154.3, 154.2, 139.9, 137.9, 136.0, 133.1, 132.6, 132.1, 131.6, 130.6, 130.6, 129.9, 129.4, 128.2, 127.5, 125.7, 124.5, 114.2, 110.2, 55.5, 21.4. HRMS (ESI) of C_28_H_23_N_6_OS, *m*/*z*: calcd for [M+H]^+^ 491.1649, found: 491.1654.
*1-Benzyl-4-(2-(7-(4-methoxyphenyl)benzo[c][1,2,5]thiadiazol-4-yl)phenyl)-1H-1,2,3-triazol-5-amine* (**4f**). Compound **4f** was synthesized according to the general procedure A (yield 84%) as a yellow solid. ^1^H NMR (400 MHz, chloroform-d) δ 7.85 (d, *J* = 8.5 Hz, 2H), 7.70 (d, *J* = 8.5 Hz, 1H), 7.64 (d, *J* = 8.5 Hz, 1H), 7.53 (d, *J* = 5.2 Hz, 3H), 7.48 (d, *J* = 7.4 Hz, 1H), 7.19 (d, *J* = 4.8 Hz, 3H), 7.08 (d, *J* = 8.6 Hz, 2H), 6.87 (d, *J* = 7.4 Hz, 2H), 5.19 (s, 2H), 3.91 (s, 3H), 2.83 (s, 2H). ^13^C NMR (101 MHz, chloroform-d) δ 160.0, 154.4, 153.7, 137.4, 136.7, 134.1, 133.0, 132.3, 131.3, 131.0, 130.6, 130.5, 130.3, 129.7, 129.2, 128.9, 128.4, 128.4, 127.2, 126.9, 114.2, 55.5, 50.6. HRMS (ESI) of C_28_H_22_N_6_OS, *m*/*z*: calcd for [M+H]^+^ 491.1649, found: 491.1644.
*4-(2-(7-(4-Methoxyphenyl)benzo[c][1,2,5]thiadiazol-4-yl)phenyl)-1-p-tolyl-1H-1,2,3-triazol-5-amine* (**4g**). Compound **4g** was synthesized according to the general procedure A (yield 71%) as a red solid. ^1^H NMR (400 MHz, chloroform-d) δ 7.91 (d, *J* = 7.1 Hz, 2H), 7.76 (d, *J* = 7.0 Hz, 1H), 7.68 (d, *J* = 7.0 Hz, 1H), 7.62 (dd, *J* = 7.3, 1.5 Hz, 1H), 7.59–7.53 (m, 3H), 7.21 (d, *J* = 7.5 Hz, 2H), 7.15 (d, *J* = 6.9 Hz, 2H), 7.06 (d, *J* = 7.2 Hz, 2H), 3.88 (s, 3H), 3.33 (s, 2H), 2.35 (s, 3H). ^13^C NMR (101 MHz, chloroform-d) δ 160.0, 154.6, 153.9, 139.4, 137.7, 136.9, 133.1, 132.7, 132.4, 131.5, 130.9, 130.6, 130.4, 129.8, 128.9, 128.4, 127.3, 124.0, 114.2, 55.5, 21.3. HRMS (ESI) of C_28_H_23_N_6_OS, *m*/*z*: calcd for [M+H]^+^ 491.1649, found: 491.1645.

#### 3.3.2. General Procedure B for Preparation of *N*-Monosubstituted Arylamino-1,2,3-triazole- 2,1,3-Benzothiadiazoles **5a**–**f** via BHA Reaction

A screw-cap vial equipped with a magnetic stir bar charged with 0.2 mmol of **4** and (het)arylbromide (5.0 equiv.), dry 1,4-dioxane (1.0 mL), (THP-Dipp)Pd(cinn)Cl (5 mol%), and sodium *tert*-butoxide (3.0 equiv.) was replaced into a preheated oil bath 110 °C and allowed to stir for 18 h at this temperature. After cooling to room temperature, the reaction mixture was poured into water and extracted with dichloromethane (3 × 10 mL). The combined organic phases were washed with brine, dried over MgSO_4_, filtered, and concentrated under reduced pressure. Purification by chromatography (eluent–hexane: ethyl acetate 2:1) yielded an analytically pure product.

*1-Benzyl-4-(4-(7-(4-methoxyphenyl)benzo[c][1,2,5]thiadiazol-4-yl)phenyl)-N-phenyl-1H-1,2,3-triazol-5-amine* (**5a**). Compound **5a** was synthesized according to the general procedure B (yield 91%) as a yellow solid. ^1^H NMR (400 MHz, chloroform-d) δ 7.99 (d, *J* = 8.2 Hz, 2H), 7.95–7.88 (m, 4H), 7.73 (d, *J* = 7.4 Hz, 1H), 7.69 (d, *J* = 7.4 Hz, 1H), 7.35–7.28 (m, 3H), 7.25–7.17 (m, 4H), 7.07 (d, *J* = 8.7 Hz, 2H), 6.90 (t, *J* = 7.4 Hz, 1H), 6.57 (d, *J* = 7.9 Hz, 2H), 5.39 (s, 2H), 5.18 (s, 1H), 3.89 (s, 3H). ^13^C NMR (101 MHz, chloroform-d) δ 160.0, 154.3, 154.1, 143.6, 140.9, 137.1, 134.7, 133.2, 132.1, 131.8, 130.6, 130.2, 130.0, 129.6, 129.1, 128.6, 128.2, 128.0, 127.4, 126.1, 120.8, 114.3, 114.2, 55.5, 51.6. HRMS (ESI) of C_34_H_26_N_6_OS, *m*/*z*: calcd for [M+H]^+^ 567.1962, found: 567.1962.
*1-Benzyl-4-(4-(7-(4-methoxyphenyl)benzo[c][1,2,5]thiadiazol-4-yl)phenyl)-N-o-tolyl-1H-1,2,3-triazol-5-amine* (**5b**). Compound **5b** was synthesized according to the general procedure B (yield 61%) as a yellow solid. ^1^H NMR (400 MHz, chloroform-d) δ 7.97 (d, *J* = 2.3 Hz, 4H), 7.92 (d, *J* = 8.6 Hz, 2H), 7.76 (d, *J* = 7.5 Hz, 1H), 7.71 (d, *J* = 7.3 Hz, 1H), 7.33–7.30 (m, 3H), 7.18 (t, *J* = 7.6 Hz, 3H), 7.07 (d, *J* = 8.6 Hz, 2H), 7.02 (t, *J* = 8.6 Hz, 1H), 6.86 (t, *J* = 7.5 Hz, 1H), 6.32 (d, *J* = 8.0 Hz, 1H), 5.37 (s, 2H), 4.87 (s, 1H), 3.89 (s, 3H), 2.17 (s, 3H). ^13^C NMR (101 MHz, chloroform-d) δ 160.0, 154.3, 154.1, 141.5, 140.7, 137.1, 134.7, 133.2, 132.1, 131.1, 130.6, 130.3, 130.0, 129.6, 129.1, 128.6, 128.2, 127.9, 127.7, 127.4, 126.1, 123.4, 120.9, 114.3, 112.9, 55.6, 51.8, 17.5. HRMS (ESI) of C_35_H_28_N_6_OS, *m*/*z*: calcd for [M+H]^+^ 581.2118, found: 581.2117.
*1-Benzyl-4-(4-(7-(4-methoxyphenyl)benzo[c][1,2,5]thiadiazol-4-yl)phenyl)-N-p-tolyl-1H-1,2,3-triazol-5-amine* (**5c**). Compound **5c** was synthesized according to the general procedure B (yield 88%) as a yellow solid. ^1^H NMR (400 MHz, chloroform-d) δ 8.02 (d, *J* = 8.3 Hz, 2H), 7.96 (d, *J* = 8.4 Hz, 2H), 7.92 (d, *J* = 8.6 Hz, 2H), 7.76 (d, *J* = 7.4 Hz, 1H), 7.72 (d, *J* = 7.4 Hz, 1H), 7.32 (dd, *J* = 4.8, 1.8 Hz, 3H), 7.22 (dd, *J* = 6.5, 2.8 Hz, 2H), 7.08 (d, *J* = 8.6 Hz, 2H), 7.03 (d, *J* = 8.1 Hz, 2H), 6.50 (d, *J* = 8.2 Hz, 2H), 5.39 (s, 2H), 4.95 (s, 1H), 3.89 (s, 3H), 2.28 (s, 3H). ^13^C NMR (101 MHz, chloroform-d) δ 160.0, 154.3, 154.1, 141.2, 140.6, 137.0, 134.8, 133.1, 132.3, 132.1, 130.5, 130.4, 129.5, 129.0, 128.5, 128.2, 128.0, 127.4, 126.1, 120.7, 114.4, 114.2, 55.5, 51.5, 20.6. HRMS (ESI) of C_35_H_28_N_6_OS, *m*/*z*: calcd for [M+H]^+^ 581.2118, found: 581.2114.
*N-(1-Benzyl-4-(4-(7-(4-methoxyphenyl)benzo[c][1,2,5]thiadiazol-4-yl)phenyl)-1H-1,2,3-triazol-5-yl)pyridin-3-amine* (**5d**). Compound **5d** was synthesized according to the general procedure B (yield 55%) as a yellow solid. ^1^H NMR (400 MHz, chloroform-d) δ 8.06 (s, 2H), 7.91–7.86 (m, 5H), 7.66–7.62 (m, 2H), 7.54–7.43 (m, 1H), 7.25–7.21 (m, 3H), 7.19 (s, 2H), 7.05 (d, *J* = 8.5 Hz, 2H), 7.00–6.95 (m, 1H), 6.58 (d, *J* = 8.3 Hz, 1H), 6.16 (s, 1H), 5.41 (s, 2H), 3.88 (s, 3H). ^13^C NMR (101 MHz, chloroform-d) δ 160.0, 154.2, 154.0, 141.4, 141.1, 140.4, 137.3, 136.9, 134.3, 133.2, 132.1, 131.8, 130.7, 130.6, 129.7, 129.6, 129.1, 128.7, 128.3, 128.0, 127.3, 126.1, 124.2, 120.4, 114.2, 55.5, 51.8. HRMS (ESI) of C_33_H_25_N_7_OS, *m*/*z*: calcd for [M+H]^+^ 568.1914, found: 568.1906.
*1-Benzyl-N-(4-methoxyphenyl)-4-(4-(7-(4-methoxyphenyl)benzo[c][1,2,5]thiadiazol-4-yl)phenyl)-1H-1,2,3-triazol-5-amine* (**5e**). Compound **5e** was synthesized according to the general procedure B (yield 59%) as a yellow solid. ^1^H NMR (400 MHz, chloroform-d) δ 8.01–7.93 (m, 4H), 7.91 (d, *J* = 8.7 Hz, 2H), 7.74 (d, *J* = 7.4 Hz, 1H), 7.69 (d, *J* = 7.3 Hz, 1H), 7.32–7.29 (m, 3H), 7.21 (dd, *J* = 6.8, 2.8 Hz, 2H), 7.07 (d, *J* = 8.6 Hz, 2H), 6.77 (d, *J* = 8.8 Hz, 2H), 6.53 (d, *J* = 8.8 Hz, 2H), 5.38 (s, 2H), 5.05 (s, 1H), 3.89 (s, 3H), 3.75 (s, 3H). ^13^C NMR (101 MHz, chloroform-d) δ 160.0, 154.4, 154.3, 154.1, 140.2, 137.1, 137.1, 134.7, 133.2, 132.9, 132.1, 130.6, 130.0, 129.6, 129.0, 128.6, 128.2, 128.0, 127.4, 126.2, 116.0, 115.3, 114.3, 55.8, 55.5, 51.6. HRMS (ESI) of C_35_H_28_N_6_O_2_S, *m*/*z*: calcd for [M+H]^+^ 597.2067, found: 597.2064.
*4-(4-(7-(4-Methoxyphenyl)benzo[c][1,2,5]thiadiazol-4-yl)phenyl)-1-phenethyl-N-p-tolyl-1H-1,2,3-triazol-5-amine* (**5f**). Compound **5f** was synthesized according to the general procedure B (yield 53%) as a yellow solid. ^1^H NMR (400 MHz, chloroform-d) δ 7.96–7.90 (m, 6H), 7.72 (q, *J* = 7.4 Hz, 2H), 7.34–7.30 (m, 3H), 7.08 (d, *J* = 8.8 Hz, 2H), 7.06–7.03 (m, 2H), 6.98 (d, *J* = 8.0 Hz, 2H), 6.33 (d, *J* = 8.4 Hz, 2H), 4.40 (t, *J* = 6.8 Hz, 2H), 4.21 (s, 1H), 3.89 (s, 3H), 3.20 (t, *J* = 6.8 Hz, 2H), 2.24 (s, 3H). ^13^C NMR (101 MHz, chloroform-d) δ 160.0, 154.3, 154.1, 141.2, 140.1, 138.0, 136.9, 133.1, 132.5, 132.2, 130.6, 130.4, 130.4, 130.0, 129.9, 129.5, 129.2, 129.0, 128.2, 127.4, 127.4, 126.2, 114.2, 114.1, 55.5, 48.9, 36.8, 20.6. HRMS (ESI) of C_36_H_30_N_6_OS, *m*/*z*: calcd for [M+H]^+^ 595.2275, found: 595.2272.

#### 3.3.3. General Procedure C for Preparation *N*,*N*-Disubstituted-arylamino-1,2,3-triazole- 2,1,3-Benzothiadiazoles **6a**–**l** and **7a**–**d** via BHA Reaction

A screw-cap vial equipped with a magnetic stir bar was charged with 0.2 mmol of **4,** corresponding aryl-Br (5 equiv.) or 2,2′-dibromobiphenyl (1.0 equiv.), dry 1,4-dioxane (1.0 mL), (THP-Dipp)Pd(cinn)Cl (5 mol%), *t*-Bu_3_-HBF_4_ (10 mol.%), and sodium *tert*-butoxide (3.0 equiv.) was replaced into a preheated oil bath 110 °C (or 150 °C for solvent-free procedure) and allowed to stir for 18 h at this temperature. After cooling to room temperature, the reaction mixture was poured into water and extracted with dichloromethane (3 × 10 mL). The combined organic phases were washed with brine, dried over MgSO_4_, filtered, and concentrated under reduced pressure. Purification by chromatography (eluent–hexane: ethyl acetate 2:1) yielded an analytically pure product.

*1-Benzyl-4-(4-(7-(4-methoxyphenyl)benzo[c][1,2,5]thiadiazol-4-yl)phenyl)-N,N-diphenyl-1H-1,2,3-triazol-5-amine* (**6a**). Compound **6a** was synthesized according to the general procedure C (yield 96%) as a yellow solid. ^1^H NMR (400 MHz, chloroform-d) δ 8.0–7.9 (m, 3H), 7.9–7.9 (m, 3H), 7.7 (d, *J* = 4.0 Hz, 2H), 7.2–7.2 (m, 3H), 7.2–7.1 (m, 6H), 7.1 (d, *J* = 8.8 Hz, 2H), 7.0 (t, *J* = 7.4 Hz, 2H), 6.9 (d, *J* = 7.6 Hz, 4H), 5.2 (s, 2H), 3.9 (s, 3H). ^13^C NMR (101 MHz, chloroform-d) δ 160.0, 154.3, 154.1, 143.9, 140.6, 137.0, 135.5, 134.3, 133.1, 132.1, 130.5, 130.0, 129.7, 129.3, 129.1, 128.8, 128.4, 128.2, 127.4, 126.1, 123.5, 120.7, 114.2, 55.5, 51.8. HRMS (ESI) of C_40_H_30_N_6_OS, *m*/*z*: calcd for [M+H]^+^ 643.2275, found: 643.2275.
*4-(4-(7-(4-Methoxyphenyl)benzo[c][1,2,5]thiadiazol-4-yl)phenyl)-N,N-diphenyl-1-p-tolyl-1H-1,2,3-triazol-5-amine* (**6b**). Compound **6b** was synthesized according to the general procedure C (yield 67%) as a yellow solid. ^1^H NMR (400 MHz, chloroform-d) δ 8.06 (d, *J* = 8.4 Hz, 2H), 7.94–7.89 (m, 4H), 7.73 (d, *J* = 7.4 Hz, 1H), 7.69 (d, *J* = 7.3 Hz, 1H), 7.25–7.23 (m, 2H), 7.17 (t, *J* = 7.9 Hz, 4H), 7.10 (d, *J* = 8.2 Hz, 2H), 7.06 (d, *J* = 8.7 Hz, 2H), 6.97 (d, *J* = 8.3 Hz, 6H), 3.87 (s, 3H), 2.32 (s, 3H). ^13^C NMR (101 MHz, chloroform-d) δ 160.0, 154.3, 154.1, 144.2, 140.6, 139.7, 137.2, 135.7, 133.2, 133.2, 132.1, 130.6, 130.0, 129.8, 129.6, 129.5, 128.3, 127.4, 126.2, 124.5, 123.4, 120.7, 114.2, 55.5, 21.3. HRMS (ESI) of C_40_H_30_N_6_OS, *m*/*z*: calcd for [M+H]+ 643.2275, found: 643.2267; calcd for [M+Na]^+^ 665.2094, found: 665.2092.
*1-(4-Methoxyphenyl)-4-(4-(7-(4-methoxyphenyl)benzo[c][1,2,5]thiadiazol-4-yl)phenyl)-N,N-diphenyl-1H-1,2,3-triazol-5-amine* (**6c**). Compound **6c** was synthesized according to the general procedure C (yield 62%) as a yellow solid. ^1^H NMR (400 MHz, chloroform-d) δ 8.06 (d, *J* = 8.1 Hz, 2H), 7.95–7.90 (m, 4H), 7.76–7.69 (m, 2H), 7.25 (s, 2H), 7.18 (t, *J* = 7.8 Hz, 4H), 7.07 (d, *J* = 8.4 Hz, 2H), 6.96 (d, *J* = 7.6 Hz, 6H), 6.80 (d, *J* = 8.5 Hz, 2H), 3.88 (s, 3H), 3.78 (s, 3H). ^13^C NMR (101 MHz, chloroform-d) δ 160.4, 160.0, 154.3, 144.2, 137.2, 135.9, 132.1, 130.6, 130.0, 129.7, 129.5, 128.3, 127.4, 126.3, 126.1, 123.4, 120.7, 114.4, 114.3, 55.7, 55.6. HRMS (ESI) of C_40_H_30_N_6_O_2_S, *m*/*z*: calcd for [M+H]^+^ 659.2224, found: 659.2224.
*4-(4-(7-(4-Methoxyphenyl)benzo[c][1,2,5]thiadiazol-4-yl)phenyl)-1-phenethyl-N,N-diphenyl-1H-1,2,3-triazol-5-amine* (**6d**). Compound **6d** was synthesized according to the general procedure C (yield 83%) as a yellow solid. ^1^H NMR (400 MHz, chloroform-d) δ 7.98 (d, *J* = 8.5 Hz, 2H), 7.93–7.87 (m, 4H), 7.70 (q, *J* = 7.3 Hz, 2H), 7.28–7.20 (m, 8H), 7.06 (d, *J* = 8.9 Hz, 2H), 7.01 (d, *J* = 8.5 Hz, 7H), 4.29–4.22 (t, *J* = 7.6 Hz, 2H), 3.87 (s, 3H), 3.02–2.93 (t, *J* = 7.5 Hz, 2H). ^13^C NMR (101 MHz, chloroform-d) δ 160.0, 154.3, 154.1, 144.2, 140.4, 137.2, 137.1, 135.4, 133.1, 132.1, 130.5, 129.9, 129.7, 129.4, 129.0, 128.8, 128.2, 127.4, 127.0, 126.0, 123.6, 120.6, 114.2, 55.5, 49.1, 35.6. HRMS (ESI) of C_41_H_32_N_6_OS, *m*/*z*: calcd for [M+H]^+^ 657.2431, found: 657.2433; calcd for [M+K]^+^ 695.1990, found: 695.1990.
*1-Benzyl-4-(4-(7-(4-methoxyphenyl)benzo[c][1,2,5]thiadiazol-4-yl)phenyl)-N,N-di(p-tolyl)-1H-1,2,3-triazol-5-amine* (**6e**). Compound **6e** was synthesized according to the general procedure C (yield 97%) as a yellow solid. ^1^H NMR (400 MHz, chloroform-d) δ 7.91 (dt, *J* = 24.0, 8.4 Hz, 7H), 7.70 (d, *J* = 4.3 Hz, 2H), 7.24–7.22 (m, 2H), 7.16 (t, *J* = 4.0 Hz, 2H), 7.07 (d, *J* = 8.8 Hz, 2H), 6.96 (d, *J* = 8.2 Hz, 4H), 6.78 (d, *J* = 8.2 Hz, 4H), 5.22 (s, 2H), 3.89 (s, 3H), 2.24 (s, 6H). ^13^C NMR (101 MHz, chloroform-d) δ 160.0, 154.3, 154.1, 141.7, 140.3, 136.9, 135.8, 134.5, 133.0, 132.8, 132.2, 130.5, 130.2, 129.2, 128.7, 128.4, 128.3, 128.1, 127.4, 126.1, 120.6, 114.2, 55.5, 51.7, 20.8. HRMS (ESI) of C_42_H_34_N_6_OS, *m*/*z*: calcd for [M+H]^+^ 671.2588, found: 671.2584; calcd for [M+Na]+ 693.2407, found: 693.2405.
*4-(4-(7-(4-Methoxyphenyl)benzo[c][1,2,5]thiadiazol-4-yl)phenyl)-N,N,1-tri(p-tolyl)-1H-1,2,3-triazol-5-amine* (**6f**). Compound **6f** was synthesized according to the general procedure C (yield 67%) as a yellow solid. ^1^H NMR (400 MHz, chloroform-d) δ 8.08 (d, *J* = 8.2 Hz, 2H), 7.95–7.91 (m, 4H), 7.76 (d, *J* = 7.6 Hz, 1H), 7.71 (d, *J* = 7.5 Hz, 1H), 7.28 (d, *J* = 8.7 Hz, 2H), 7.12 (d, *J* = 8.1 Hz, 2H), 7.09 (s, 2H), 6.97 (d, *J* = 8.5 Hz, 4H), 6.85 (d, *J* = 8.1 Hz, 4H), 3.90 (s, 3H), 2.34 (s, 3H), 2.24 (s, 6H). ^13^C NMR (101 MHz, chloroform-d) δ 160.0, 154.3, 154.1, 141.9, 139.5, 137.1, 135.9, 133.4, 133.1, 132.7, 130.6, 130.2, 129.8, 129.7, 129.5, 128.3, 127.4, 126.2, 124.4, 120.5, 114.3, 110.2, 109.9, 55.6, 21.4, 20.8. HRMS (ESI) of C_42_H_34_N_6_OS, *m*/*z*: calcd for [M+H]^+^ 671.2588, found: 671.2580.
*1-Benzyl-4-(2-(7-(4-methoxyphenyl)benzo[c][1,2,5]thiadiazol-4-yl)phenyl)-N,N-diphenyl-1H-1,2,3-triazol-5-amine* (**6g**). Compound **6g** was synthesized according to the general procedure C (yield 67%) as a yellow solid. ^1^H NMR (400 MHz, chloroform-d) δ 7.94 (d, *J* = 8.7 Hz, 2H), 7.60 (d, *J* = 7.2 Hz, 1H), 7.50 (d, *J* = 7.9 Hz, 1H), 7.44 (d, *J* = 7.2 Hz, 1H), 7.31 (d, *J* = 7.7 Hz, 1H), 7.24 (d, *J* = 5.9 Hz, 3H), 7.10 (d, *J* = 8.8 Hz, 3H), 7.01–6.97 (m, 3H), 6.80 (t, *J* = 7.5 Hz, 4H), 6.76–6.71 (m, 2H), 6.10 (d, *J* = 7.8 Hz, 4H), 4.96 (s, 2H), 3.91 (s, 3H). ^13^C NMR (101 MHz, chloroform-d) δ 159.9, 154.9, 154.0, 143.8, 141.8, 138.1, 136.7, 134.6, 132.9, 132.4, 131.2, 130.6, 130.5, 130.1, 129.4, 128.5, 128.4, 128.2, 127.7, 123.2, 123.1, 120.9, 120.8, 114.3, 55.5, 52.3. HRMS (ESI) of C_40_H_30_N_6_OS, *m*/*z*: calcd for [M+H]^+^ 643.2275, found: 643.2273.
*4-(2-(7-(4-Methoxyphenyl)benzo[c][1,2,5]thiadiazol-4-yl)phenyl)-N,N-diphenyl-1-p-tolyl-1H-1,2,3-triazol-5-amine* (**6h**). Compound **6h** was synthesized according to the general procedure C (yield 71%) as a yellow solid. ^1^H NMR (400 MHz, chloroform-d) δ 7.93 (d, *J* = 8.9 Hz, 2H), 7.63 (d, *J* = 7.3 Hz, 1H), 7.58–7.52 (m, 3H), 7.39 (d, *J* = 6.4 Hz, 1H), 7.22 (d, *J* = 8.3 Hz, 3H), 7.09 (d, *J* = 8.6 Hz, 2H), 6.98 (d, *J* = 8.2 Hz, 2H), 6.86 (t, *J* = 7.6 Hz, 4H), 6.74 (t, *J* = 7.3 Hz, 2H), 6.45 (d, *J* = 7.9 Hz, 4H), 3.90 (s, 3H), 2.22 (s, 3H). ^13^C NMR (101 MHz, chloroform-d) δ 160.0, 155.0, 154.0, 144.0, 141.9, 139.0, 138.0, 133.5, 133.0, 132.6, 131.2, 131.1, 130.6, 130.1, 129.6, 129.3, 128.8, 128.4, 127.9, 126.9, 123.3, 123.0, 120.8, 114.3, 55.6, 21.2. HRMS (ESI) of C_40_H_30_N_6_OS, *m*/*z*: calcd for [M+H]^+^ 643.2275, found: 643.2271.
*1-Benzyl-N,N-bis(4-methoxyphenyl)-4-(4-(7-(4-methoxyphenyl)benzo[c][1,2,5]thiadiazol-4-yl)phenyl)-1H-1,2,3-triazol-5-amine* (**6i**). Compound **6i** was synthesized according to the general procedure C (yield 98%) as a yellow solid. ^1^H NMR (400 MHz, chloroform-d) δ 7.94–7.86 (m, 7H), 7.70 (d, *J* = 5.6 Hz, 2H), 7.25–7.24 (m, 2H), 7.18–7.15 (m, 2H), 7.07 (d, *J* = 9.0 Hz, 2H), 6.79 (d, *J* = 9.0 Hz, 4H), 6.69 (d, *J* = 9.1 Hz, 4H), 5.23 (s, 2H), 3.88 (s, 3H), 3.72 (s, 6H). ^13^C NMR (101 MHz, chloroform-d) δ 160.0, 155.6, 154.3, 154.1, 140.1, 137.8, 136.9, 136.1, 134.6, 133.0, 132.2, 130.5, 130.0, 129.8, 129.2, 128.8, 128.3, 128.3, 128.1, 127.4, 126.1, 121.9, 114.9, 114.2, 110.1, 55.6, 55.5, 51.7. HRMS (ESI) of C_42_H_34_N_6_O_3_S, *m*/*z*: calcd for [M+H]^+^ 703.2486, found: 703.2480; calcd for [M+K]^+^ 741.2045, found: 741.2051.
*1-Benzyl-N,N-bis(4-tert-butylphenyl)-4-(4-(7-(4-methoxyphenyl)benzo[c][1,2,5]thiadiazol-4-yl)phenyl)-1H-1,2,3-triazol-5-amine* (**6j**). Compound **6j** was synthesized according to the general procedure C (yield 64%) as a yellow solid. ^1^H NMR (400 MHz, chloroform-d) δ 7.97 (d, *J* = 8.4 Hz, 2H), 7.91 (d, *J* = 8.7 Hz, 2H), 7.87 (d, *J* = 8.3 Hz, 2H), 7.70 (d, *J* = 2.5 Hz, 2H), 7.17 (dd, *J* = 7.6, 4.1 Hz, 6H), 7.06 (t, *J* = 7.0 Hz, 4H), 6.87 (d, *J* = 8.6 Hz, 4H), 5.27 (s, 2H), 3.89 (s, 3H), 1.26 (s, 18H). ^13^C NMR (101 MHz, chloroform-d) δ 160.0, 154.3, 154.1, 146.1, 141.6, 140.5, 136.9, 135.8, 134.3, 133.1, 132.3, 130.6, 130.0, 129.9, 129.3, 128.7, 128.4, 128.2, 128.1, 127.4, 126.4, 126.1, 55.5, 51.4, 34.4, 31.5. HRMS (ESI) of C_48_H_46_N_6_OS, *m*/*z*: calcd for [M+H]^+^ 755.3527, found: 755.3534; calcd for [M+Na]^+^ 777.3346, found: 777.3333; calcd for [M+K]^+^ 793.3086, found: 793.3090.
*N1-(1-Benzyl-4-(4-(7-(4-methoxyphenyl)benzo[c][1,2,5]thiadiazol-4-yl)phenyl)-1H-1,2,3-triazol-5-yl)-N1-(4-(dimethylamino)phenyl)-N4,N4-dimethylbenzene-1,4-diamine* (**6k**). Compound **6k** was synthesized according to the general procedure C (yield 65%) as a yellow solid. ^1^H NMR (400 MHz, chloroform-d) δ 7.96 (d, *J* = 8.5 Hz, 2H), 7.91 (d, *J* = 8.8 Hz, 2H), 7.86 (d, *J* = 8.3 Hz, 2H), 7.70 (d, *J* = 5.2 Hz, 2H), 7.26–7.24 (m, 3H), 7.20–7.18 (m, 2H), 7.07 (d, *J* = 8.8 Hz, 2H), 6.76 (d, *J* = 9.0 Hz, 4H), 6.55 (d, *J* = 8.9 Hz, 4H), 5.22 (s, 2H), 3.89 (s, 3H), 2.86 (s, 12H). ^13^C NMR (101 MHz, chloroform-d) δ 159.9, 154.3, 154.1, 146.9, 139.8, 136.6, 135.1, 134.9, 132.9, 132.4, 130.5, 130.1, 130.0, 129.2, 128.7, 128.5, 128.2, 128.1, 127.4, 126.2, 121.8, 114.2, 113.9, 55.5, 51.5, 41.2. HRMS (ESI) of C_44_H_40_N_8_OS, *m*/*z*: calcd for [M]^+^ 728.3046, found: 728.3035.
*1-Benzyl-N,N-bis(3,5-dimethylphenyl)-4-(4-(7-(4-methoxyphenyl)benzo[c][1,2,5]thiadiazol-4-yl)phenyl)-1H-1,2,3-triazol-5-amine* (**6l**). Compound **6l** was synthesized according to the general procedure C (yield 58%) as a yellow solid. ^1^H NMR (400 MHz, chloroform-d) δ 7.98 (d, *J* = 8.4 Hz, 2H), 7.93–7.87 (m, 4H), 7.74–7.69 (m, 2H), 7.26–7.23 (m, 3H), 7.20–7.17 (m, 2H), 7.07 (d, *J* = 8.8 Hz, 2H), 6.59 (s, 2H), 6.49 (s, 4H), 5.23 (s, 2H), 3.89 (s, 3H), 2.14 (s, 12H). ^13^C NMR (101 MHz, chloroform-d) δ 160.0, 154.3, 154.1, 144.1, 140.5, 139.3, 139.2, 136.9, 135.9, 134.5, 133.1, 132.3, 130.5, 130.0, 129.9, 129.2, 128.7, 128.5, 128.4, 128.2, 127.4, 126.2, 125.2, 118.8, 114.2, 55.5, 51.7, 21.5. HRMS (ESI) of C_35_H_28_N_6_O_2_S, *m*/*z*: calcd for [M+H]^+^ 699.2901, found: 699.2896; calcd for [M+Na]^+^ 721.2720, found: 721.2714.
*4-(4-(1-Benzyl-5-(9H-carbazol-9-yl)-1H-1,2,3-triazol-4-yl)phenyl)-7-(4-methoxyphenyl) benzo[c][1,2,5]thiadiazole* (**7a**). Compound **7a** was synthesized according to the general procedure C (yield 54%) as a yellow solid. ^1^H NMR (400 MHz, chloroform-d) δ 8.14 (d, *J* = 7.6 Hz, 2H), 7.88 (d, *J* = 8.6 Hz, 2H), 7.78 (d, *J* = 8.3 Hz, 2H), 7.64 (d, *J* = 9.0 Hz, 4H), 7.31 (dt, *J* = 12.8, 7.4 Hz, 4H), 7.05 (d, *J* = 8.8 Hz, 3H), 6.95 (t, *J* = 7.6 Hz, 2H), 6.80 (d, *J* = 7.9 Hz, 2H), 6.71 (d, *J* = 7.5 Hz, 2H), 5.22 (s, 2H), 3.87 (s, 3H). ^13^C NMR (101 MHz, chloroform-d) δ 160.0, 154.2, 154.0, 133.3, 133.2, 131.8, 130.5, 129.9, 129.1, 128.6, 128.4, 128.2, 128.1, 127.6, 127.3, 126.9, 125.9, 124.3, 121.6, 120.8, 114.2, 110.0, 55.5, 52.6. HRMS (ESI) of C_40_H_28_N_6_OS, *m*/*z*: calcd for [M+H]^+^ 641.2118, found: 641.2120.
*4-(4-(1-Benzyl-5-(2,3,6,7-tetramethoxy-9H-carbazol-9-yl)-1H-1,2,3-triazol-4-yl)phenyl)-7-(4-methoxyphenyl)benzo[c][1,2,5]thiadiazole* (**7b**). Compound **7b** was synthesized according to the general procedure C (yield 48%) as a yellow solid. ^1^H NMR (400 MHz, chloroform-d) δ 7.89 (d, *J* = 8.9 Hz, 2H), 7.81 (d, *J* = 8.6 Hz, 2H), 7.68–7.61 (m, 4H), 7.48 (s, 2H), 7.14 (d, *J* = 7.4 Hz, 1H), 7.05 (d, *J* = 8.0 Hz, 4H), 6.82 (d, *J* = 7.6 Hz, 2H), 6.15 (s, 2H), 5.21 (s, 2H), 4.05 (s, 6H), 3.88 (s, 3H), 3.63 (s, 6H). ^13^C NMR (101 MHz, chloroform-d) δ 160.0, 154.2, 154.0, 149.0, 145.7, 142.8, 137.7, 134.4, 133.5, 133.3, 131.8, 130.5, 129.9, 129.7, 129.0, 128.6, 128.5, 128.3, 127.9, 127.3, 125.8, 116.5, 114.2, 102.3, 93.7, 56.8, 56.3, 55.5, 52.4. HRMS (ESI) of C_44_H_36_N_6_O_5_S, *m*/*z*: calcd for [M+H]^+^ 761.2541, found: 761.2534.
*4-(4-(5-(9H-Carbazol-9-yl)-1-(4-methoxyphenyl)-1H-1,2,3-triazol-4-yl)phenyl)-7-(4-methoxyphenyl)benzo[c][1,2,5]thiadiazole* (**7c**). Compound **7c** was synthesized according to the general procedure C (yield 31%) as a yellow solid. ^1^H NMR (400 MHz, chloroform-d) δ 8.13 (d, *J* = 7.3 Hz, 2H), 7.89 (d, *J* = 8.8 Hz, 2H), 7.81 (d, *J* = 8.5 Hz, 2H), 7.68–7.63 (m, 4H), 7.39–7.30 (m, 4H), 7.17 (d, *J* = 9.0 Hz, 2H), 7.09–7.05 (m, 4H), 6.68 (d, *J* = 9.0 Hz, 2H), 3.88 (s, 3H), 3.68 (s, 3H). ^13^C NMR (101 MHz, chloroform-d) δ 160.3, 160.0, 154.3, 154.0, 142.6, 140.2, 137.7, 133.3, 131.9, 130.6, 129.9, 129.6, 129.0, 128.3, 127.3, 127.1, 126.1, 124.5, 124.3, 121.7, 120.9, 114.7, 114.2, 110.2, 55.5, 55.5. HRMS (ESI) of C_40_H_28_N_6_O_2_S, *m*/*z*: calcd for [M+H]^+^ 657.2067, found: 657.2070.
*4-(2-(1-benzyl-5-(9H-carbazol-9-yl)-1H-1,2,3-triazol-4-yl)phenyl)-7-(4-methoxyphenyl)benzo[c][1,2,5]thiadiazole* (**7d**). Compound **7d** was synthesized according to the general procedure C (yield 51%) as a yellow solid. ^1^H NMR (400 MHz, chloroform-d) δ 7.85 (d, *J* = 7.7 Hz, 1H), 7.79 (d, *J* = 8.4 Hz, 2H), 7.75 (d, *J* = 7.8 Hz, 2H), 7.47 (m, 1H), 7.36 (m, 1H), 7.29 (d, *J* = 7.3 Hz, 1H), 7.23 (d, *J* = 7.8 Hz, 1H), 7.07 (d, *J* = 8.7 Hz, 5H), 6.96–6.90 (m, 3H), 6.81 (t, *J* = 7.5 Hz, 2H), 6.43 (d, *J* = 7.8 Hz, 2H), 6.22 (d, *J* = 7.9 Hz, 2H), 5.10 (s, 2H), 3.92 (s, 3H). ^13^C NMR (101 MHz, chloroform-d) δ 159.8, 153.9, 153.3, 138.3, 136.8, 133.3, 132.6, 131.7, 130.8, 130.7, 129.8, 129.5, 128.8, 128.6, 128.4, 128.2, 127.6, 126.5, 126.2, 123.6, 121.3, 120.0, 114.0, 109.0, 55.5, 53.0. HRMS (ESI) of C_40_H_28_N_6_OS, *m*/*z*: calcd for [M+H]+ 641.2118, found: 641.2118.

### 3.4. Optical and Electrochemical Investigation

UV–Vis spectra in dichloromethane solutions were recorded on an Agilent Cary 300 spectrometer (Santa Clara, CA, USA), for the fluorescence spectra, a Cary Eclipse spectrofluorometer has been used. All measured luminescence spectra were corrected for nonuniformity of detector spectral sensitivity. Rhodamine 6G (φ_fl_ 0.95) in ethanol was used as a reference for the luminescence quantum yield measurements. The luminescence quantum yields were calculated using equation:

$$\varphi_{i}=\varphi_{0}\frac{\left(1-10^{-A_{0}}\right)\times S_{i}\times n_{i}^{2}}{\left(1-10^{-A_{i}}\right)\times S_{0}\times n_{0}^{2^{\prime }}}$$

where φ_i_ and φ_0_ are the luminescence quantum yields of the studied solution and the standard compound, respectively; *A*_i_ and *A*_0_ are the absorptions of the studied solution and the standard, respectively; *S*_i_ and *S*_0_ are the areas underneath the curves of the luminescence spectra of the studied solution and the standard, respectively; and *n*_i_ and *n*_0_ are the refractive indices of the solvents for the substance under study and the standard compound (*n*_i_ 1.4242, DCM; *n*_0_ 1.361, EtOH).

### 3.5. Voltammetry Studies

Electrochemical measurements were carried out at 22 °C with a Metrohm Autolab B.V. potentiostat type: PGSTAT128N. Cyclic voltammetry (CV) experiments were performed in three-electrode cell equipped with a glassy carbon (GC) working electrode (disk *d* 2 mm), Ag/AgCl reference electrode, and platinum counter electrode. Compounds were dissolved in degassed dry DCM containing TBAHFP as the supporting electrolyte (0.1 M). Dry argon gas was bubbled through the solutions for 10 min before cyclic voltammetry experiments. The applied scan rate for CV was 200 mV s^−1^. In ESI cyclic voltammograms are given relative to the Ag/AgCl reference electrode. Energies of the frontier molecular orbitals (FMOs) were calculated against ferrocene/ferrocenium (Fc/Fc+) as internal standard as follows: HOMO, eV = –Eox, V–4.8; LUMO, eV = –Ered, V–4.8, since the energy level of ferrocene/ferrocenium is 4.8 eV below the vacuum level [64].

### 3.6. OLED Fabrication and Characterization

OLED devices were fabricated on ITO-coated glass substrates that were pre-cleaned according to the standard procedure as described in [65]. The hole transport, light-emitting, electron transport layers and LiF(1 nm)/Al(80) as cathode were deposited sequentially using thermal vacuum evaporation (TVE) at a residual pressure of 4 × 10^−6^ mbar. The electroluminescence spectra of OLEDs were recorded using an Avantes 2048 fiber-optic spectrofluorimeter (Apeldoorn, The Netherlands). Voltage–current and voltage–brightness characteristics were recorded with a Keithley 2601 SourceMeter (Tektronix, Beaverton, OR, USA), a Keithley 485 picoampermeter, and a TKA-04/3 luxmeter-brightness meter (TKA Scientific Instruments, Saint Petersburg, Russia). Preparations of the OLED samples and the measurements of their spectral and optoelectronic characteristics were performed at room temperature in the argon atmosphere glovebox with maximum oxygen and moisture presence of 10 ppm.

## 4. Conclusions

In summary, we developed an efficient synthetic pathway to new series of 5-(aryl)amino-1,2,3-triazole-containing 2,1,3-benzothiadiazole derivatives. The method is based on 1,3-dipolar azide–nitrile cycloaddition followed by Buchwald–Hartwig cross-coupling to afford the corresponding *N*-aryl and *N*,*N*-diaryl substituted 5-amino-1,2,3-triazolyl 2,1,3-benzothiadiazoles under catalysis with (THP-Dipp)Pd(cinn)Cl complex thus opening the straightforward access to unknown before triazole-linked D-A systems. In addition, the one-pot diarylative Pd-catalyzed heterocyclization has been also performed to demonstrate a quick installation of carbazole donors into BTD structure. The quantum yields of photoluminescence of the obtained derivatives are rather high (54–95%) and depend to a large extent on the strength of the donor component, which was changed in the series from Ph- to diphenylamino- and carbazole moieties. The maximum quantum yield among the obtained compounds is close to 100%. Based on the most photoactive derivative and wide bandgap host material mCP, a light-emitting layer of OLED was made. The device showed a maximum brightness of 8000 cd/m^2^ at an applied voltage of 18 V. The maximum current efficiency of the device reaches a value of 3.29 cd/A.

## Figures and Tables

**Table 1:** The long-wavelength absorption and emission bands, quantum yields, the frontier molecular orbitals (FMOs) energies ^a^ for compounds **4b**, **5f**, **6d**, **6g**, and **7a** in DCM.

Compound	λ_abs_, nm	λ_em_, nm	QY, Air, %	HOMO, eV	LUMO, eV	Gap, eV
**4b**	407	562	89	−5.54	−3.49	2.05
**5f**	405	550	86	−5.59	−3.48	2.11
**6d**	405	546	95	−5.78	−3.48	2.30
**6g**	391	529	54	−5.78	−3.47	2.31
**7a**	404	540	95	−5.83	−3.49	2.34

^a^ Calculated from electrochemical data.

**Table 2:** EL characteristics of the studied OLED with a dye **6d** content in of 15 wt.%.

Light-Emitting Layer	U_on_, V	Max. Brightness, cd/m^2^	Max. Efficiency		CIE		λ_max_ EL, nm
			CE, cd/A	PE, lm/W	x	y	
**6d**/mCP	4.0	8000	3.29	1.78	0.319	0.561	555

## Data Availability

Data are contained within the article and Supplementary Materials.

## Supplementary Materials

The following supporting information can be downloaded at: https://www.mdpi.com/article/10.3390/molecules29092151/s1, The following are available online: copies of ^1^H and ^13^C NMR spectra for all novel compounds, voltammograms for **4b**, **5f**, **6d**, **6g**, **7a**. Deposition Numbers 2345715 (for **6a**), 2345713 (for **6g**), and 2345714 (for **7a**) contain the supplementary crystallographic data for this paper. These data are provided free of charge by the joint Cambridge Crystallographic Data Centre service www.ccdc.cam.ac.uk/structures (accessed on 2 May 2024).
