# Supplementary material for: Synthesis of 5-(Aryl)amino-1,2,3-triazole-containing 2,1,3-Benzothiadiazoles via Azide–Nitrile Cycloaddition Followed by Buchwald–Hartwig Reaction
Source: Molecules. 2024 May 6;29(9):2151. doi: 10.3390/molecules29092151 (PMC11085325; doi:10.3390/molecules29092151)
Supplement: Supplementary file 1 [file molecules-29-02151-s001.zip › molecules-2975255-supplementary.pdf]

Supporting information *for*

Synthesis of novel 5-amino-1,2,3-triazole-2,1,3-benzothiadiazoles and their derivatives via dipolar azide-nitrile cycloaddition and buchwald–hartwig reaction

Pavel S. Gribanov, Anna N. Philippova, Maxim A. Topchiy, Dmitry A. Lypenko, Artem V. Dmitriev, Sergey D. Tokarev, Alexander F. Smol'yakov, Alexey N. Rodionov, Andrey F. Asachenko, and Sergey N. Osipov

Table of contents

NMR spectra S2

Electrochemistry S66

NMR spectra

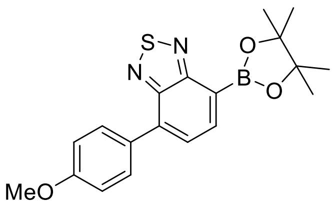

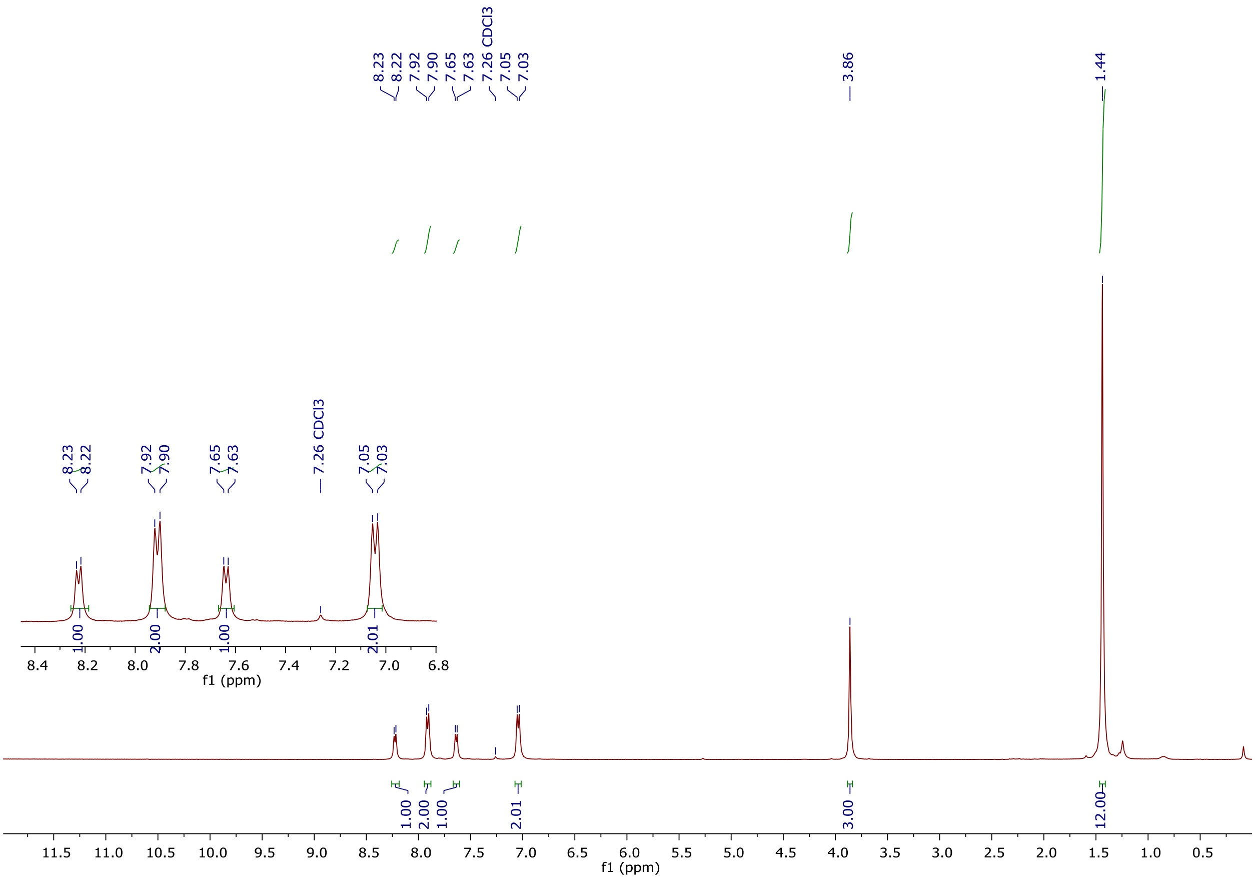

Figure S1. ^{1}H NMR (400 MHz, chloroform-*d*) spectrum of compound **2**

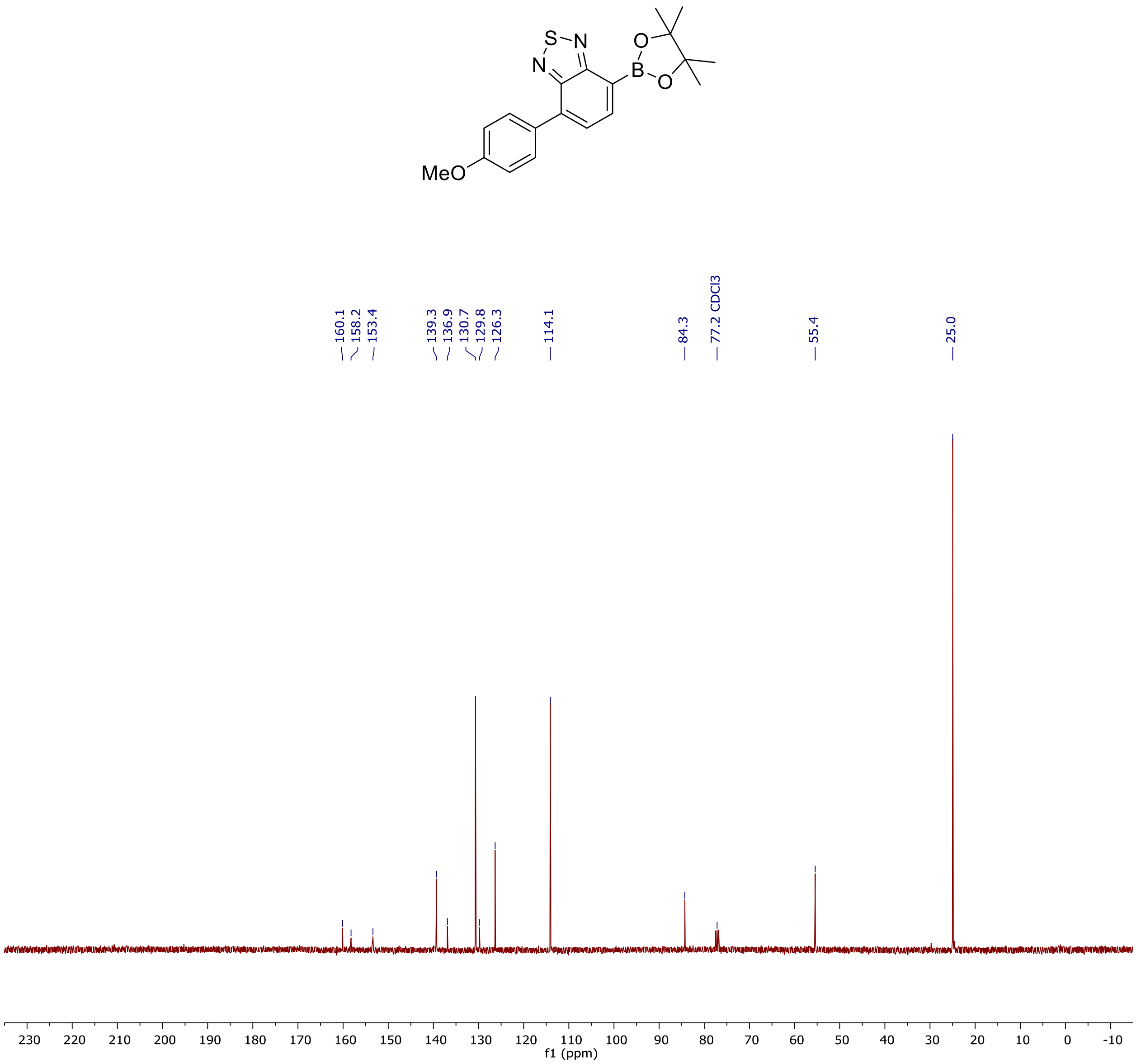

Figure S2. ^{13}C NMR (101 MHz, chloroform-*d*) spectrum of compound **2**

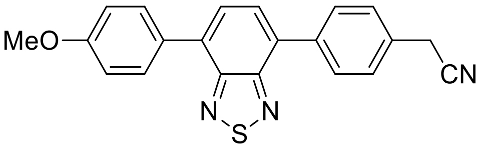

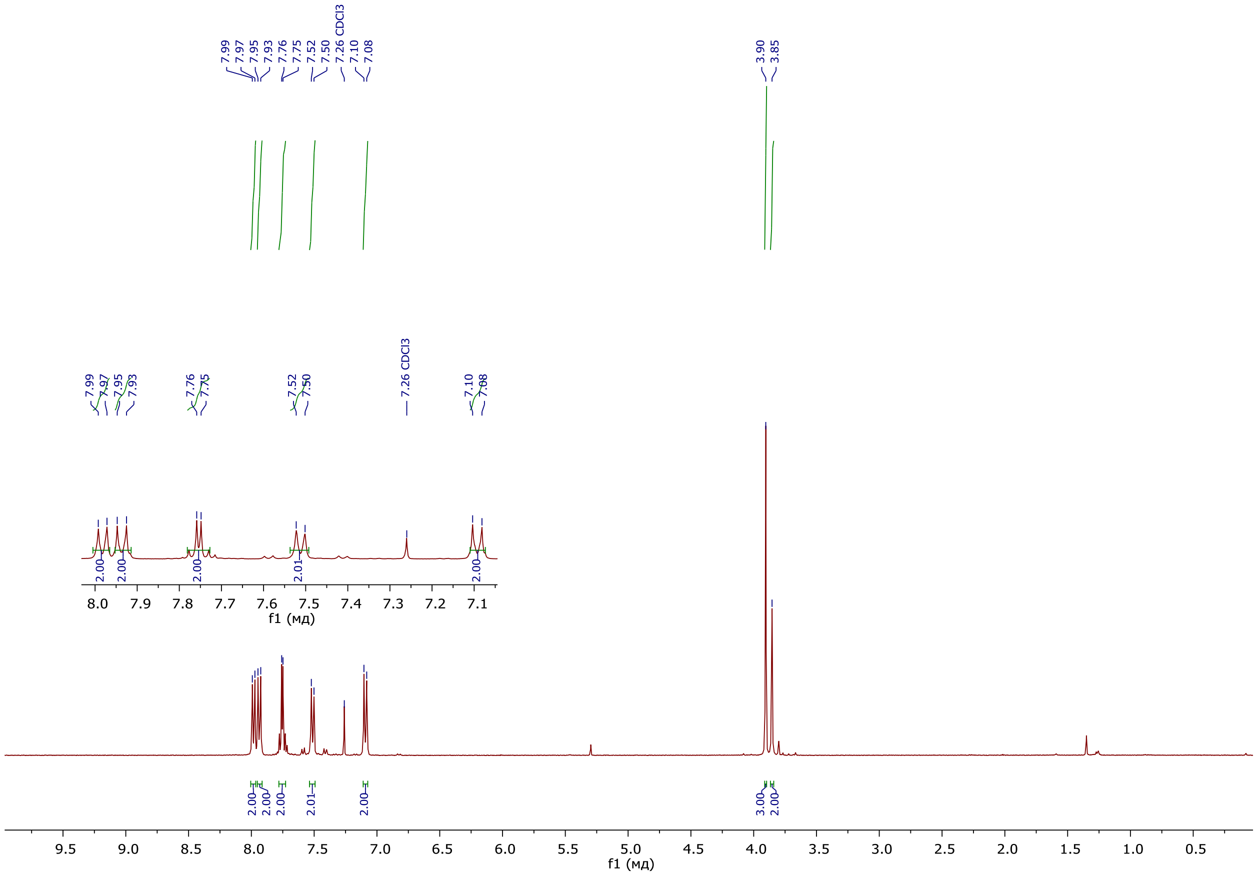

Figure S3. ^{1}H NMR (400 MHz, chloroform-*d*) spectrum of compound **3a**

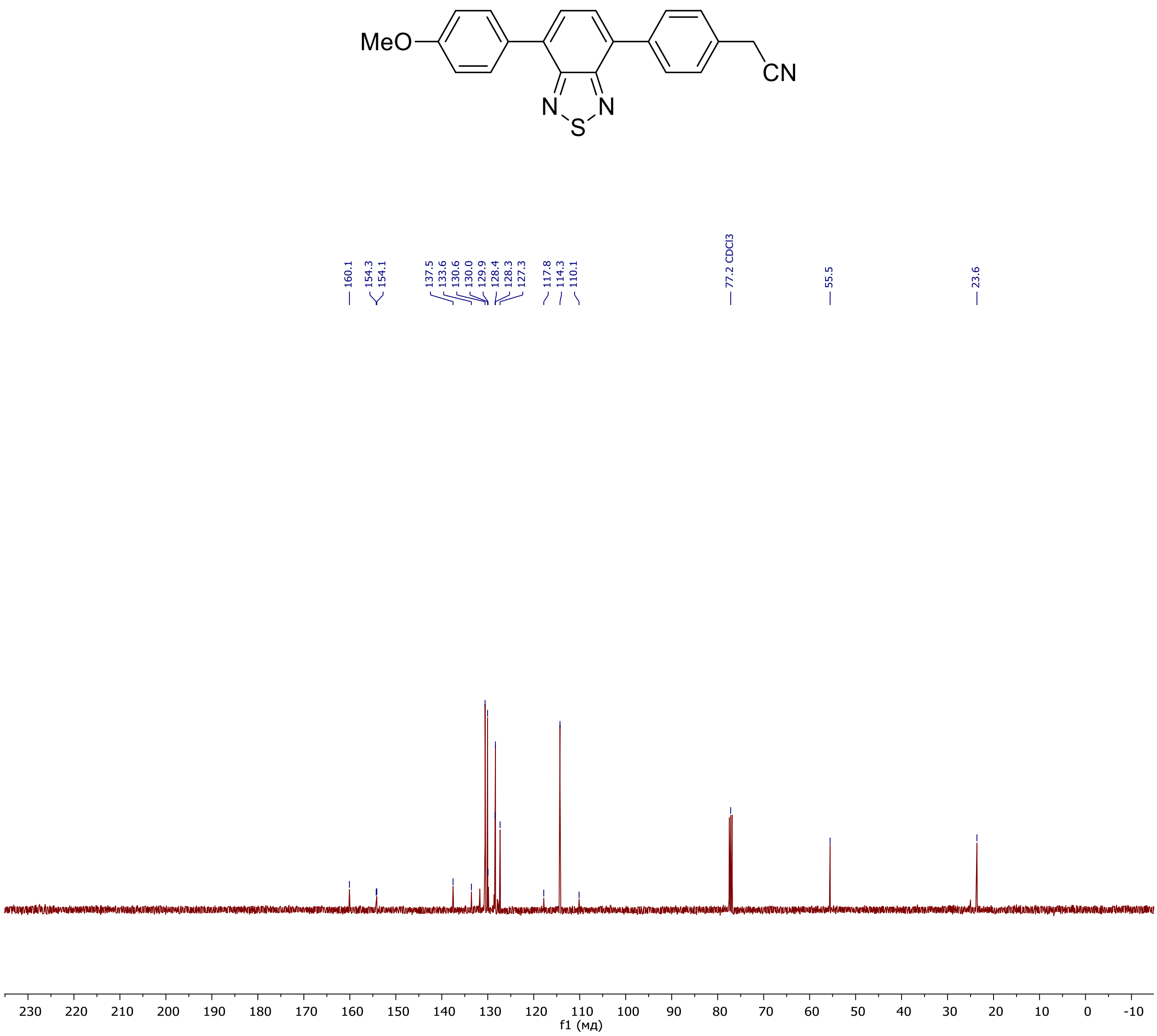

Figure S4. ^{13}C NMR (101 MHz, chloroform-*d*) spectrum of compound **3a**

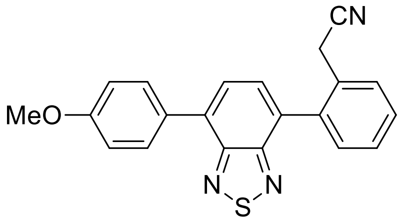

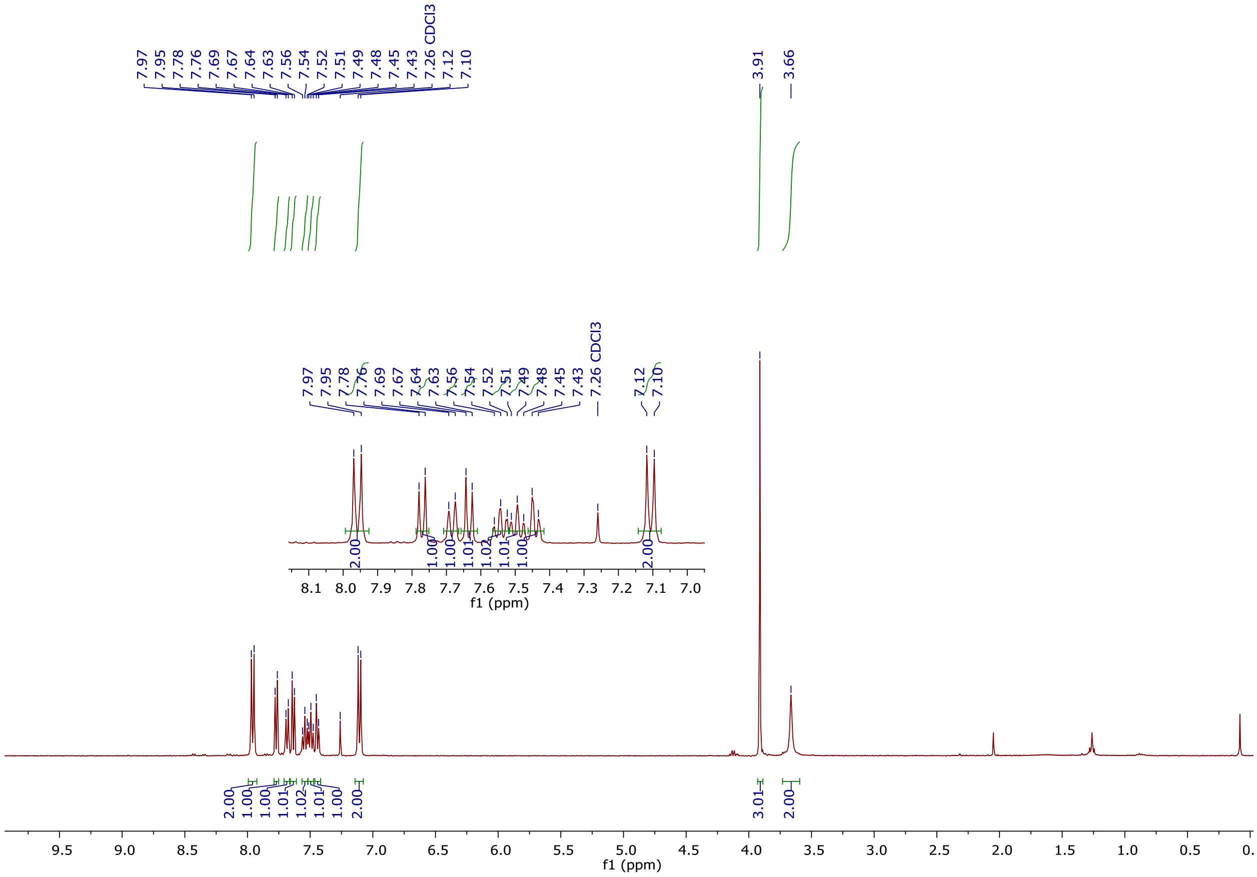

Figure S5. ^{1}H NMR (400 MHz, chloroform-*d*) spectrum of compound **3b**

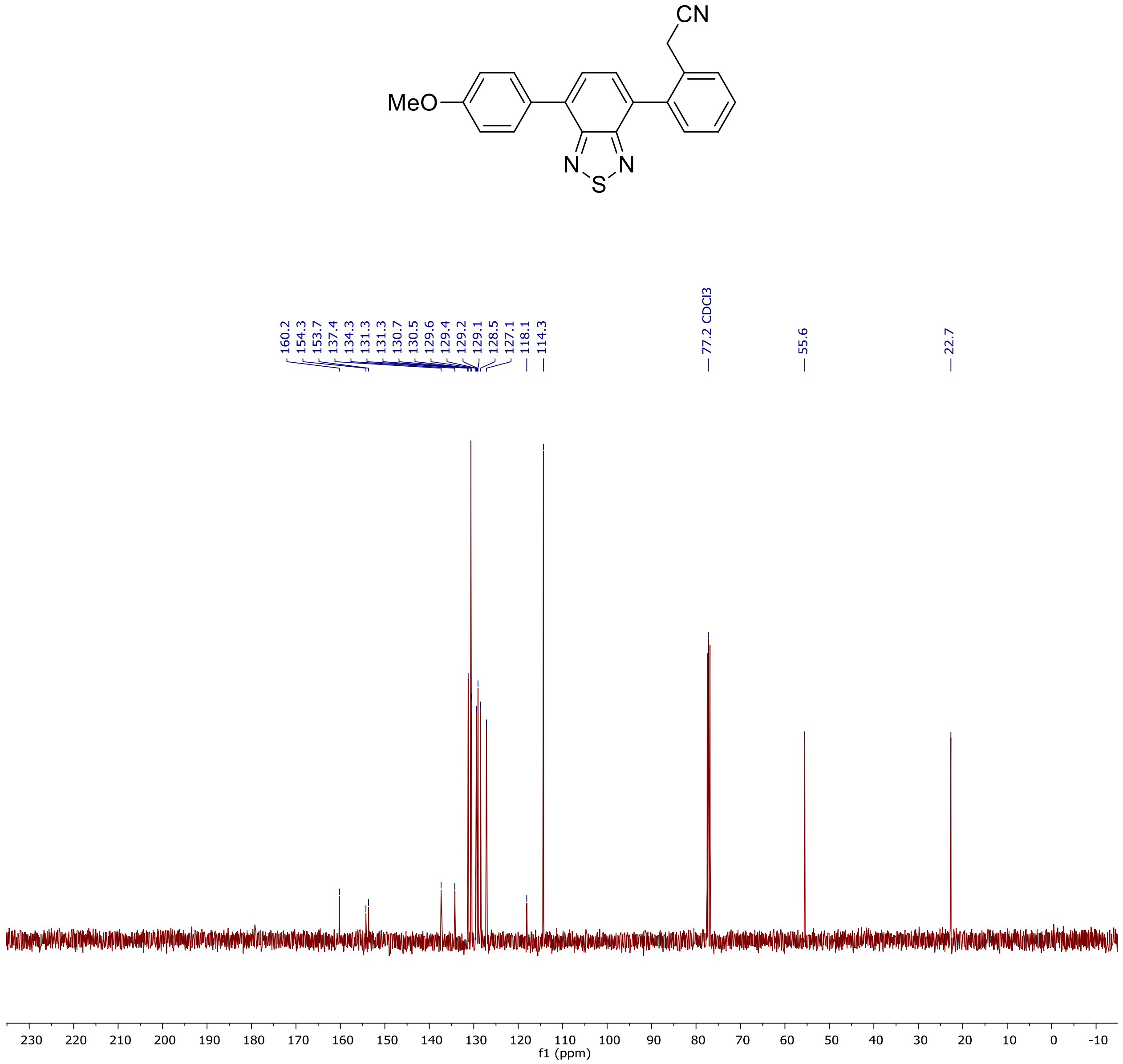

Figure S6. ^{13}C NMR (101 MHz, chloroform-*d*) spectrum of compound **3b**

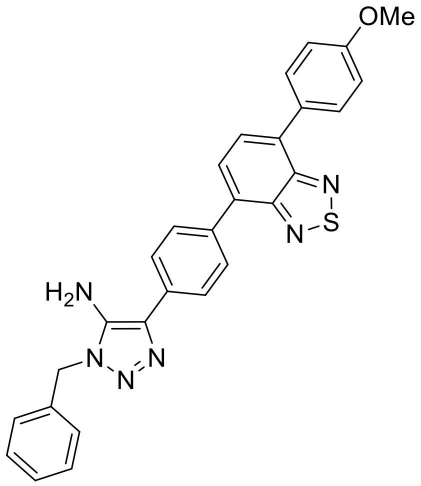

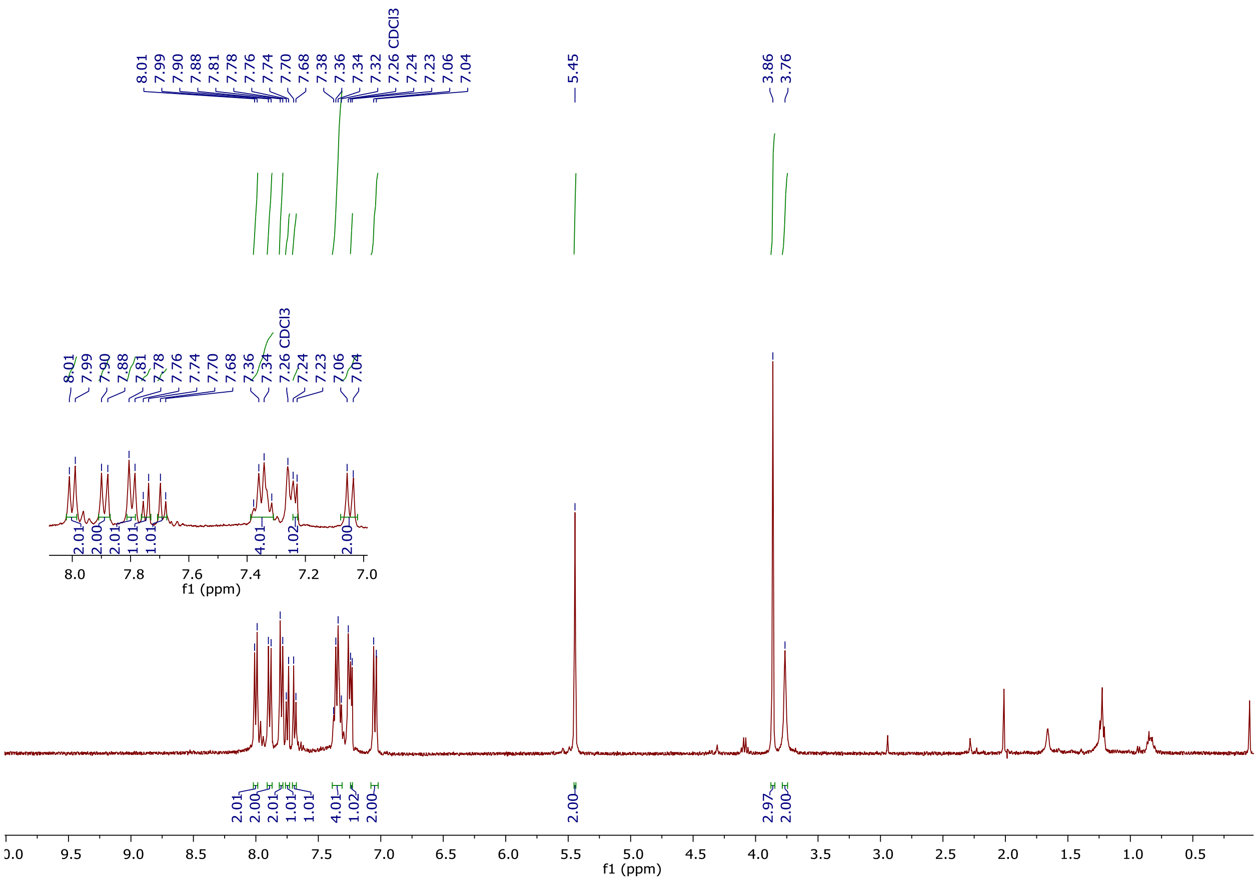

Figure S7. ^{1}H NMR (400 MHz, chloroform-*d*) spectrum of compound **4a**

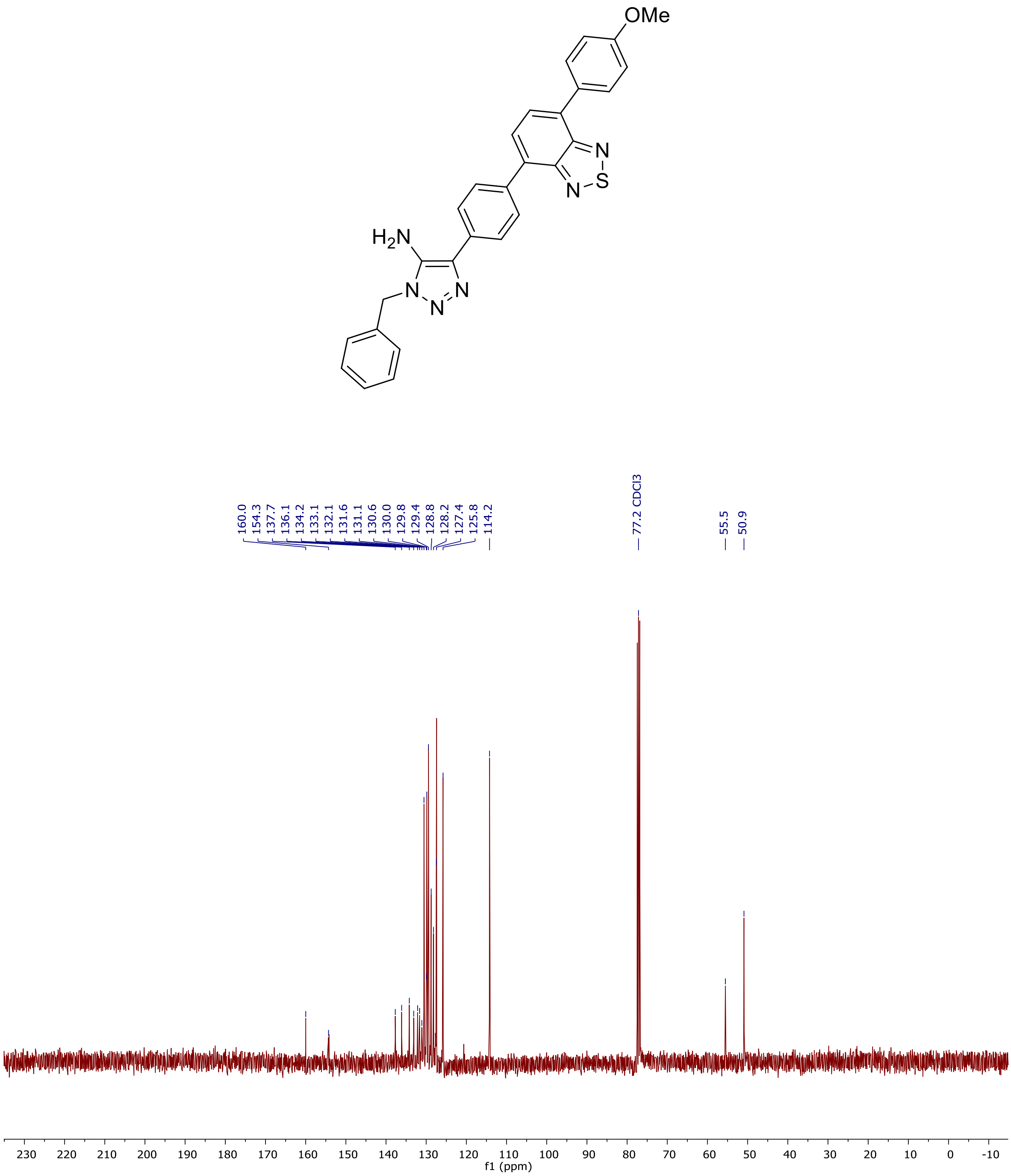

Figure S8. ^{13}C NMR (101 MHz, chloroform-*d*) spectrum of compound **4a**

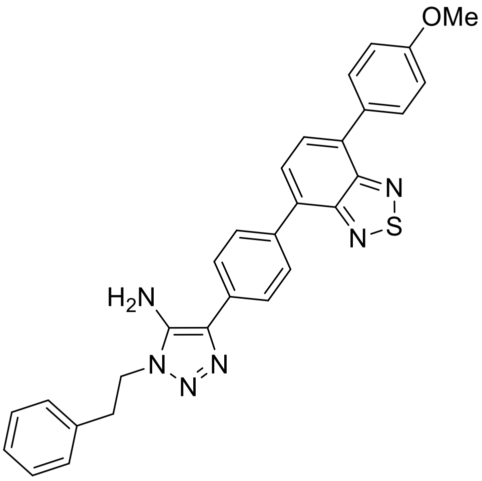

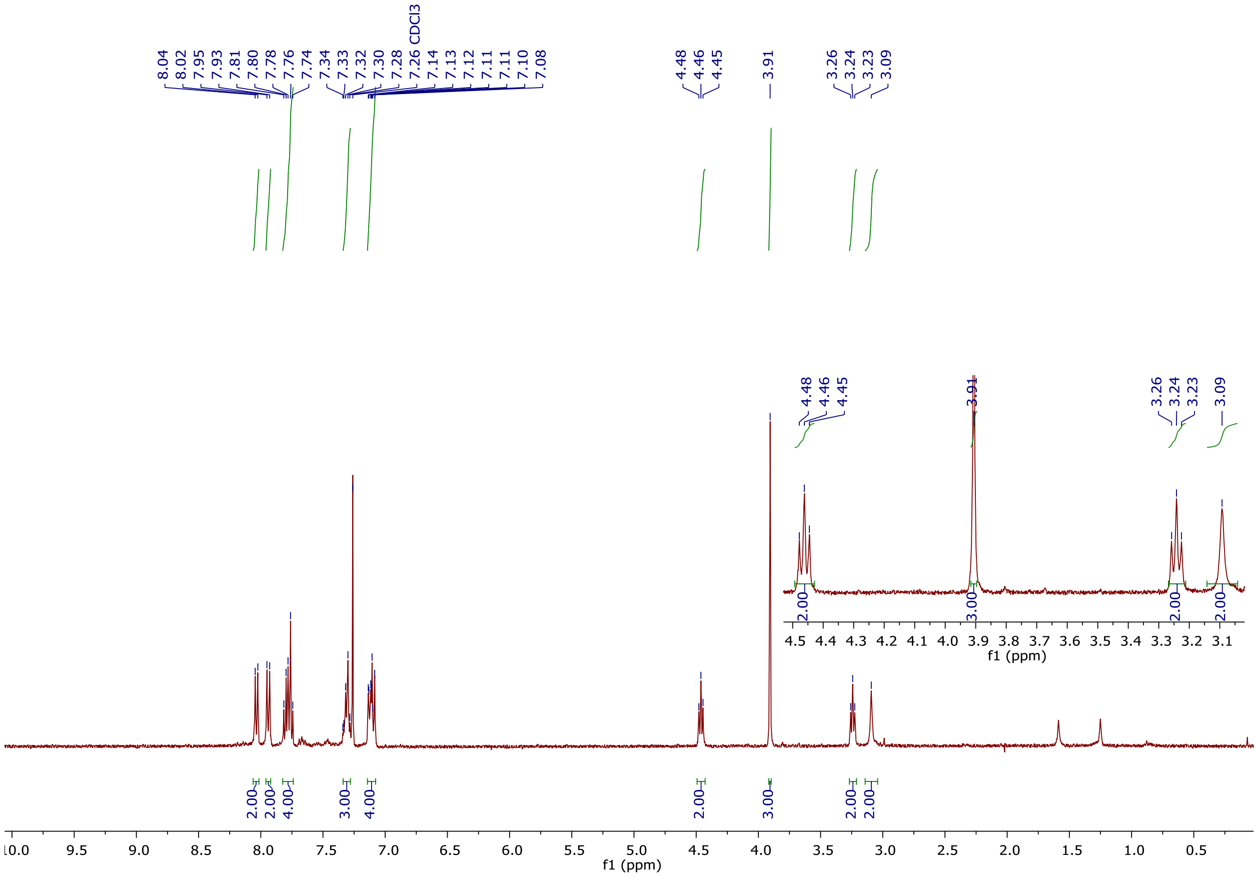

Figure S9. ^{1}H NMR (400 MHz, chloroform-*d*) spectrum of compound **4b**

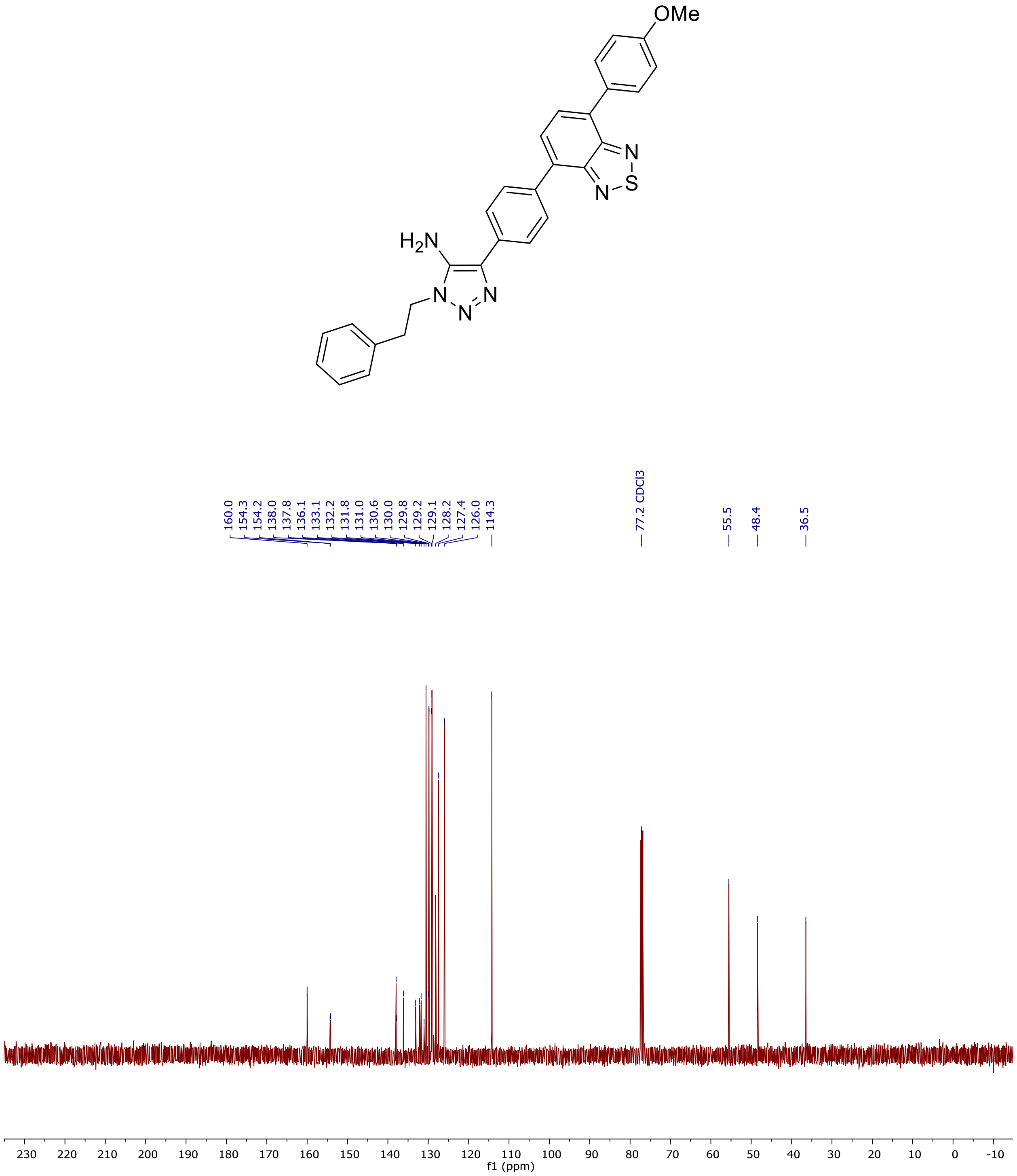

Figure S10. ^{13}C NMR (101 MHz, chloroform-*d*) spectrum of compound **4b**

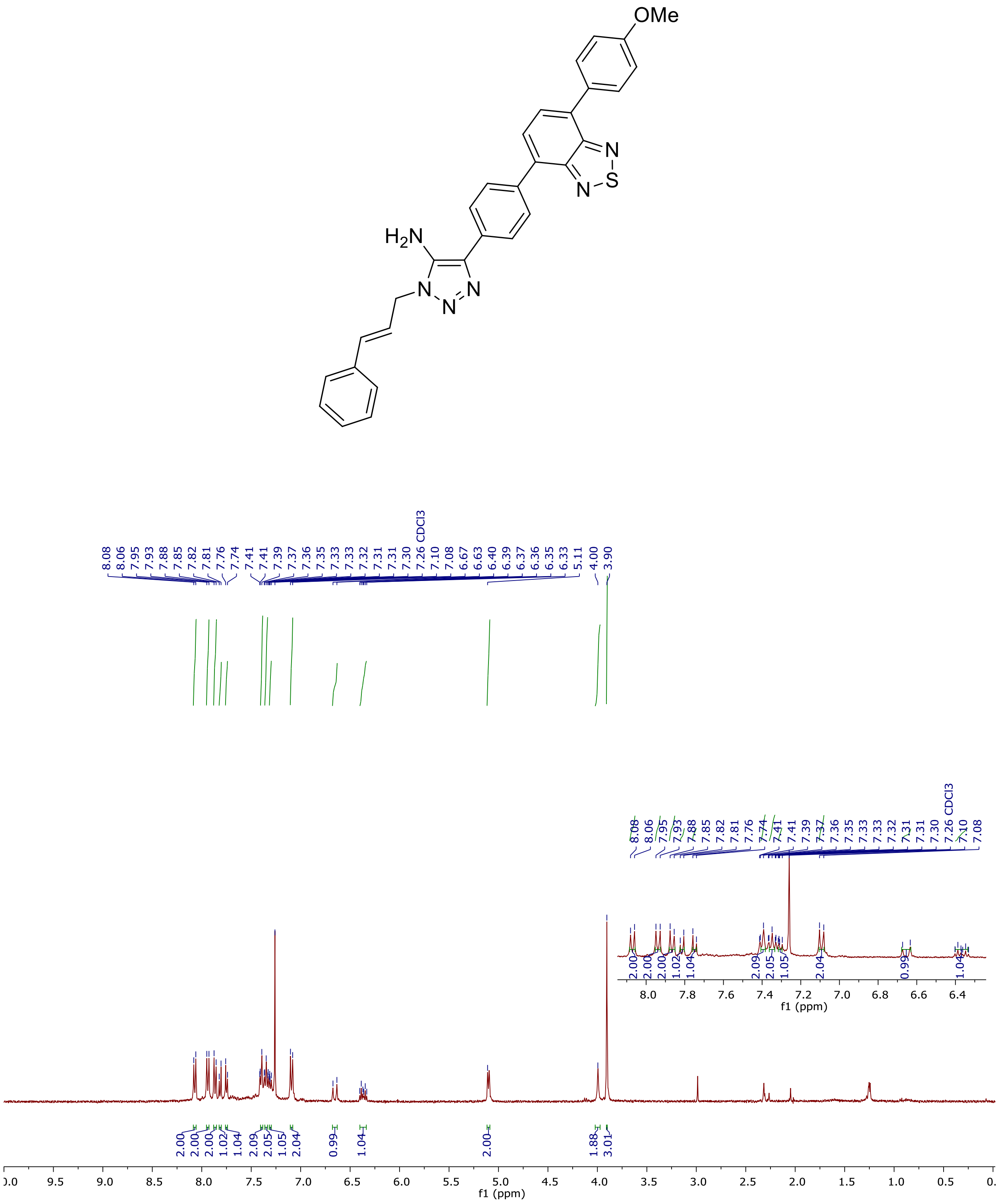

Figure S11. ^{1}H NMR (400 MHz, chloroform-*d*) spectrum of compound **4c**

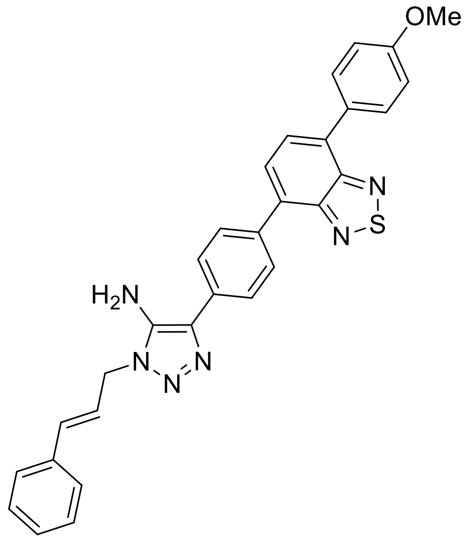

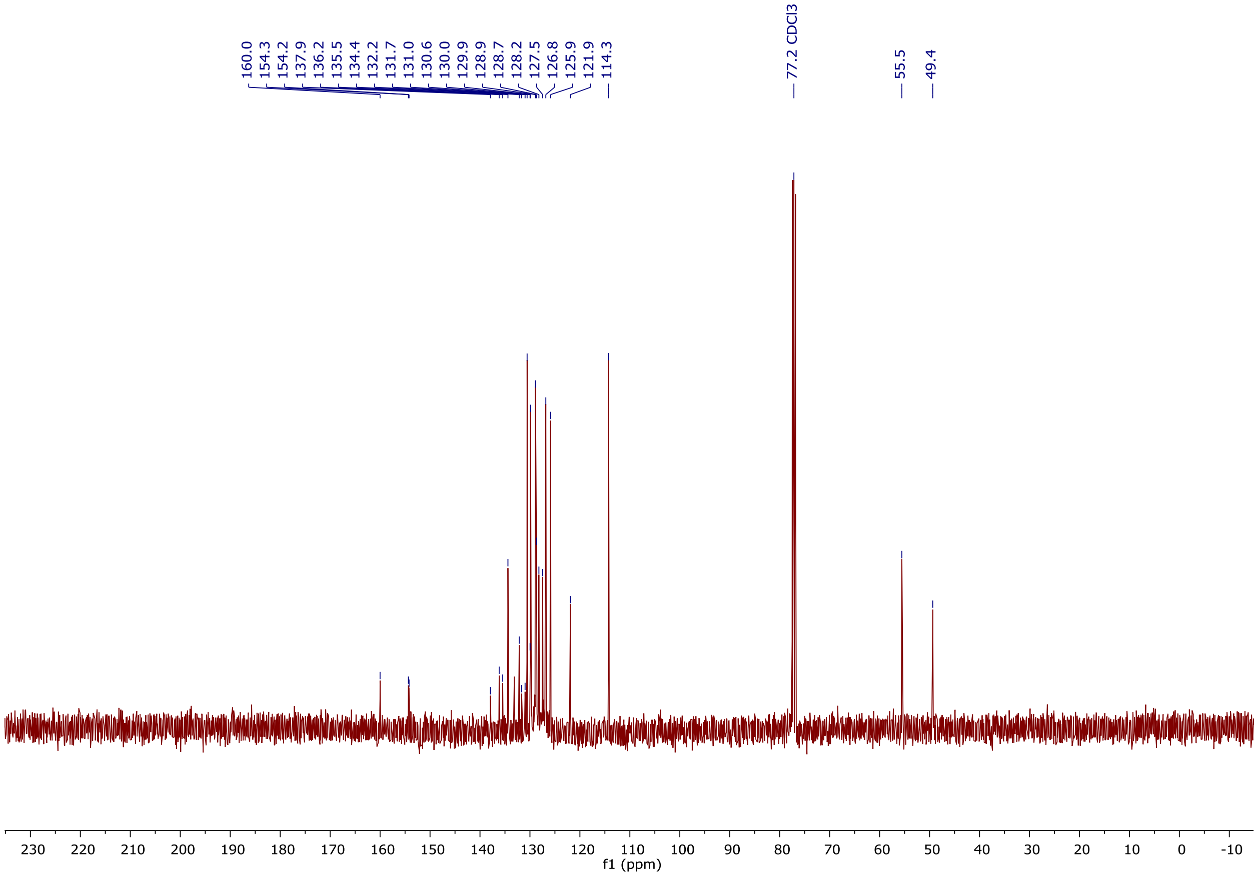

Figure S12. ^{13}C NMR (101 MHz, chloroform-*d*) spectrum of compound **4c**

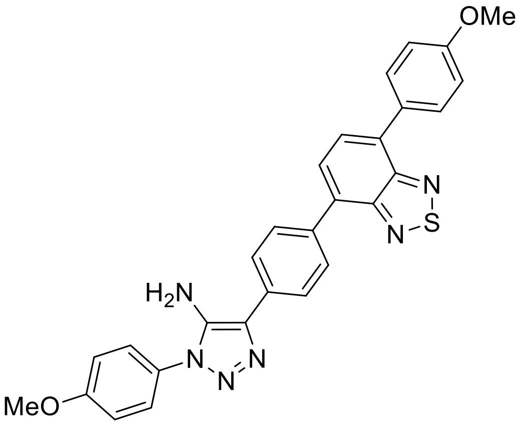

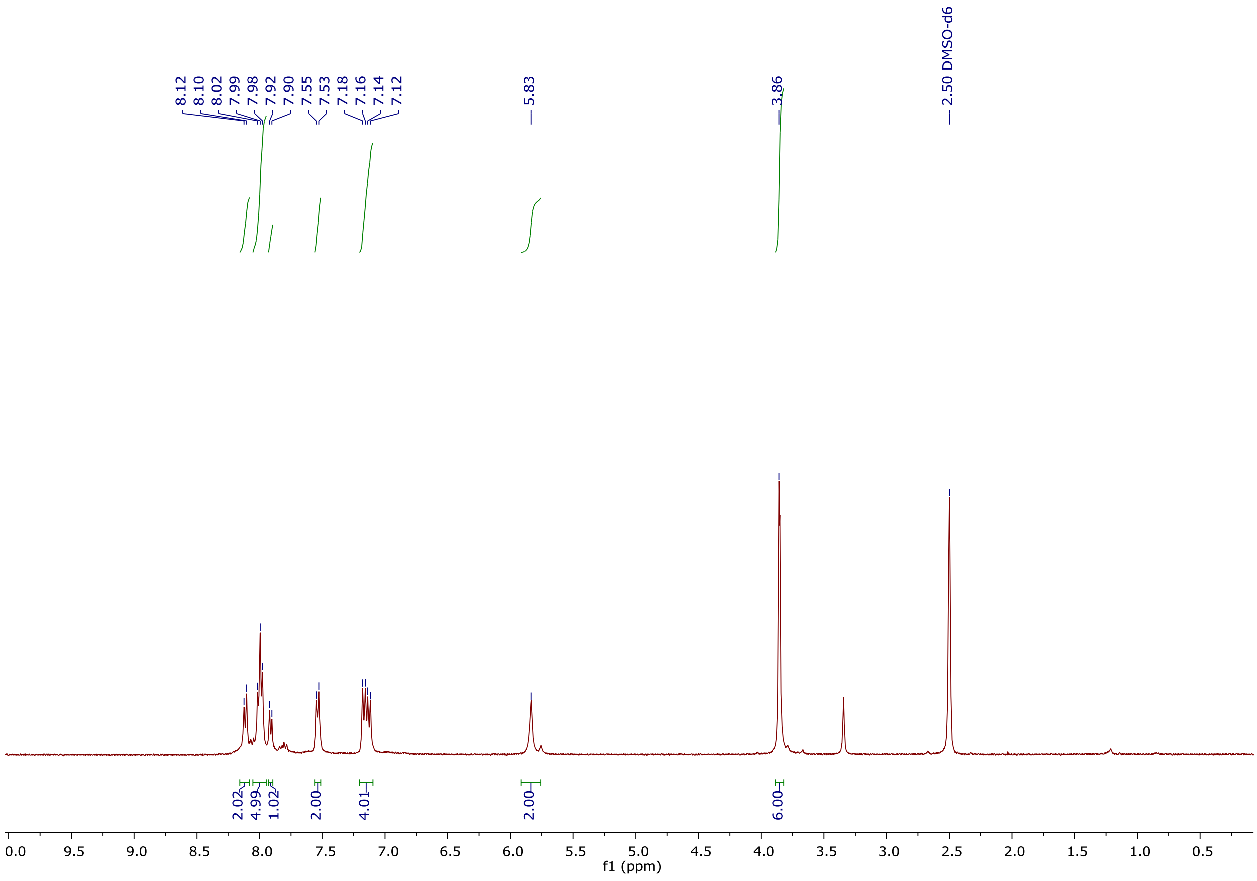

Figure S13. ^{1}H NMR (400 MHz, DMSO-d_6) spectrum of compound **4d**

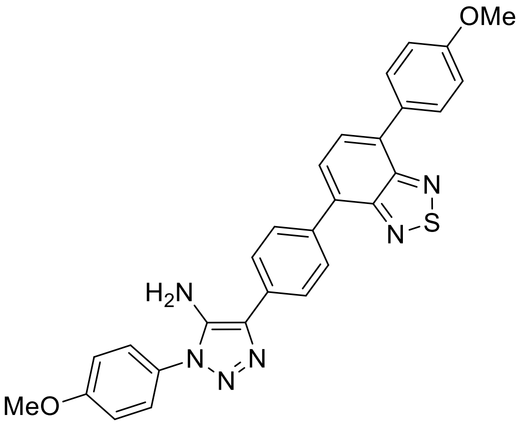

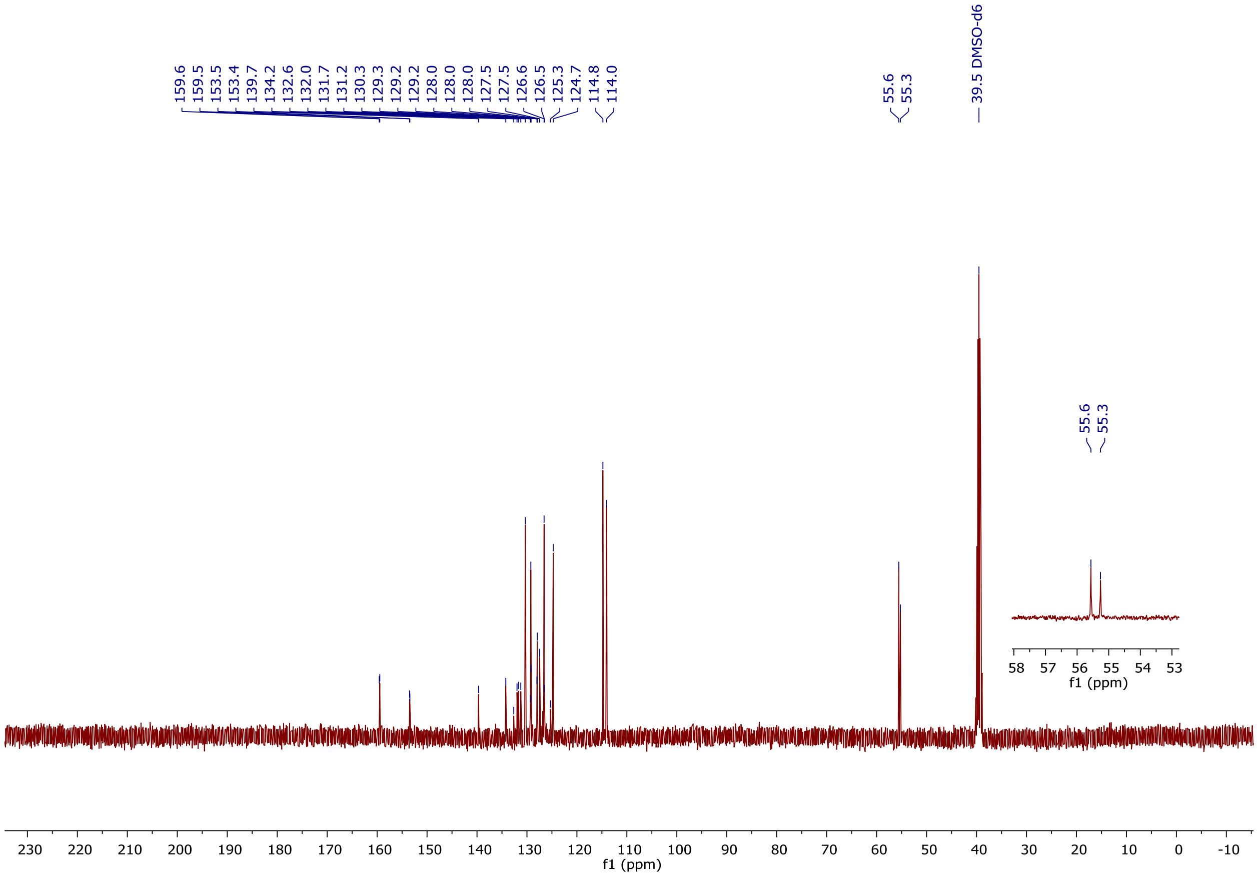

Figure S14. ^{13}C NMR (101 MHz, DMSO-d_6) spectrum of compound **4d**

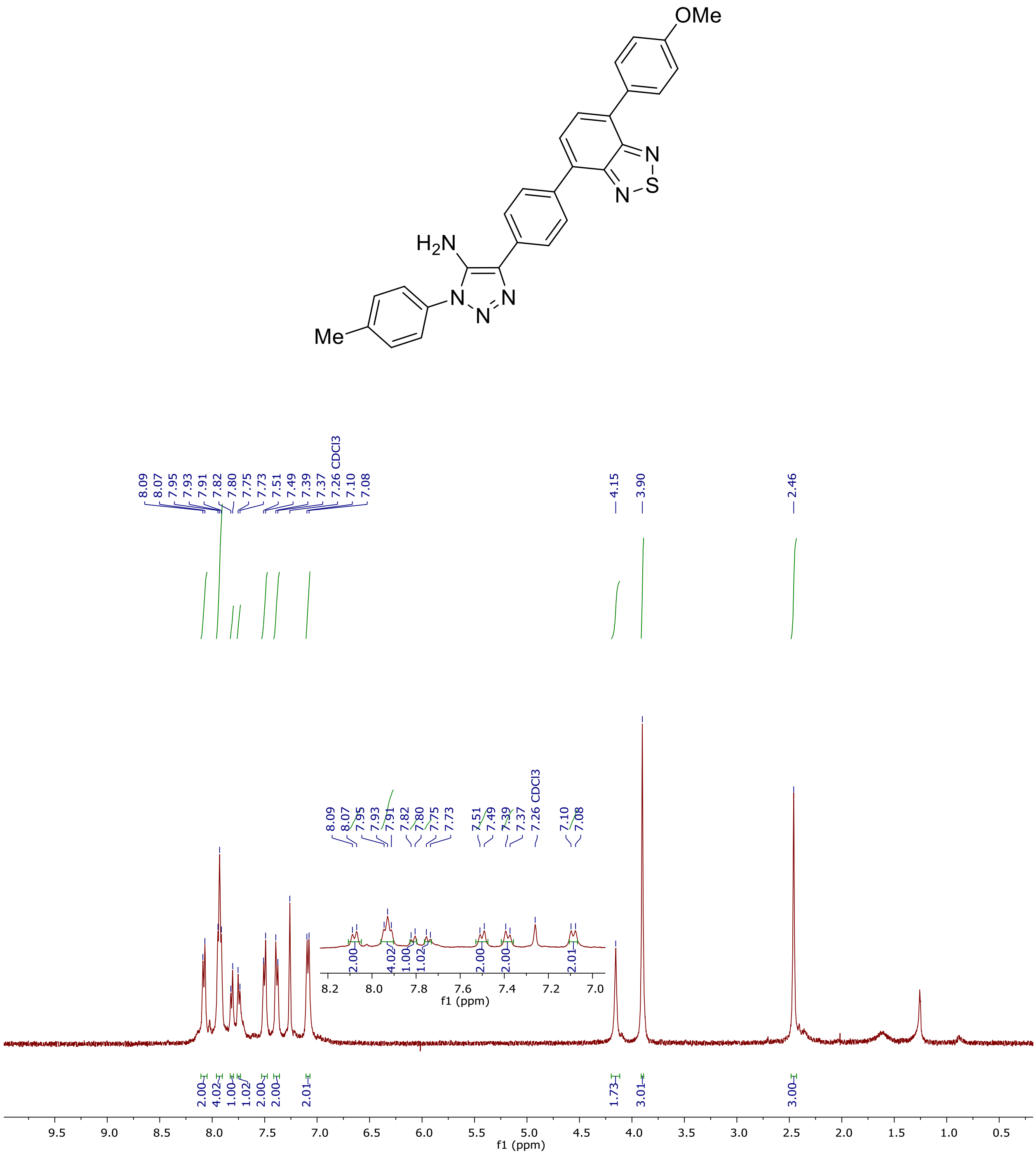

Figure S15. ^{1}H NMR (400 MHz, chloroform-*d*) spectrum of compound **4e**

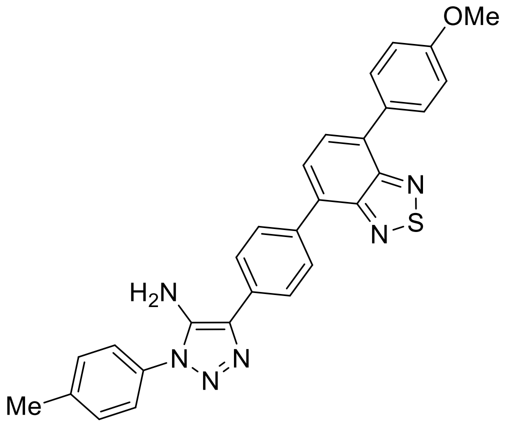

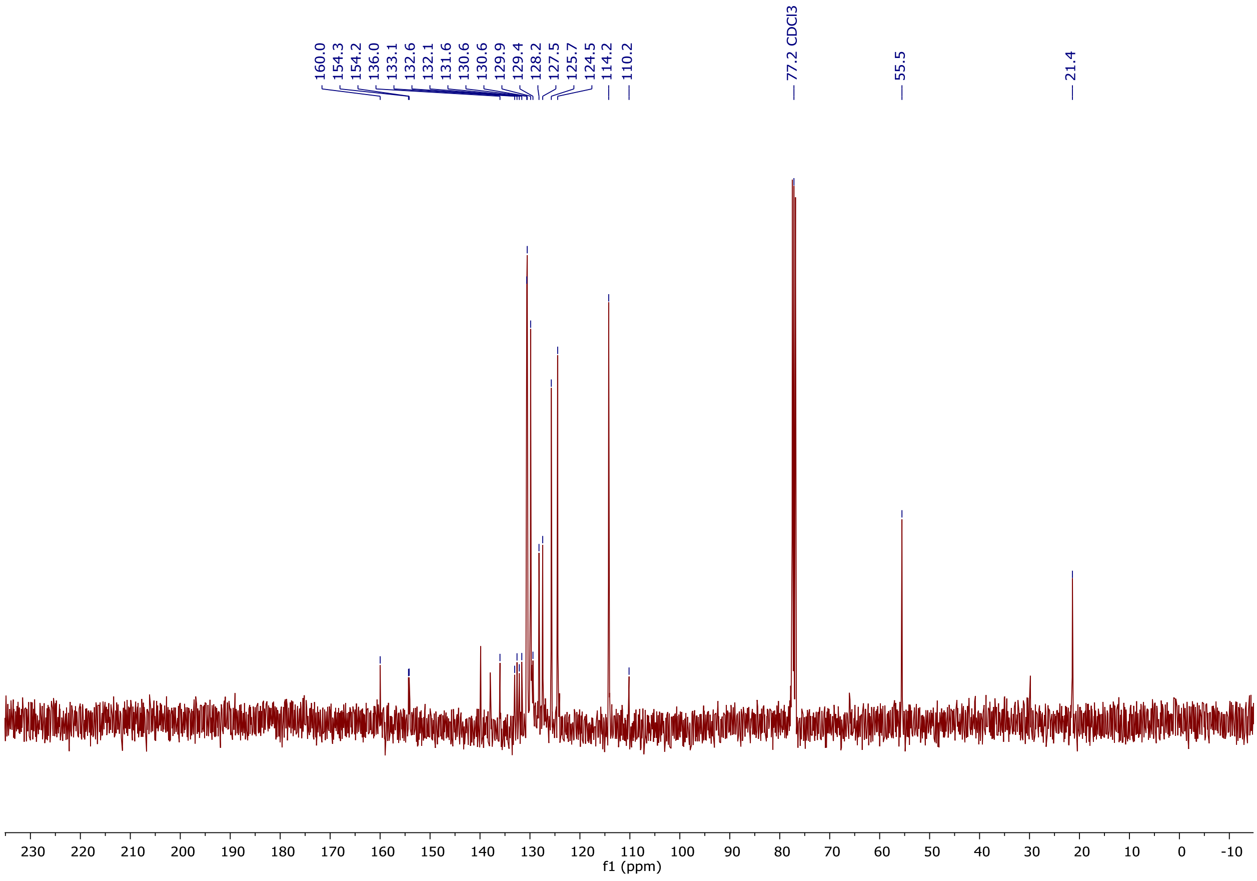

Figure S16. ^{13}C NMR (101 MHz, chloroform-*d*) spectrum of compound **4e**

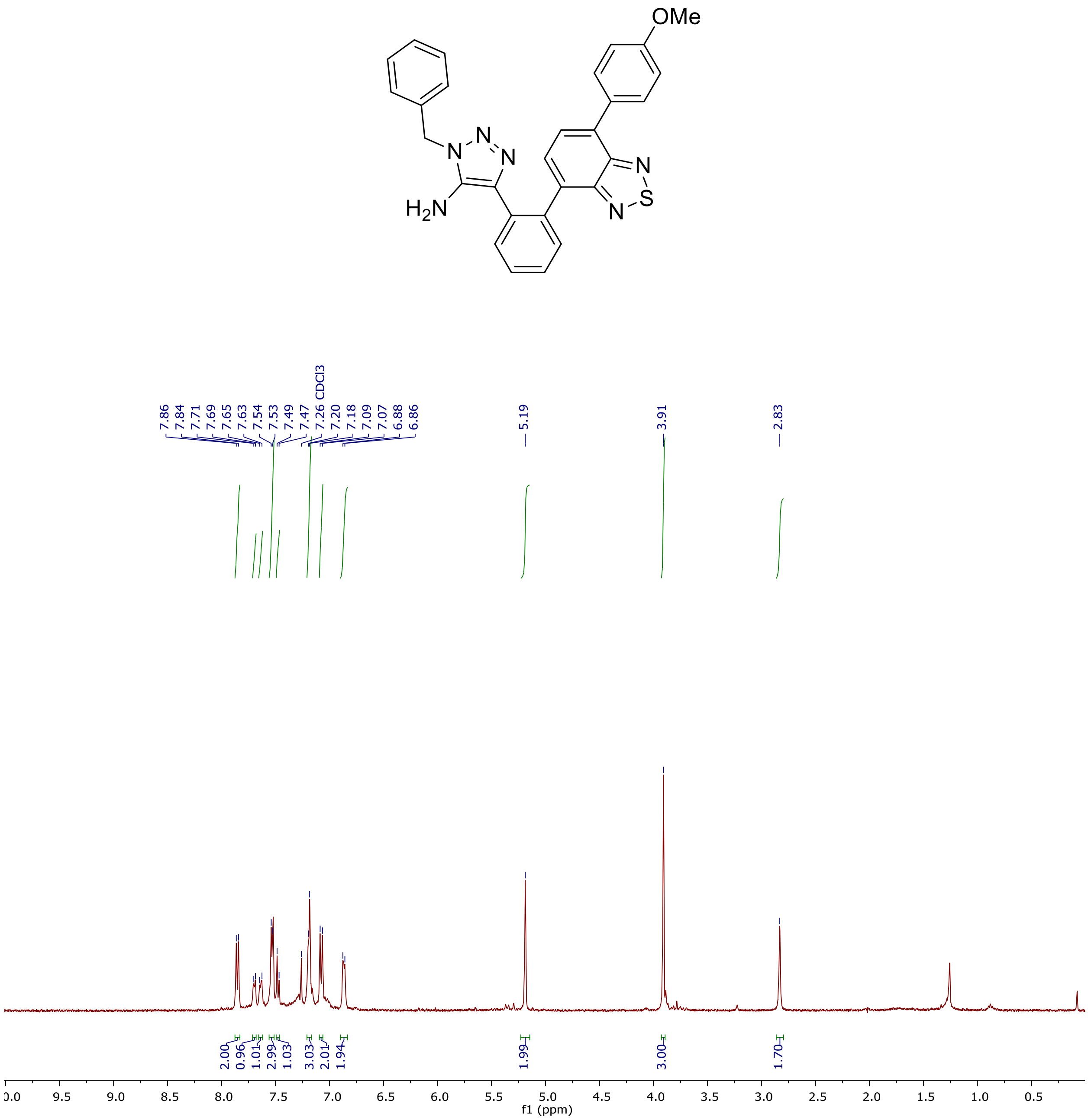

Figure S17. ^{1}H NMR (400 MHz, chloroform-*d*) spectrum of compound **4f**

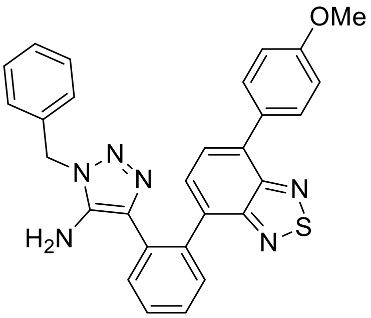

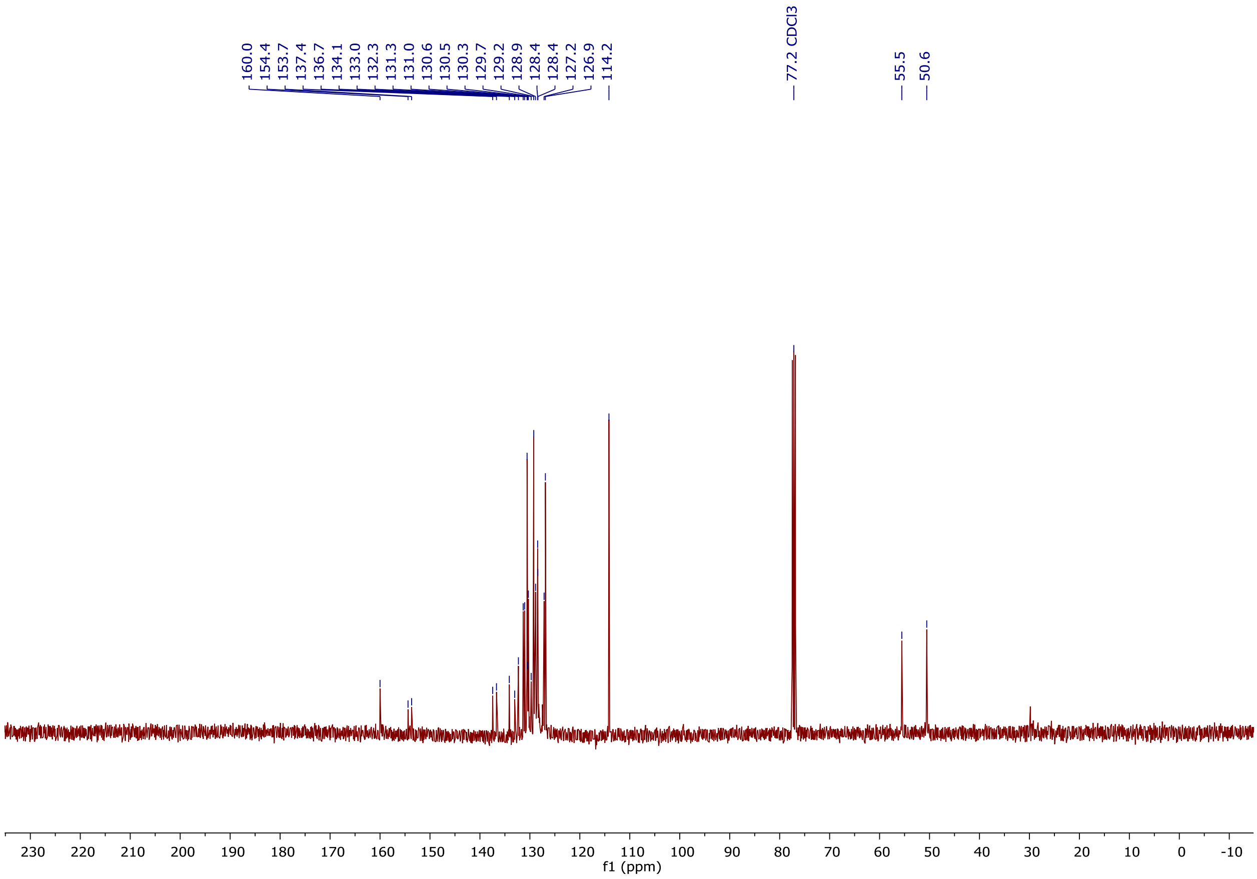

Figure S18. ^{13}C NMR (101 MHz, chloroform-*d*) spectrum of compound **4f**

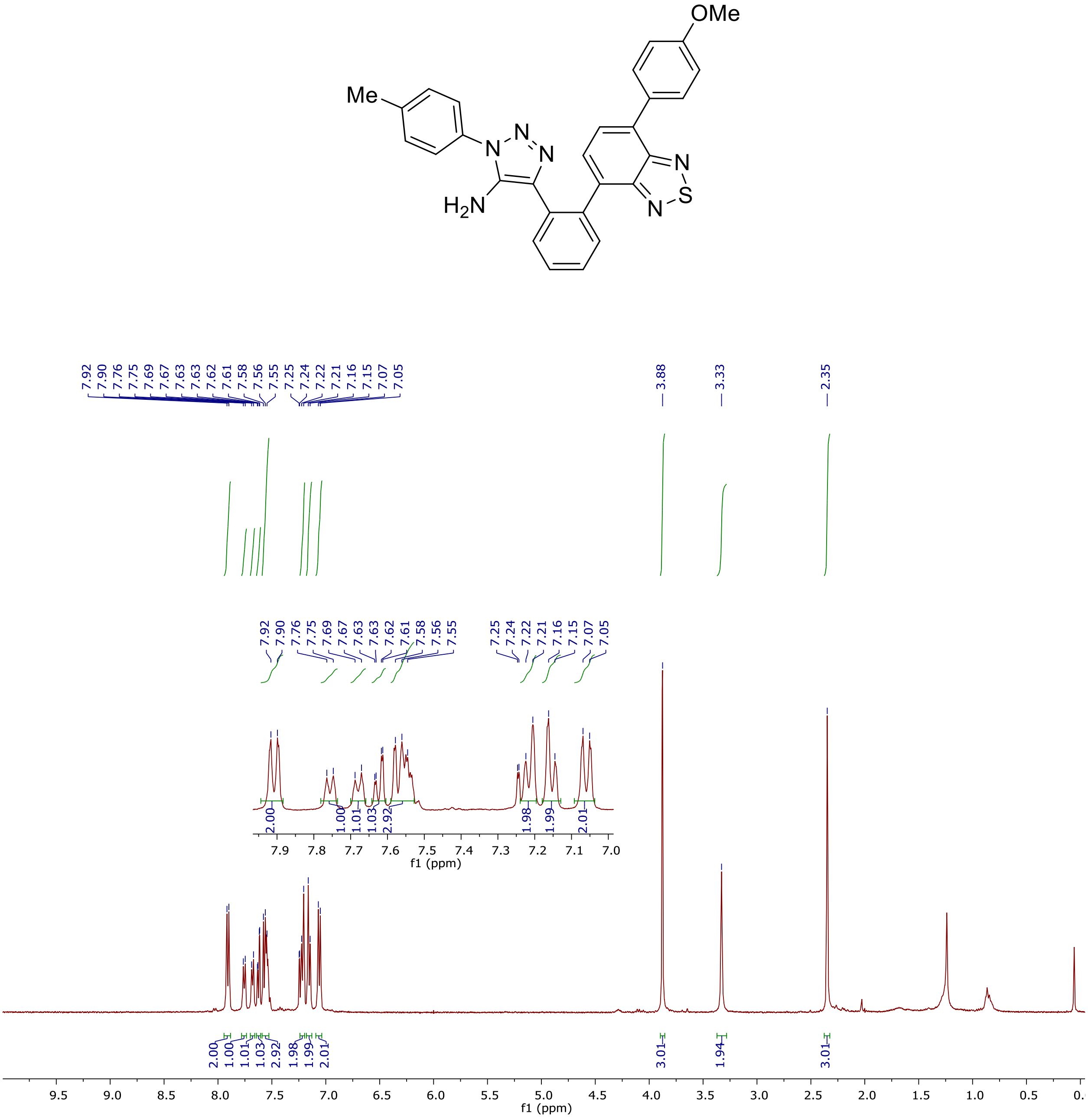

Figure S19. ^{1}H NMR (400 MHz, chloroform-*d*) spectrum of compound **4g**

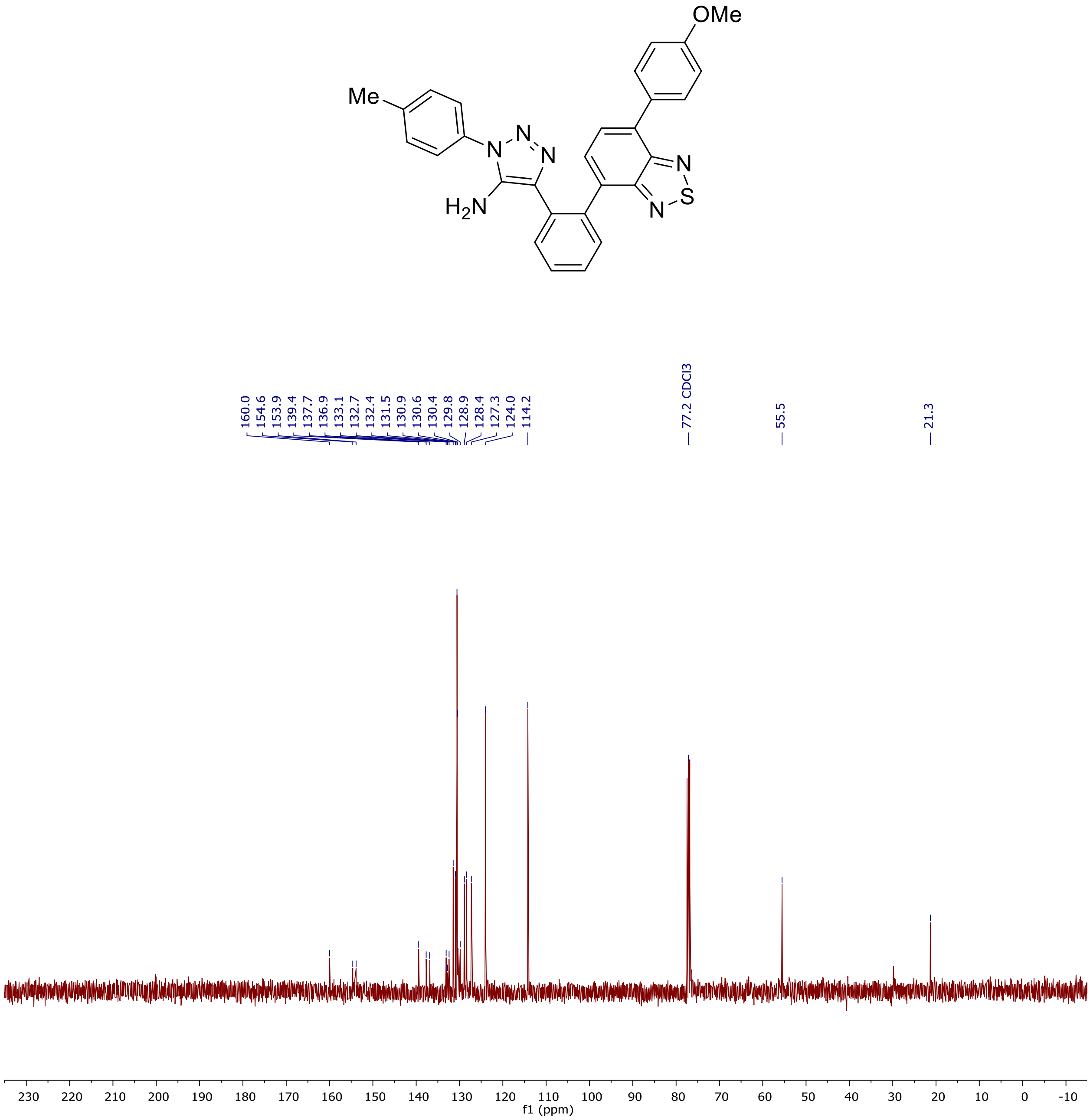

Figure S20. ^{13}C NMR (101 MHz, chloroform-*d*) spectrum of compound **4g**

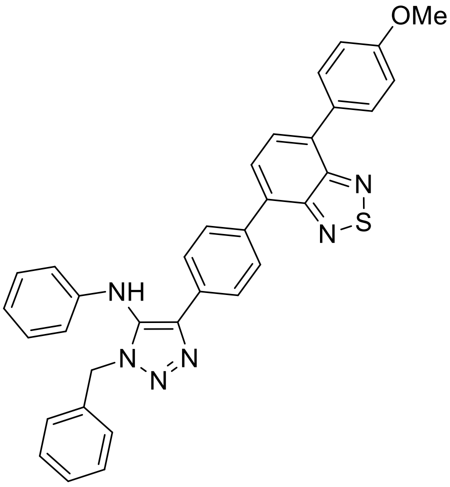

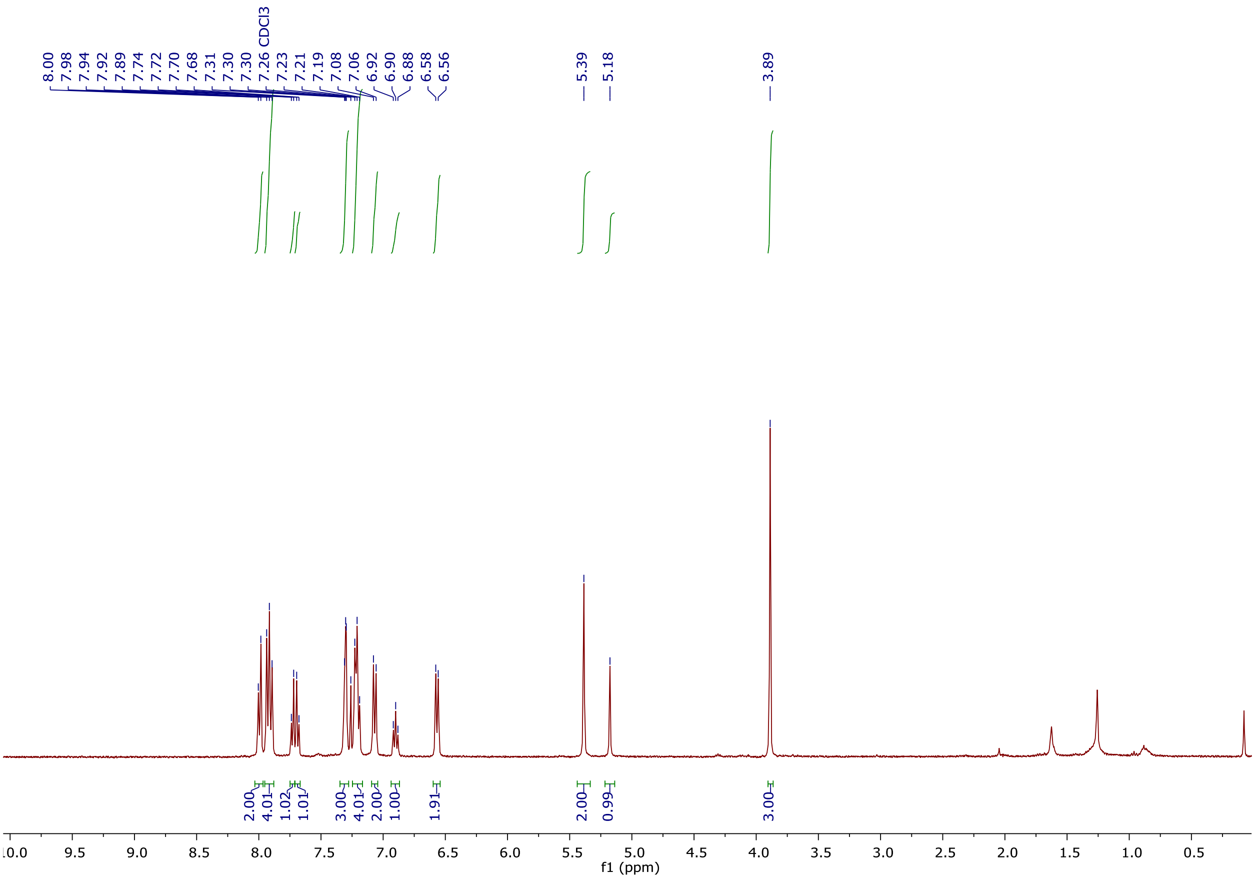

Figure S21. ^{1}H NMR (400 MHz, chloroform-*d*) spectrum of compound **5a**

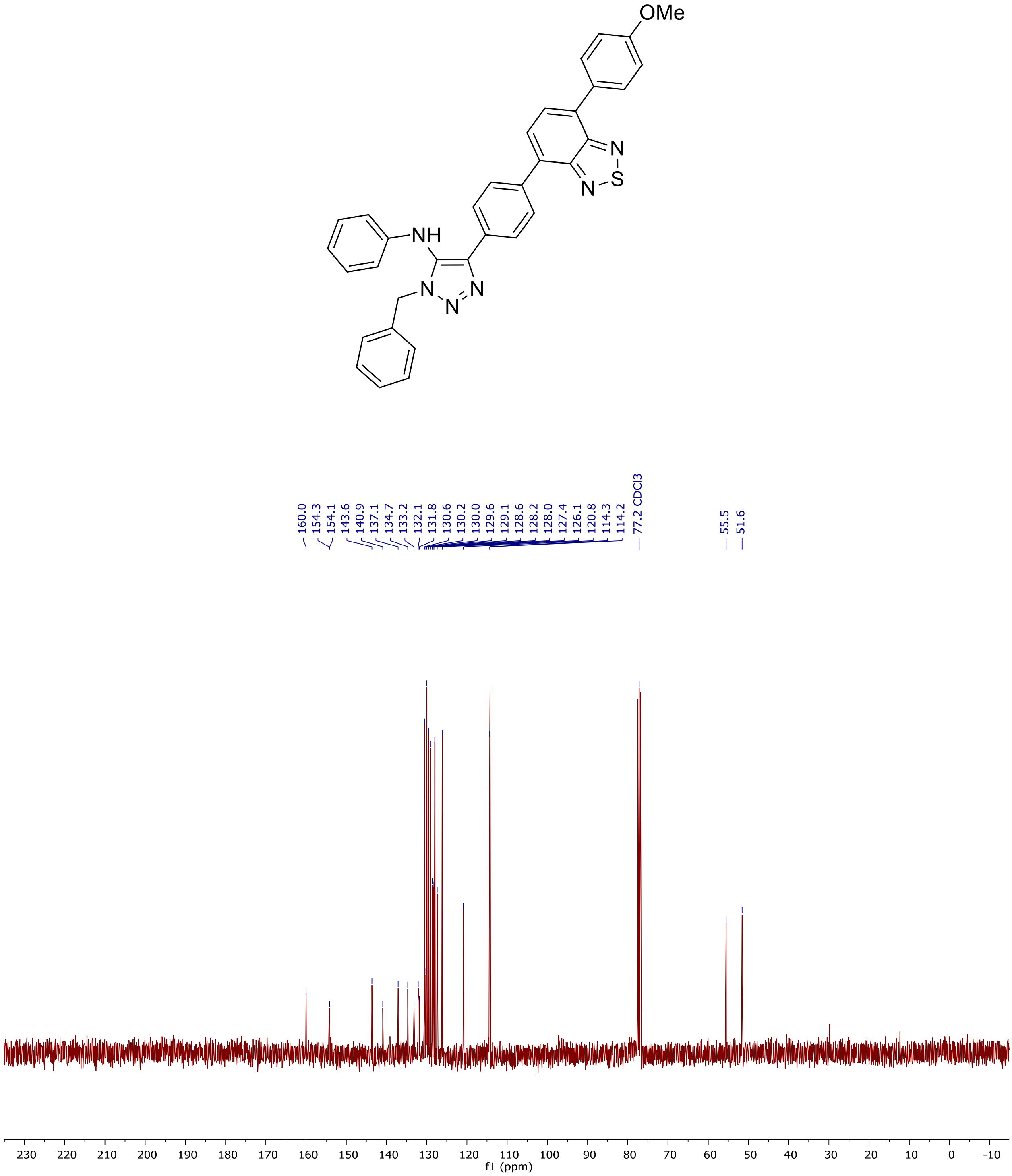

Figure S22. ^{13}C NMR (101 MHz, chloroform-*d*) spectrum of compound **5a**

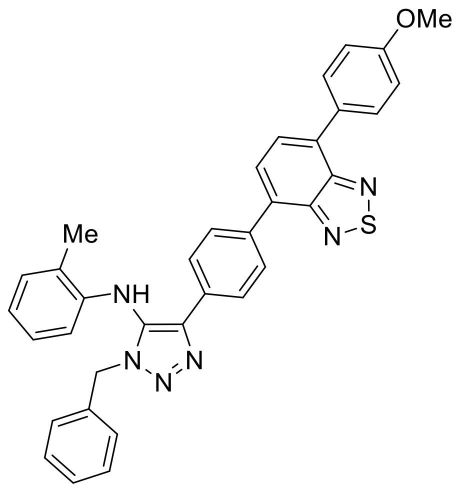

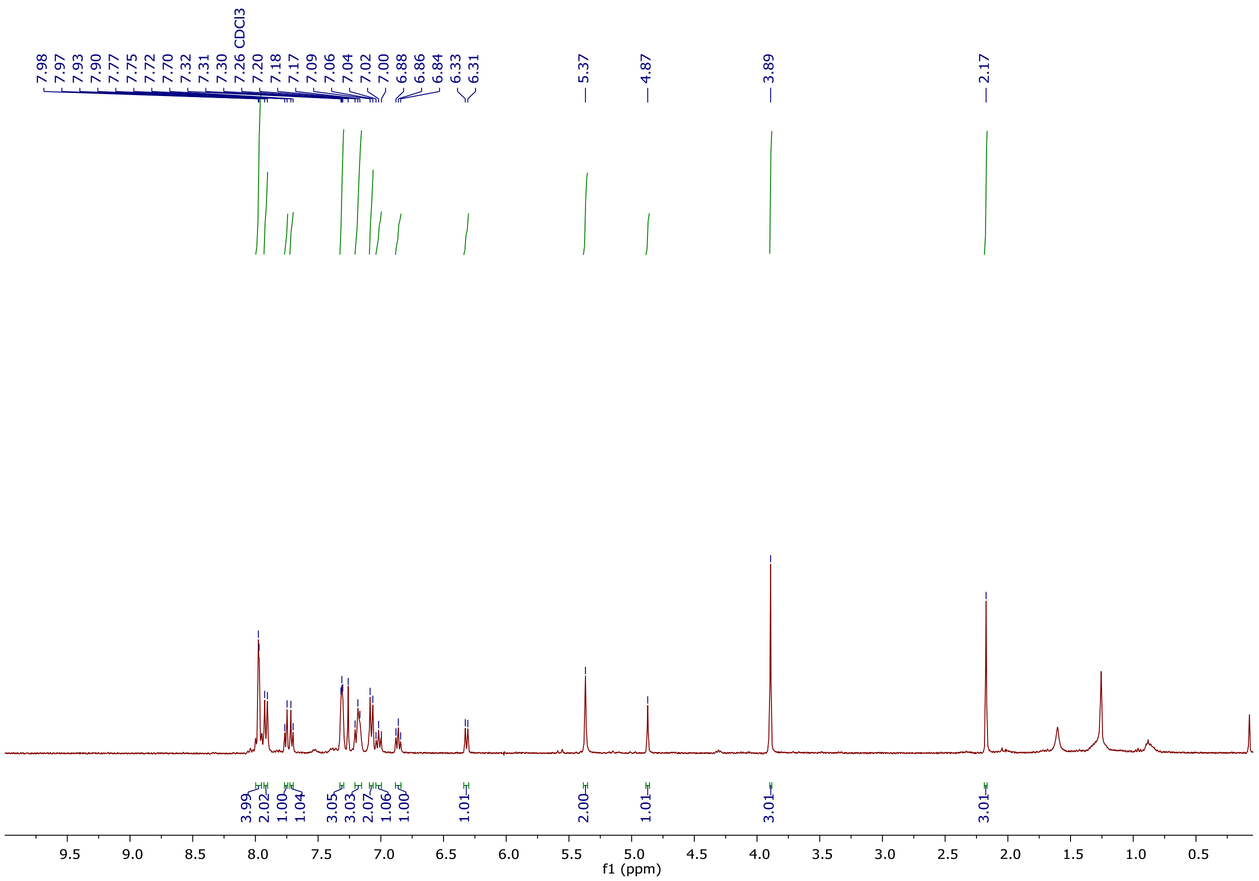

Figure S23. ^{1}H NMR (400 MHz, chloroform-*d*) spectrum of compound **5b**

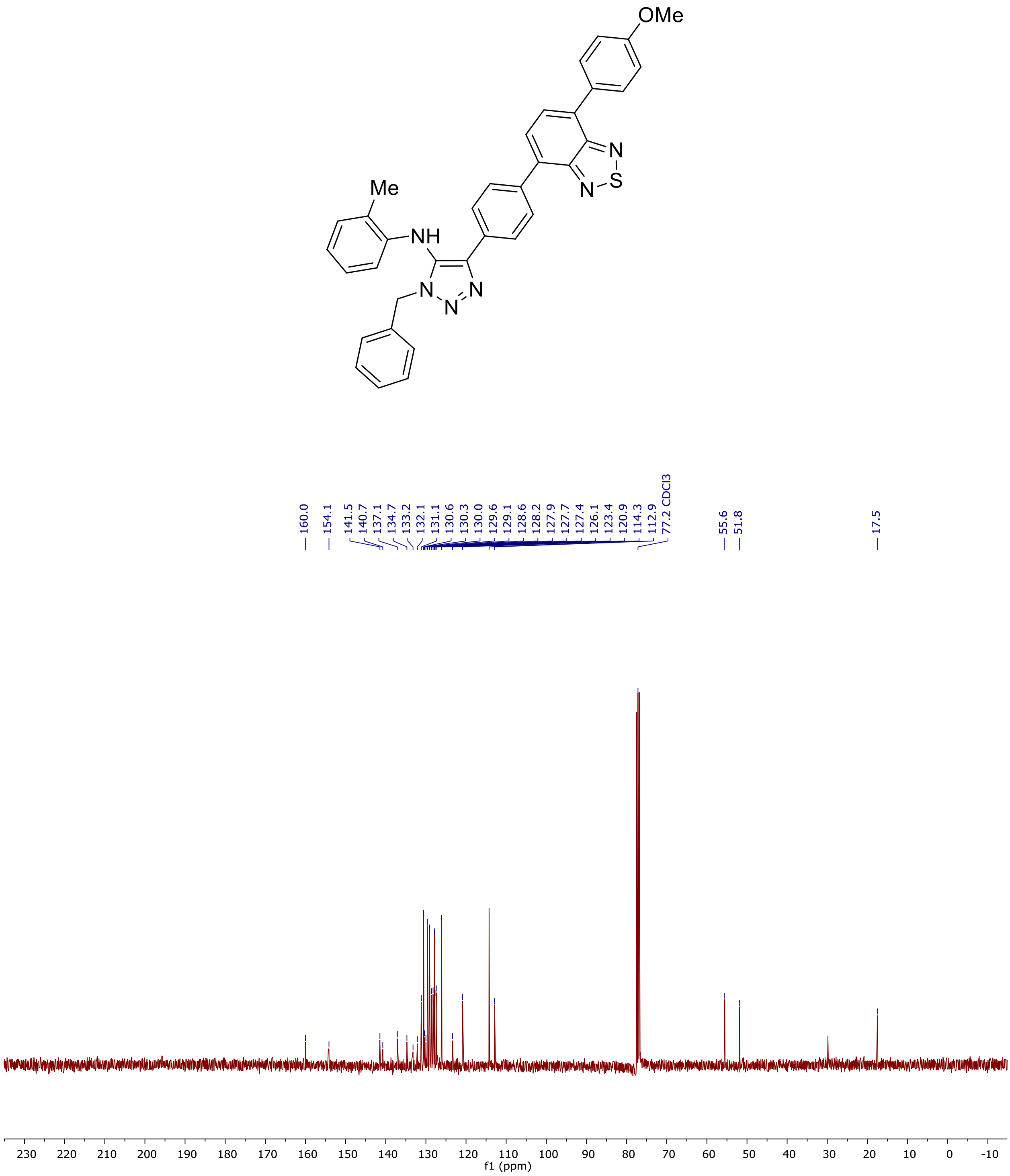

Figure S24. ^{13}C NMR (101 MHz, chloroform-*d*) spectrum of compound **5b**

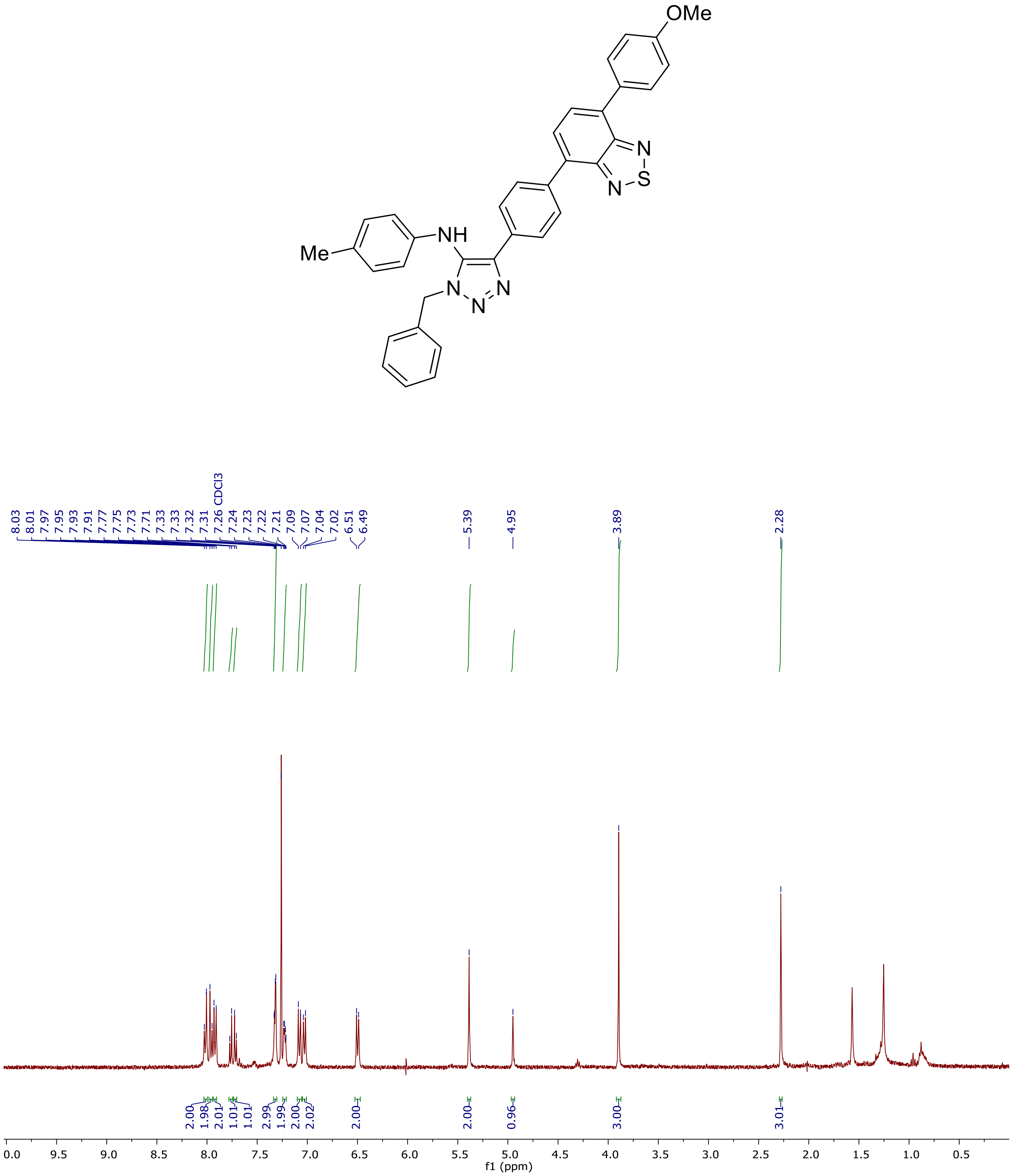

Figure S25. ^{1}H NMR (400 MHz, chloroform-*d*) spectrum of compound **5c**

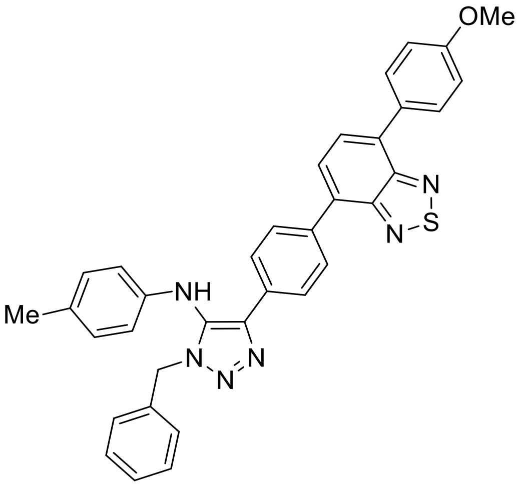

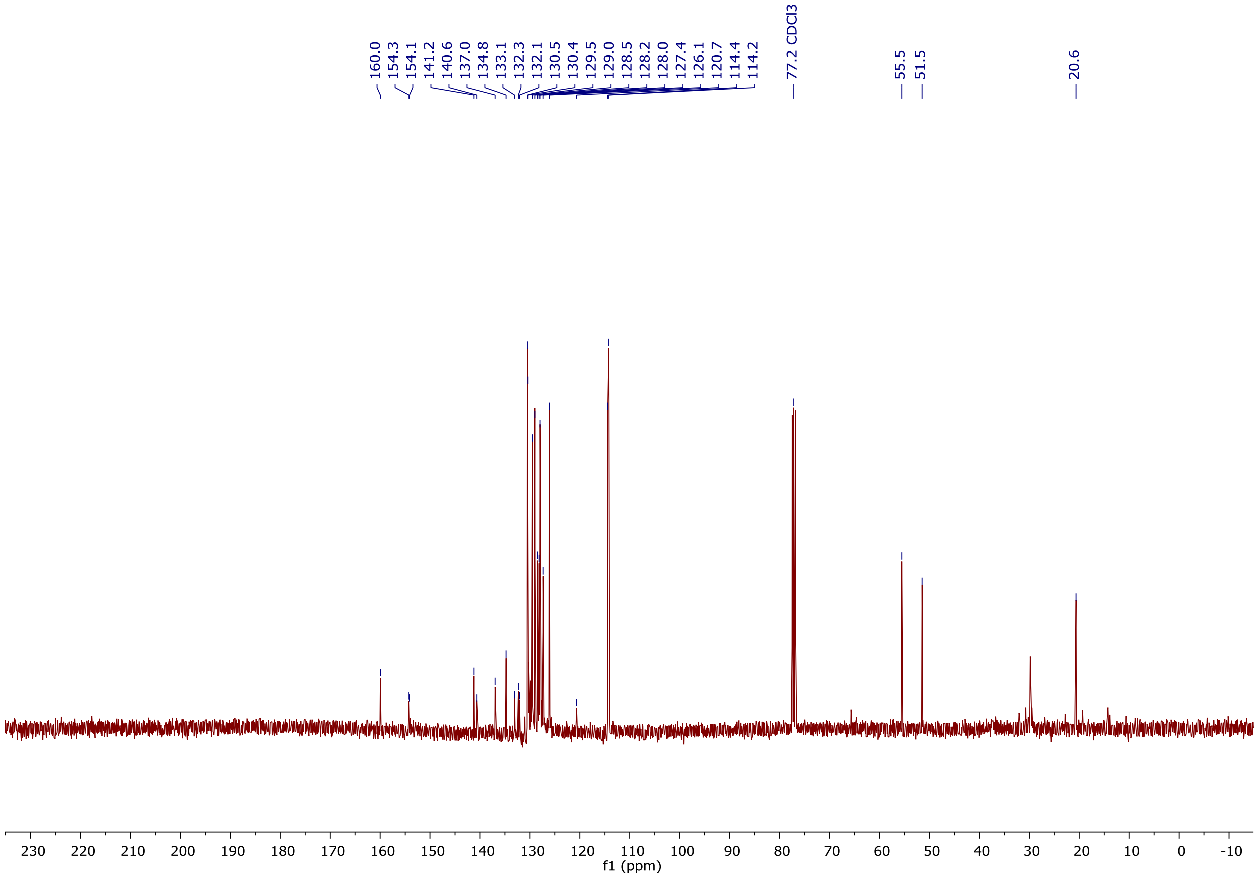

Figure S26. ^{13}C NMR (101 MHz, chloroform-*d*) spectrum of compound **5c**

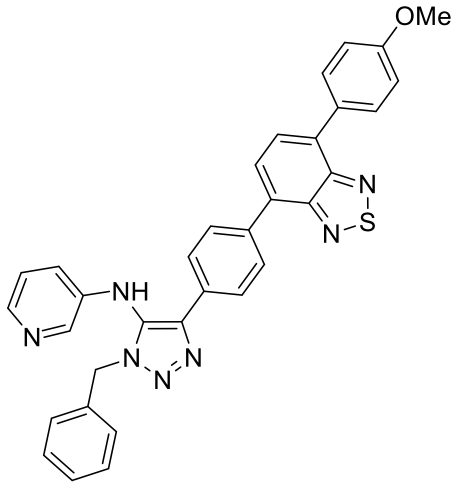

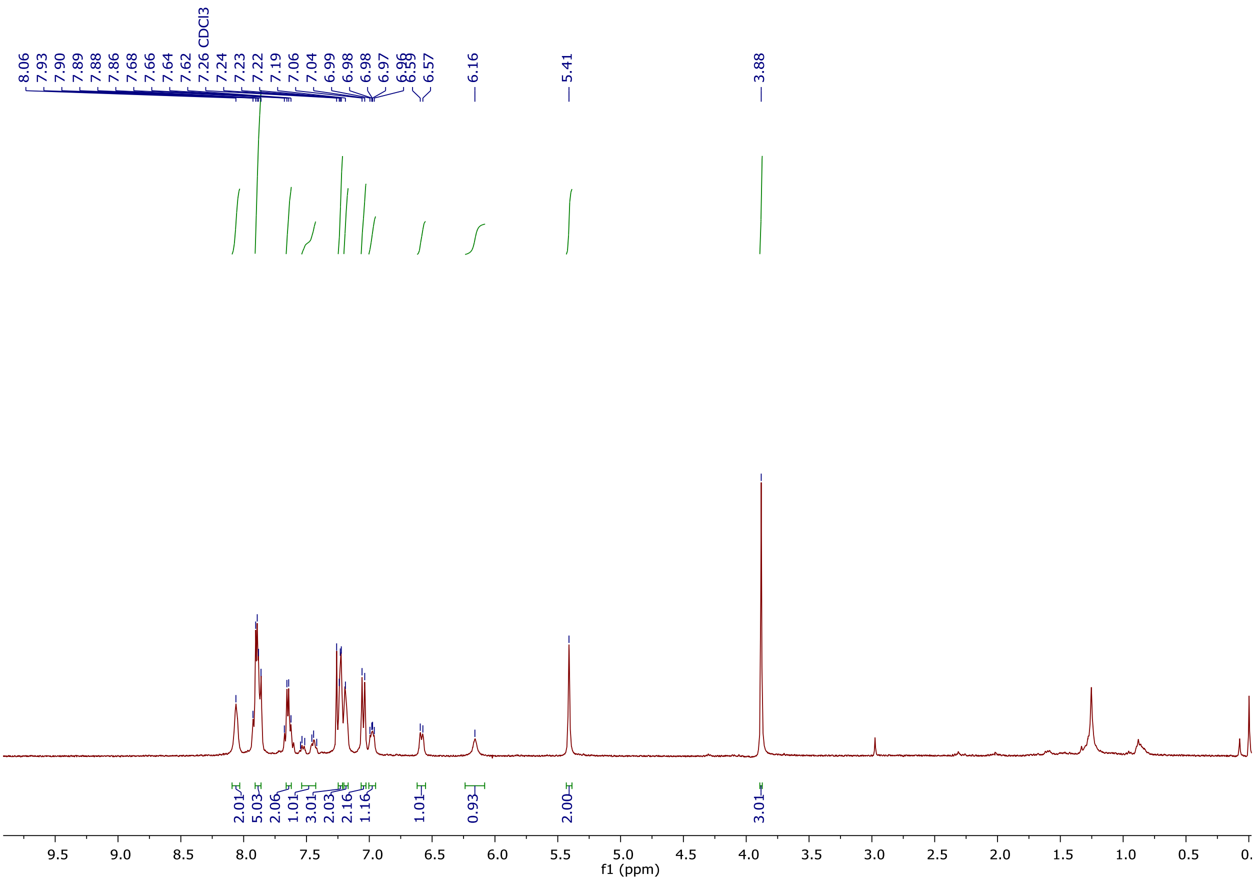

Figure S27. ^{1}H NMR (400 MHz, chloroform-*d*) spectrum of compound **5d**

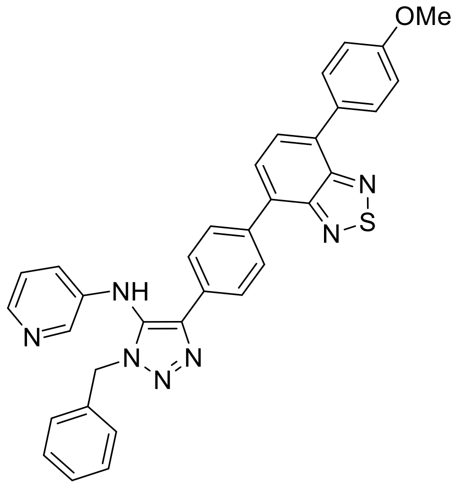

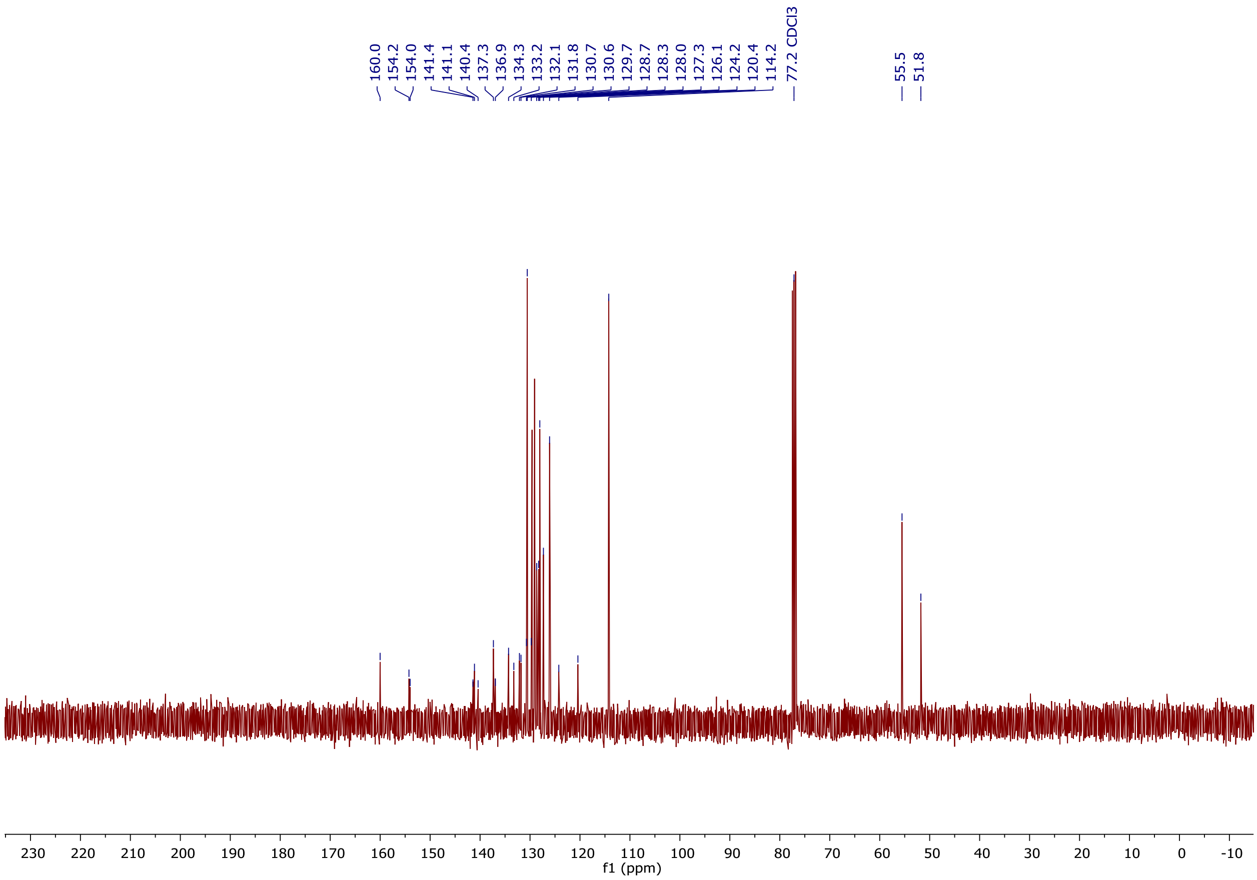

Figure S28. ^{13}C NMR (101 MHz, chloroform-*d*) spectrum of compound **5d**

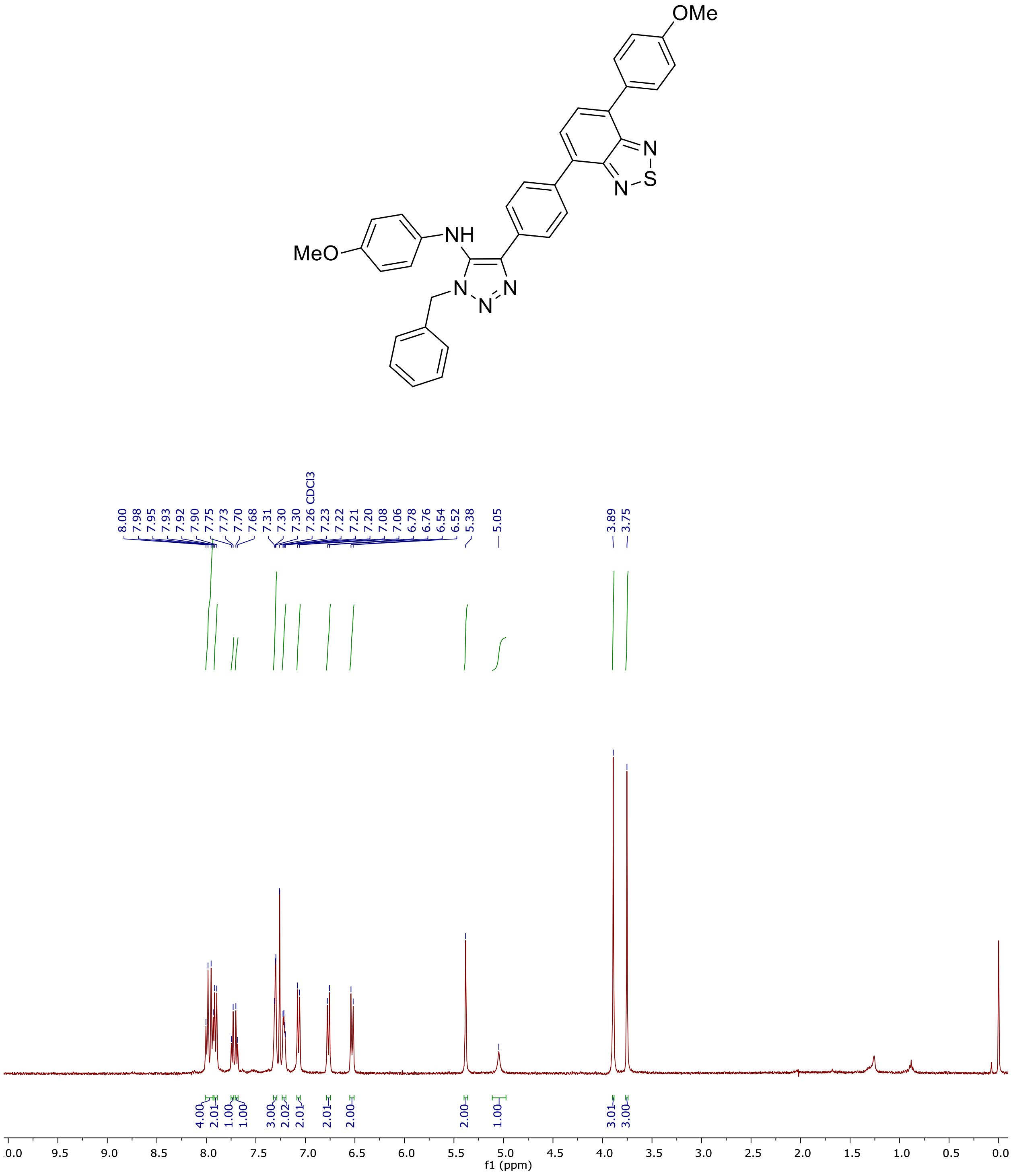

Figure S29. ^{1}H NMR (400 MHz, chloroform-*d*) spectrum of compound **5e**

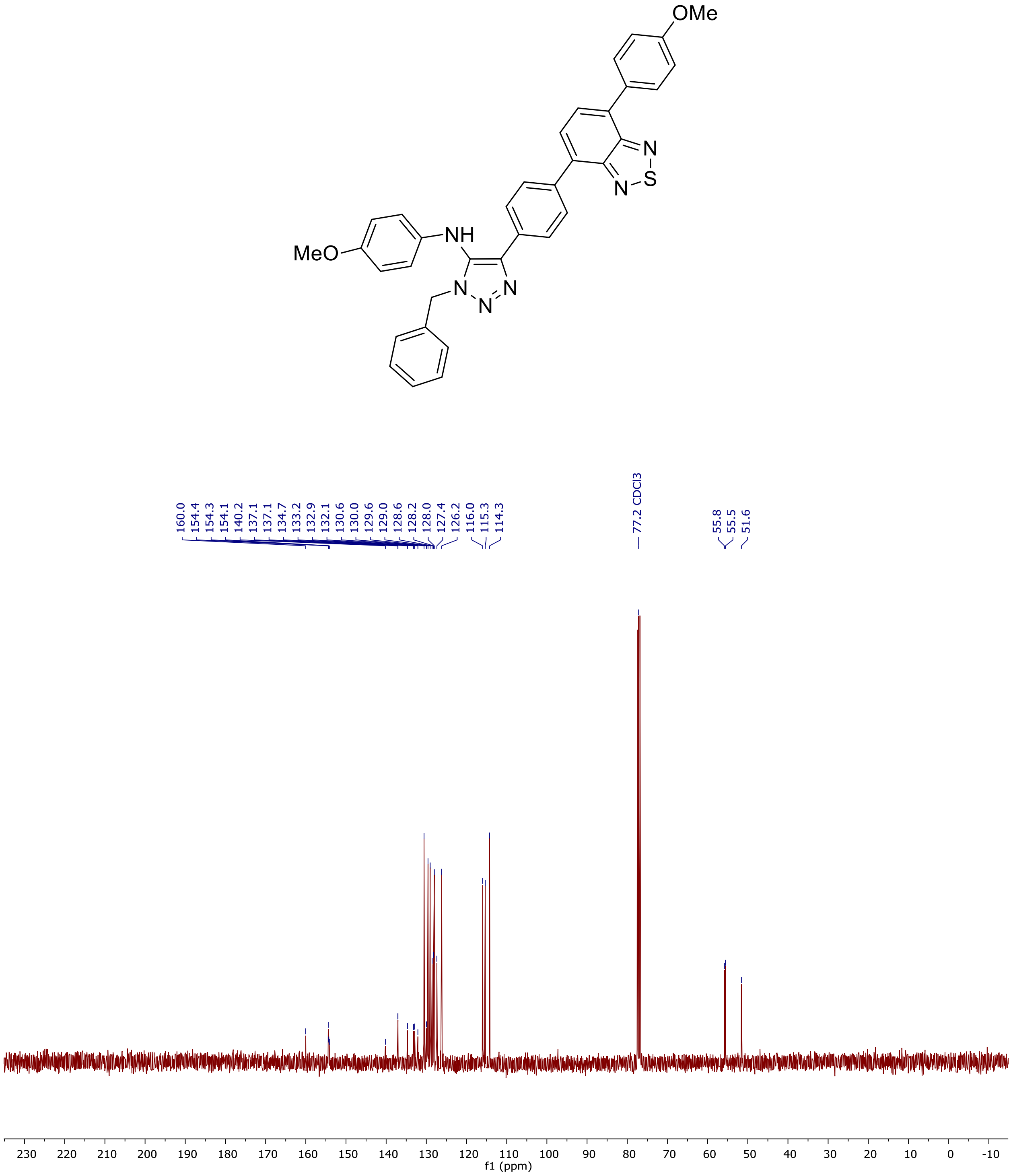

Figure S30. ^{13}C NMR (101 MHz, chloroform-*d*) spectrum of compound **5e**

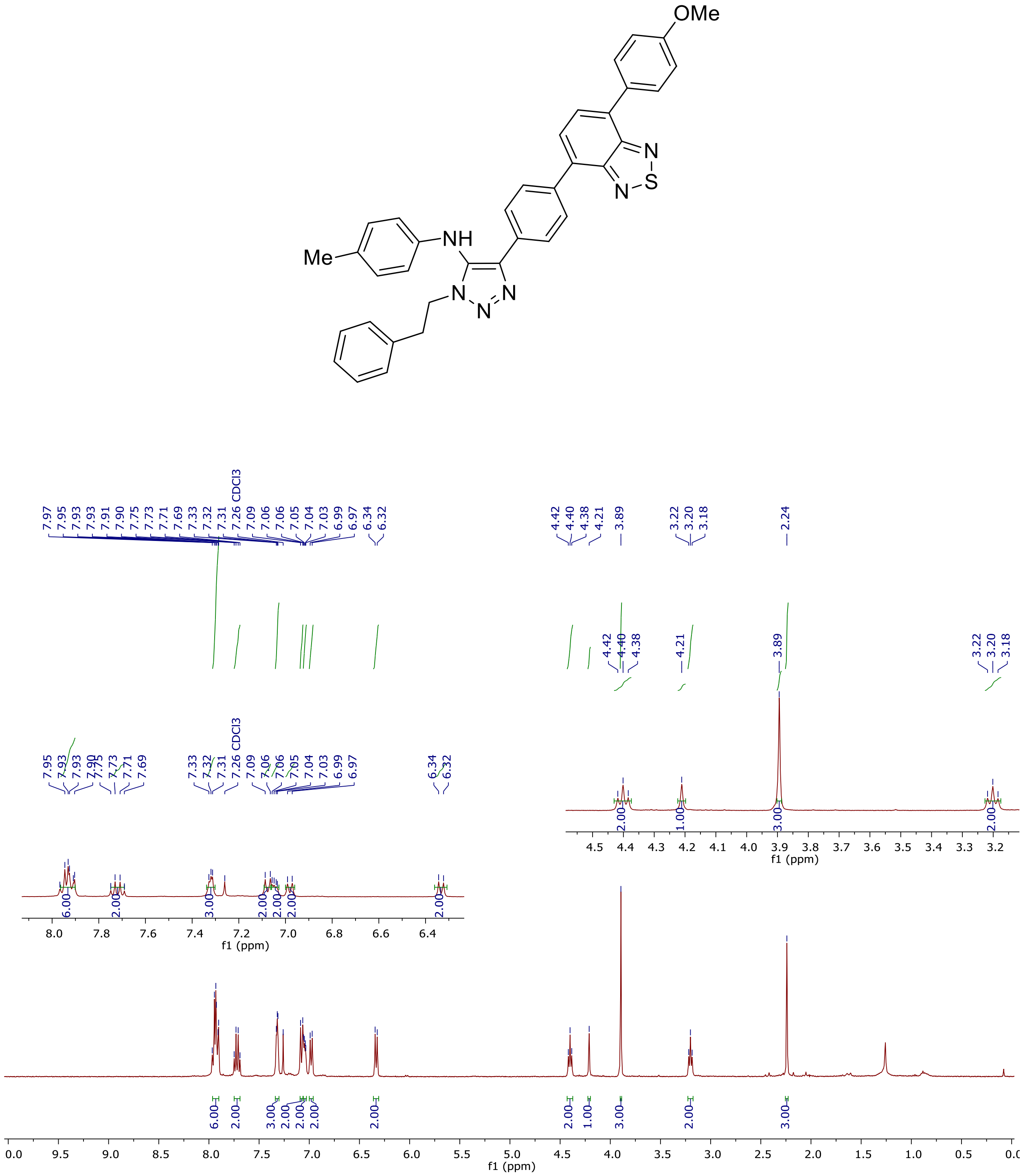

Figure S31. ^{1}H NMR (400 MHz, chloroform-*d*) spectrum of compound **5f**

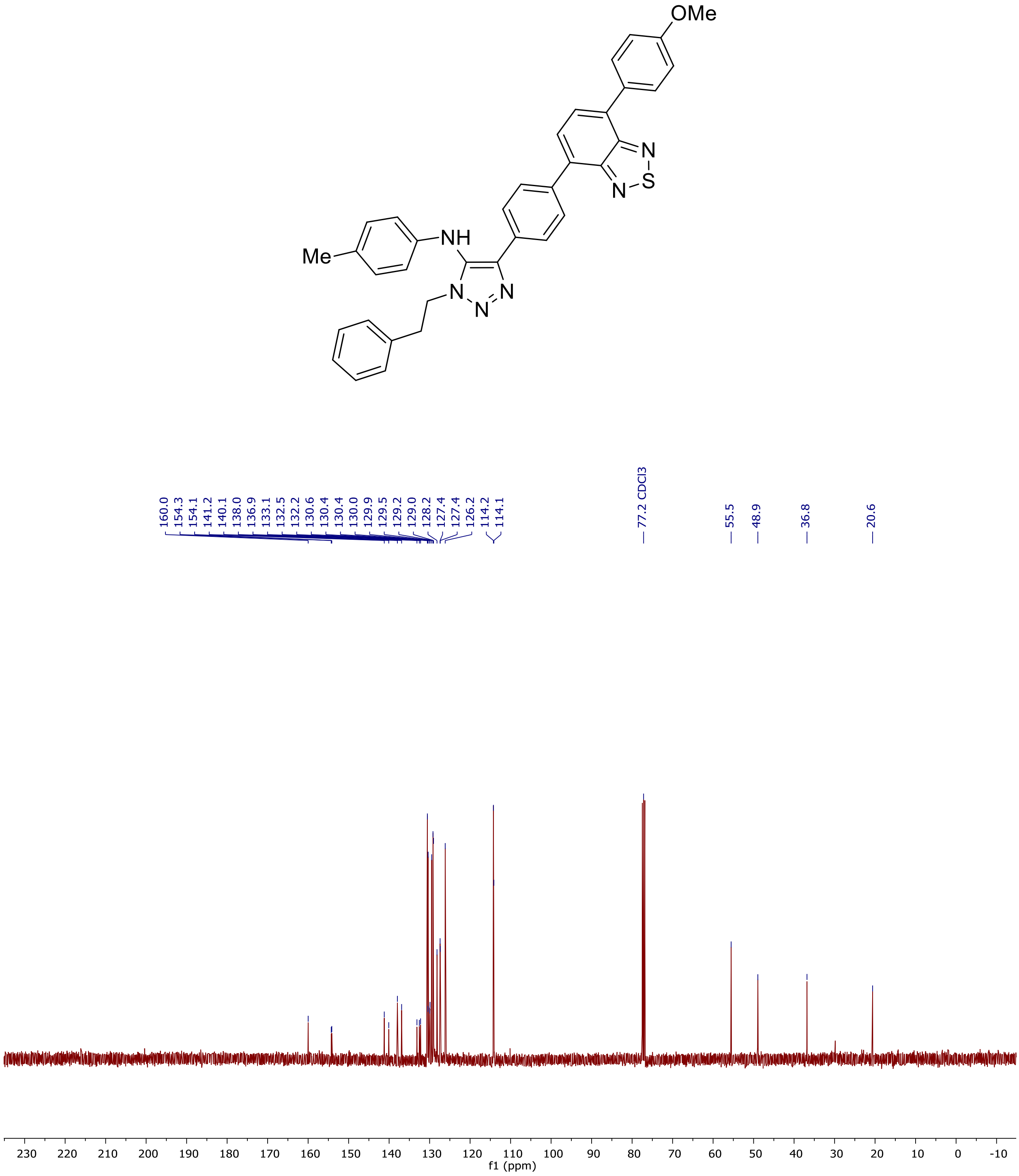

Figure S32. ^{13}C NMR (101 MHz, chloroform-*d*) spectrum of compound **5f**

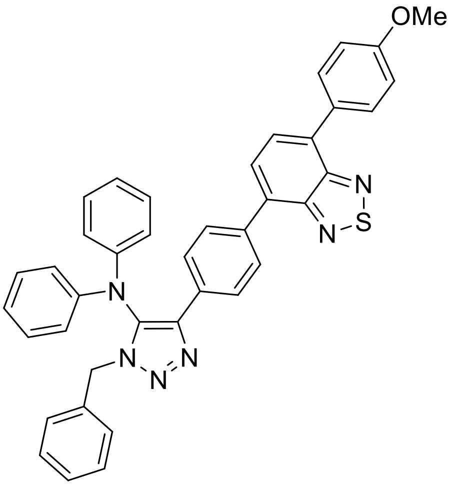

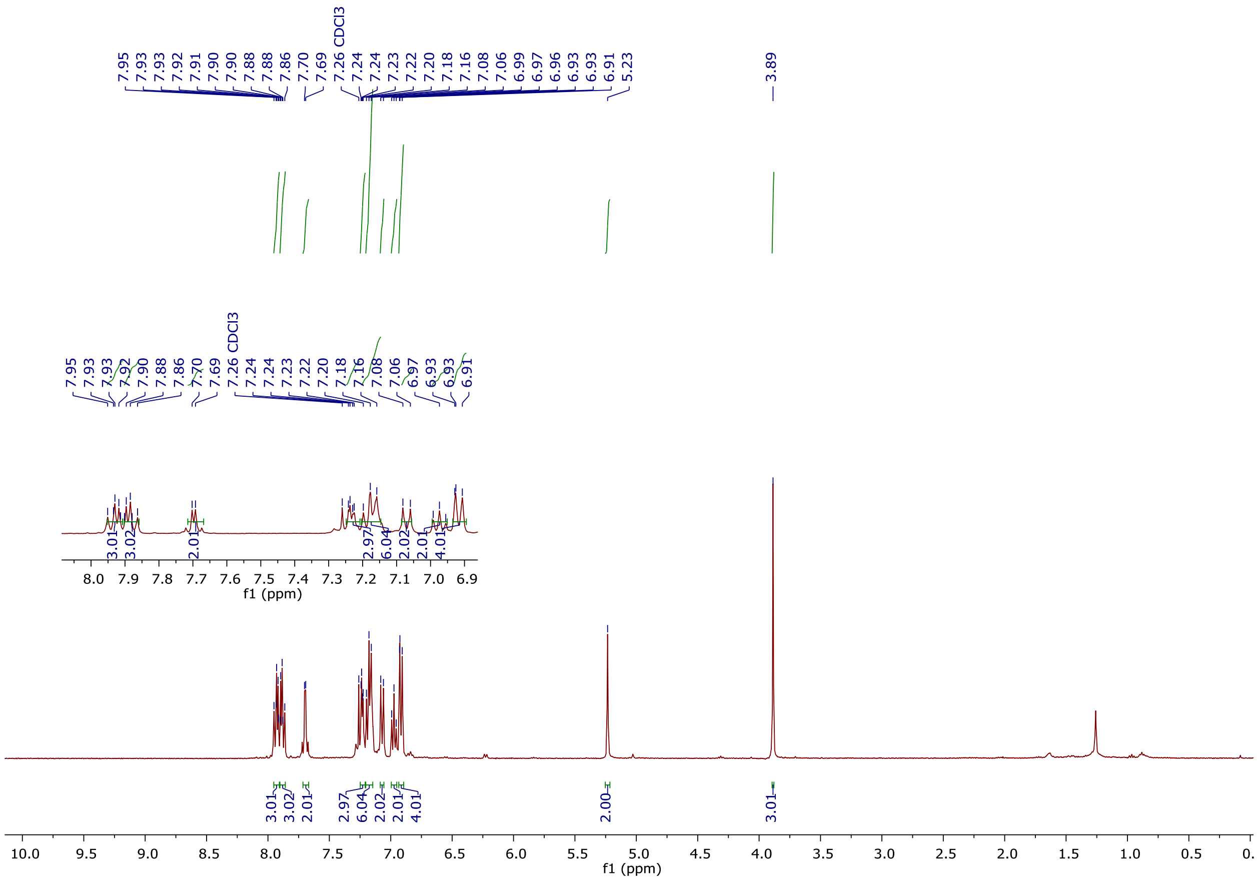

Figure S33. ^{1}H NMR (400 MHz, chloroform-*d*) spectrum of compound **6a**

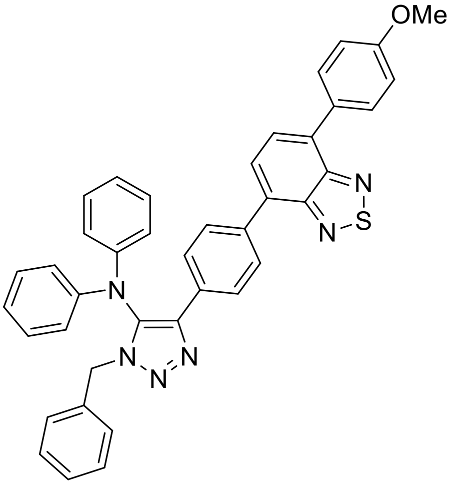

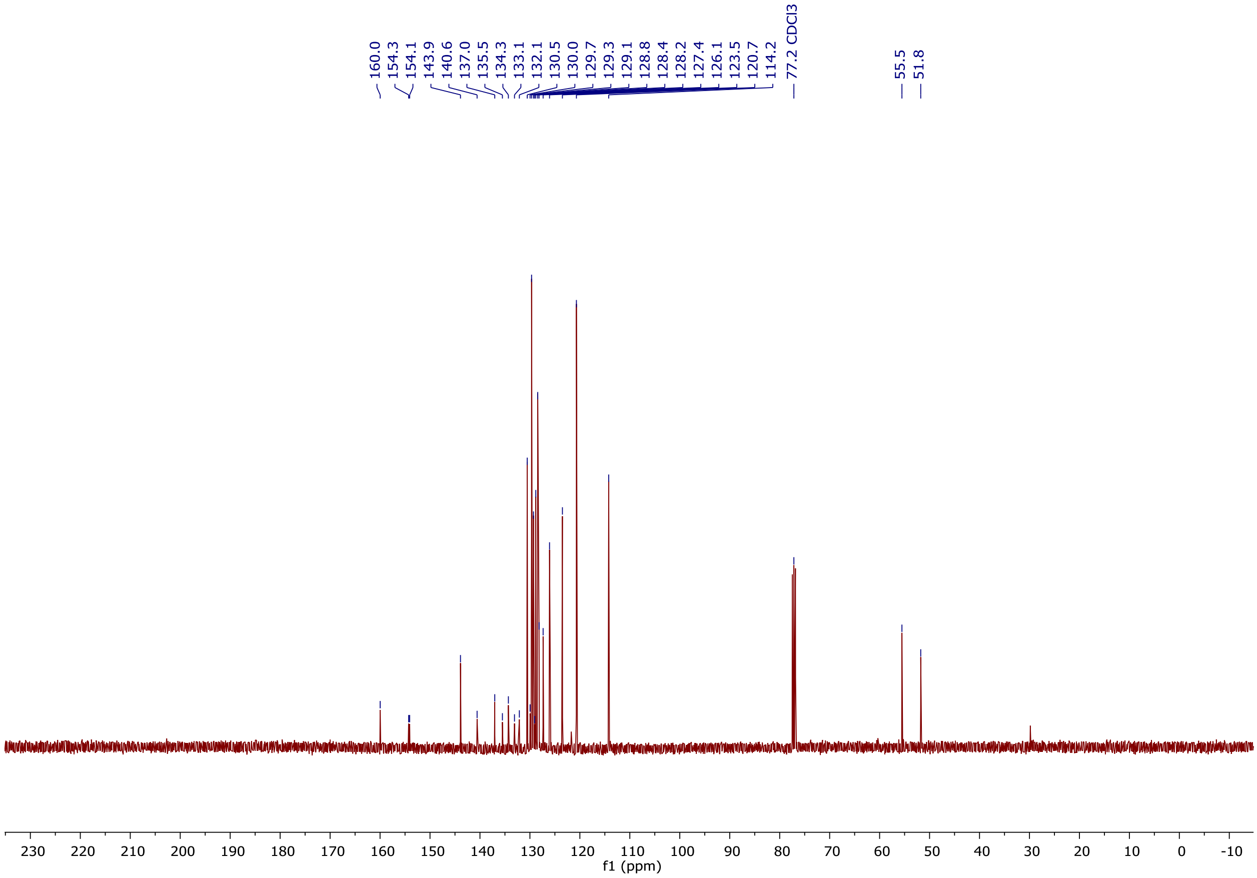

Figure S34. ^{13}C NMR (101 MHz, chloroform-*d*) spectrum of compound **6a**

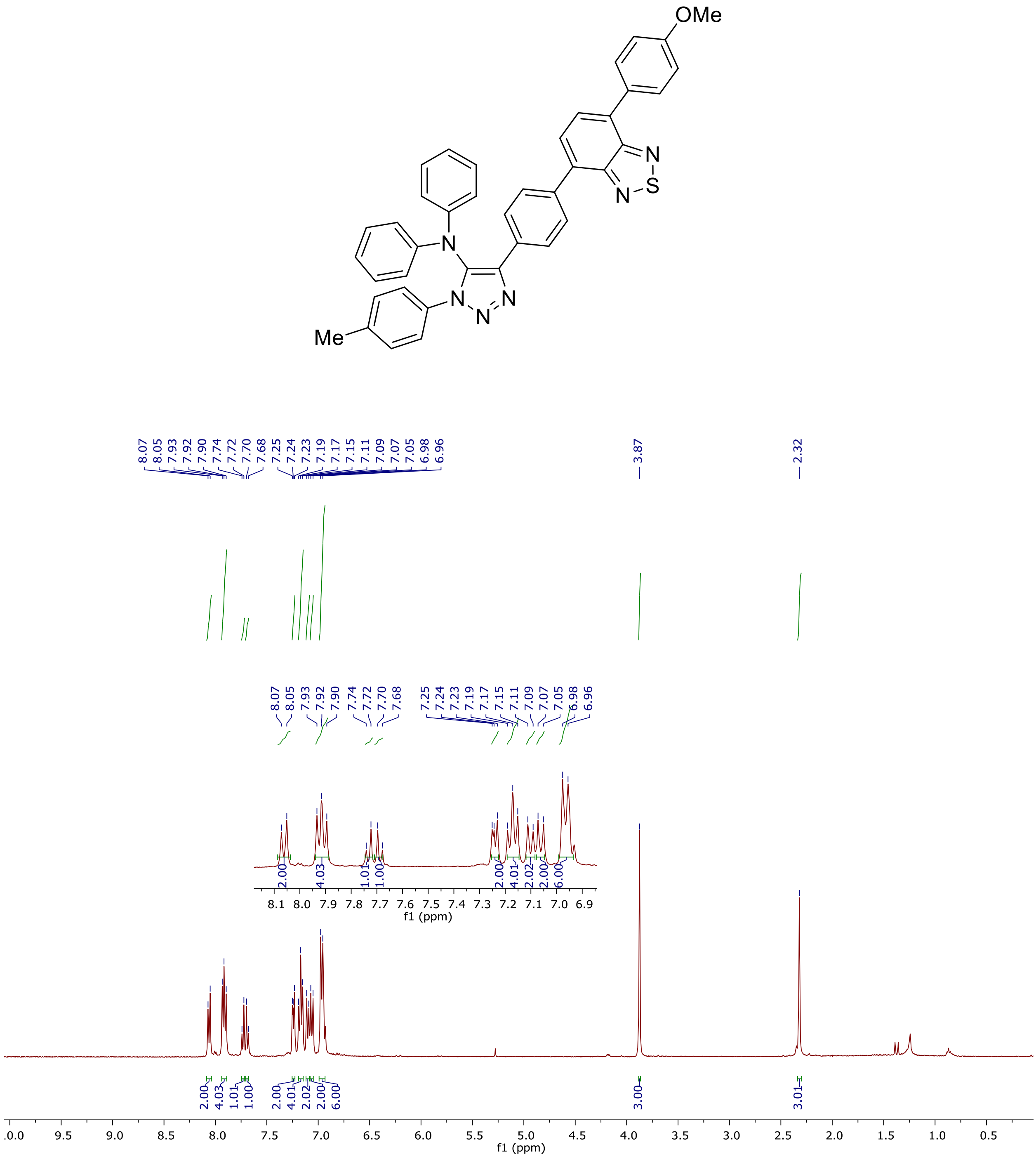

Figure S35. ^{1}H NMR (400 MHz, chloroform-*d*) spectrum of compound **6b**

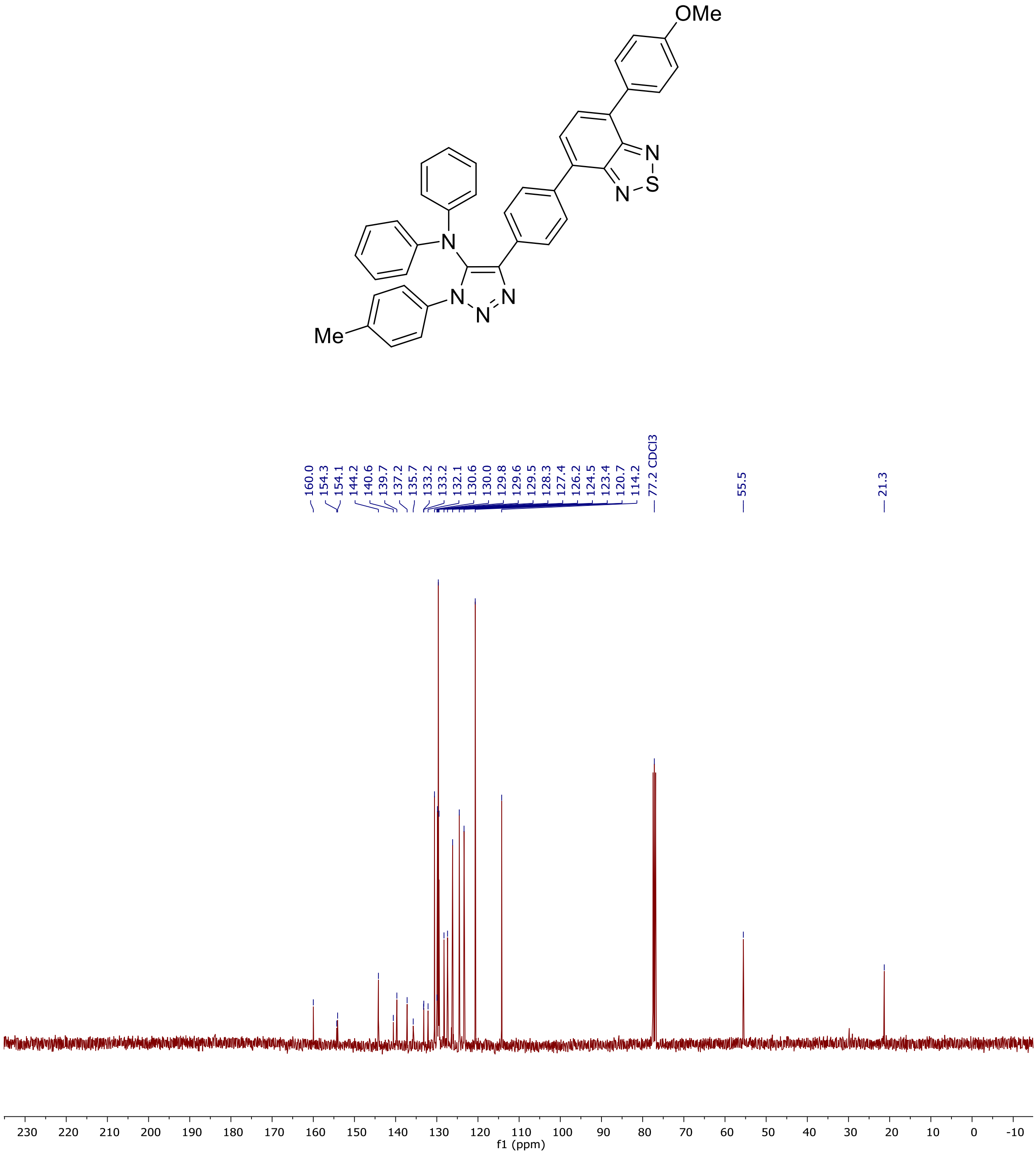

Figure S36. ^{13}C NMR (101 MHz, chloroform-*d*) spectrum of compound **6b**

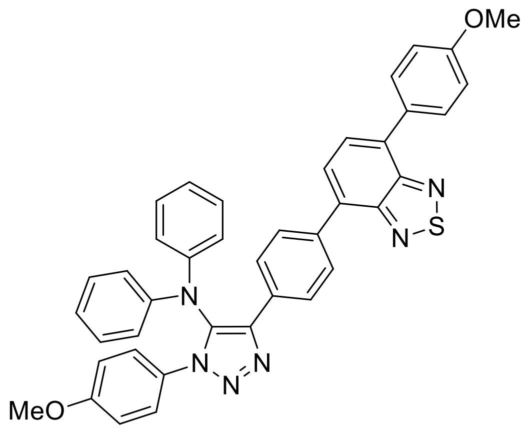

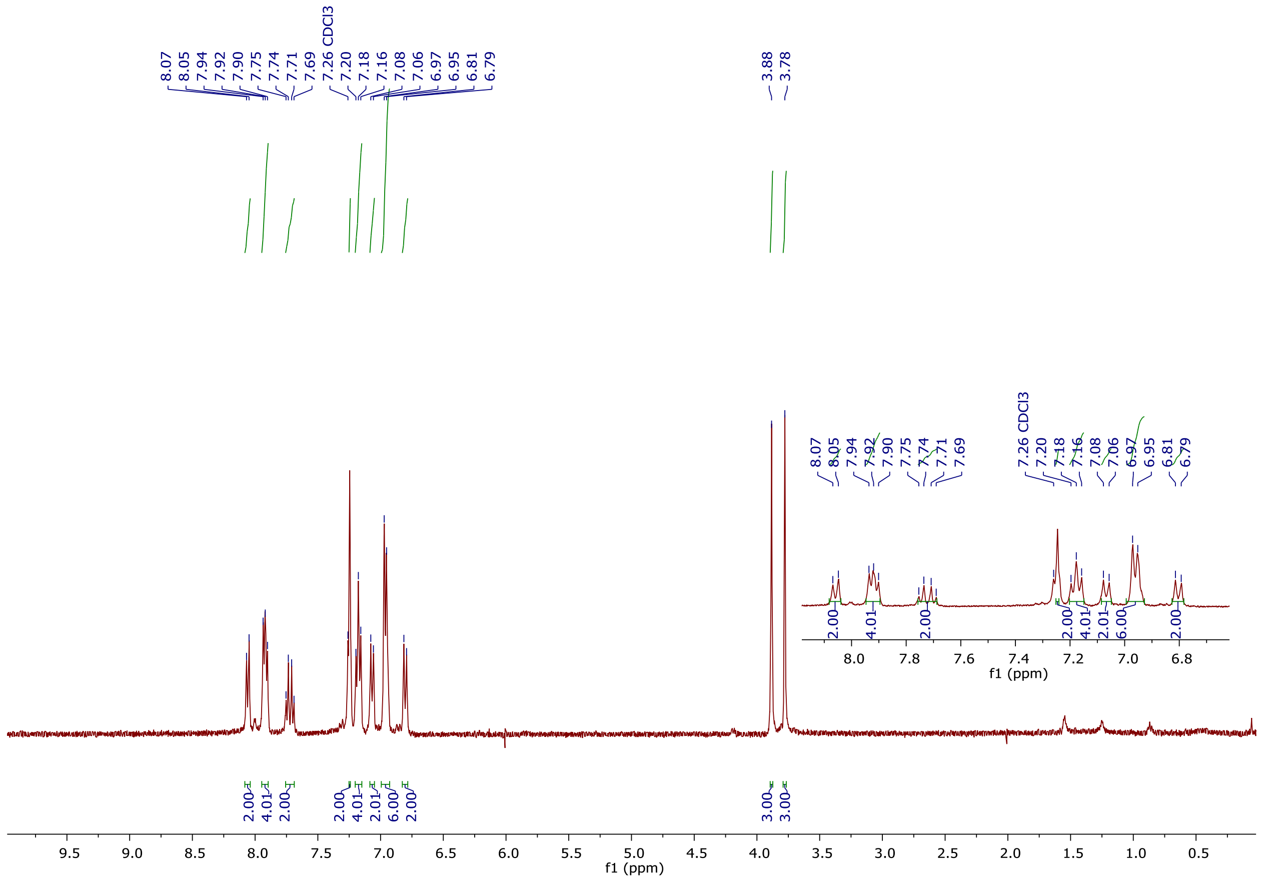

Figure S37. ^{1}H NMR (400 MHz, chloroform-*d*) spectrum of compound **6c**

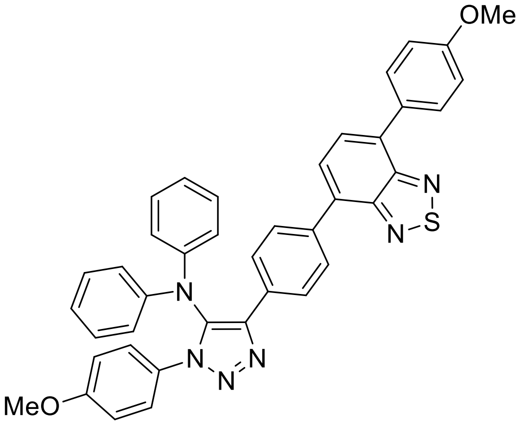

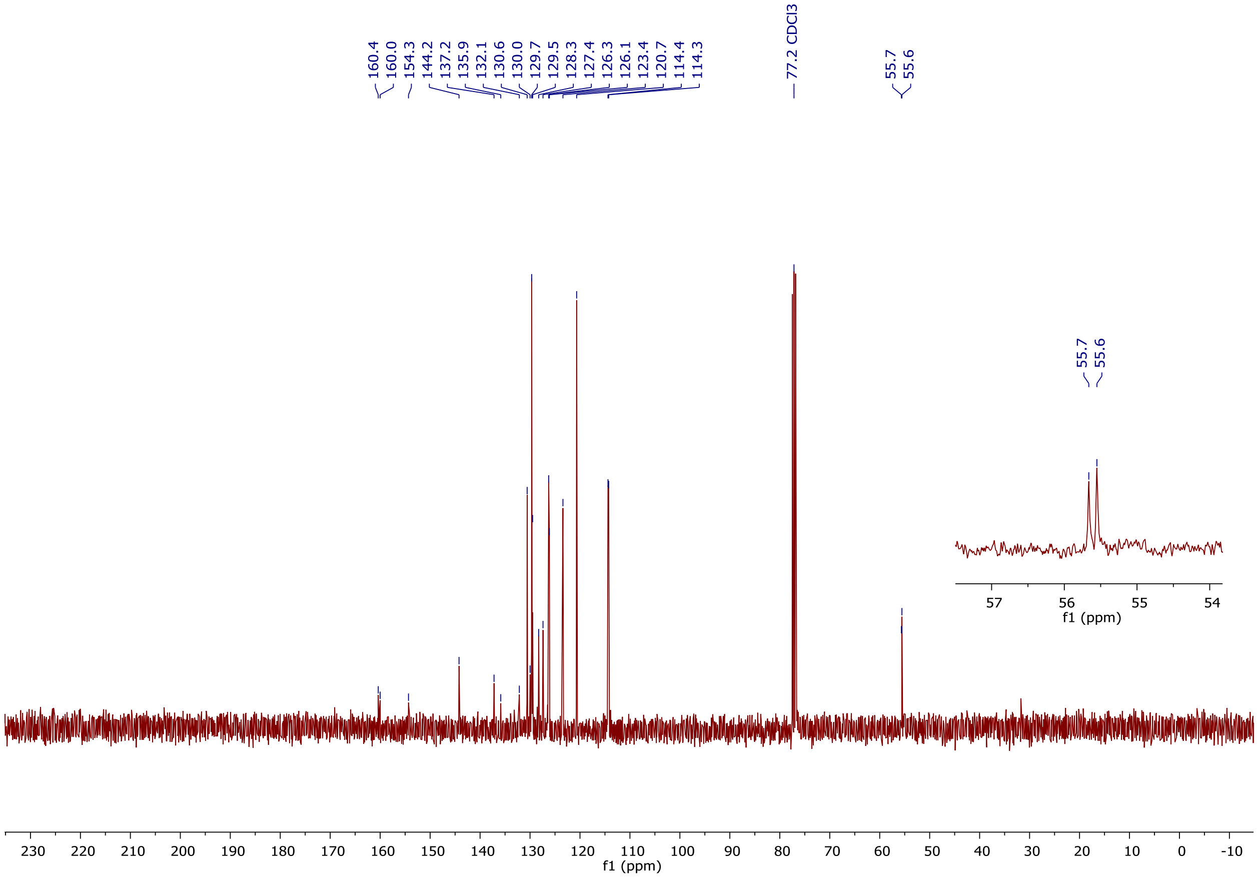

Figure S38. ^{13}C NMR (101 MHz, chloroform-*d*) spectrum of compound **6c**

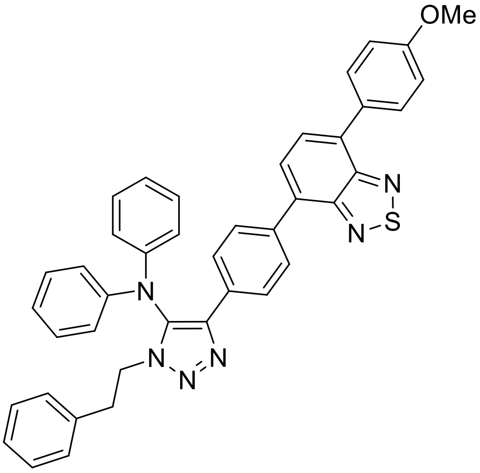

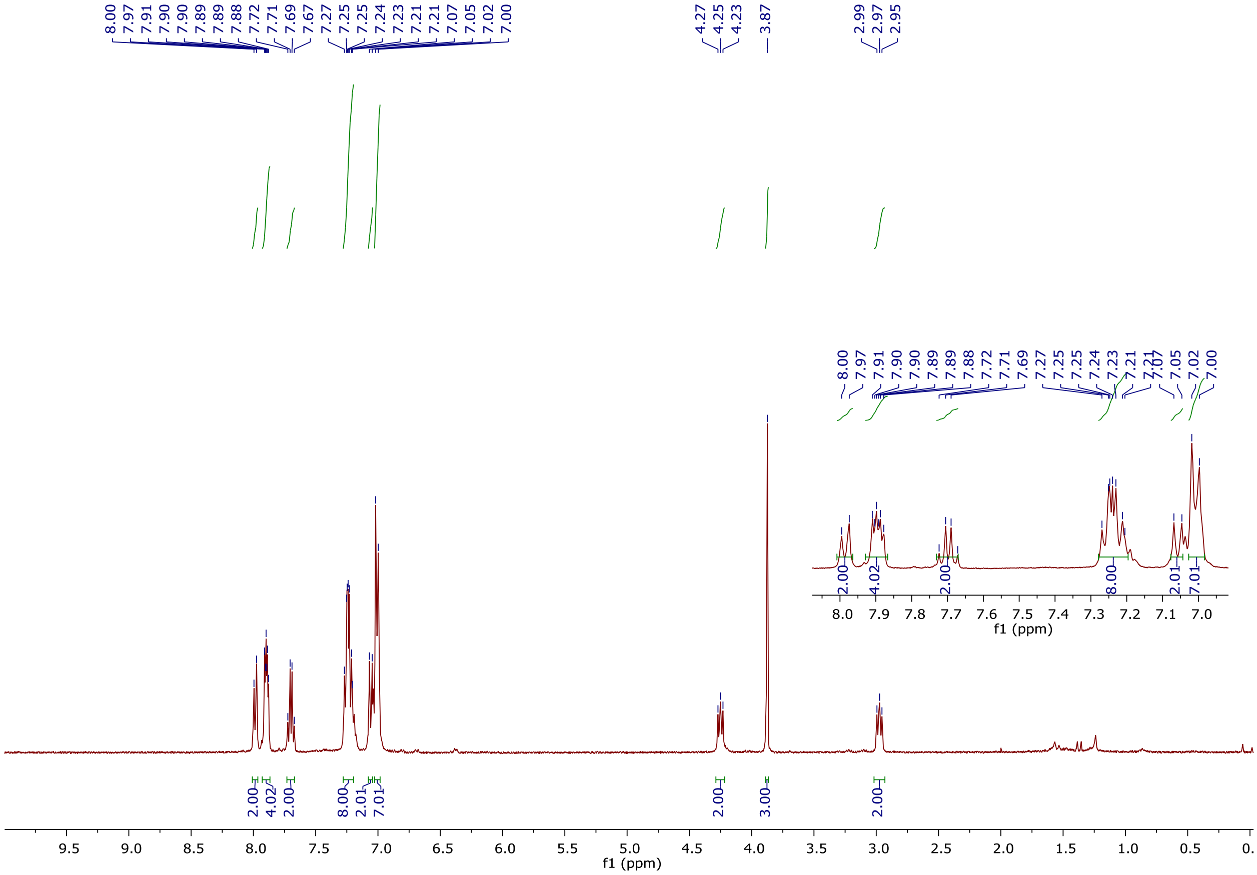

Figure S39. ^{1}H NMR (400 MHz, chloroform-*d*) spectrum of compound **6d**

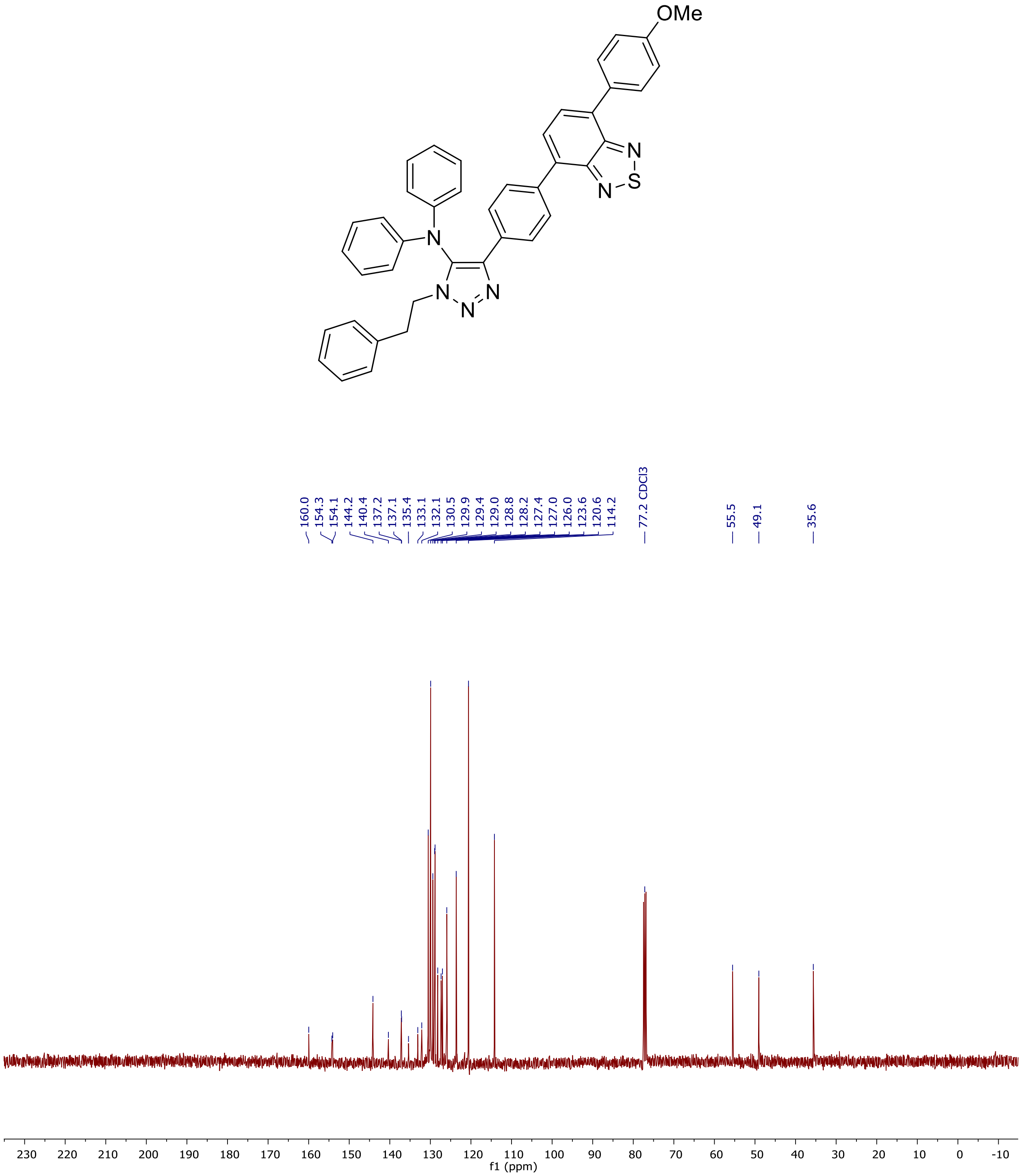

Figure S40. ^{13}C NMR (101 MHz, chloroform-*d*) spectrum of compound **6d**

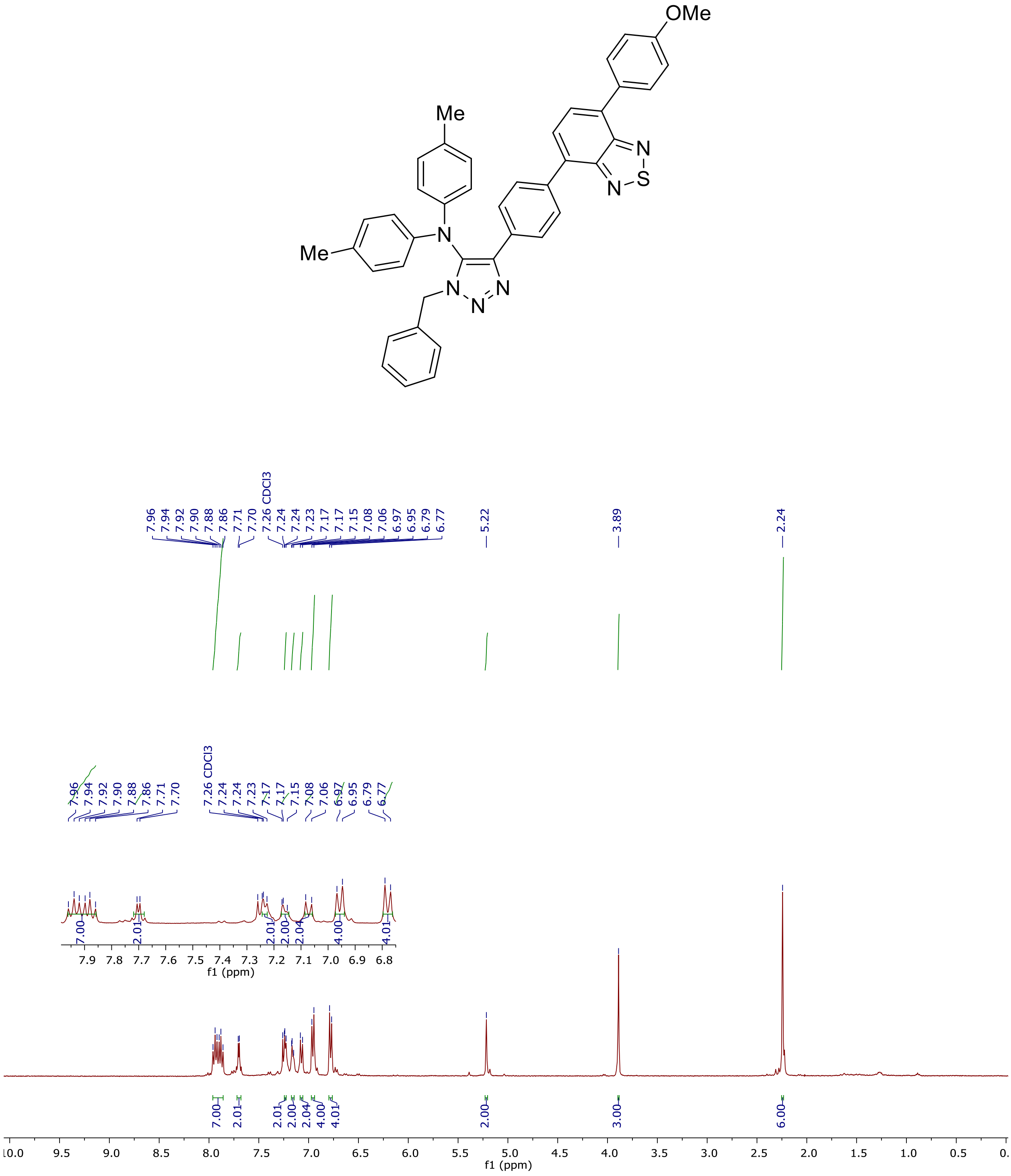

Figure S41. ^{1}H NMR (400 MHz, chloroform-*d*) spectrum of compound **6e**

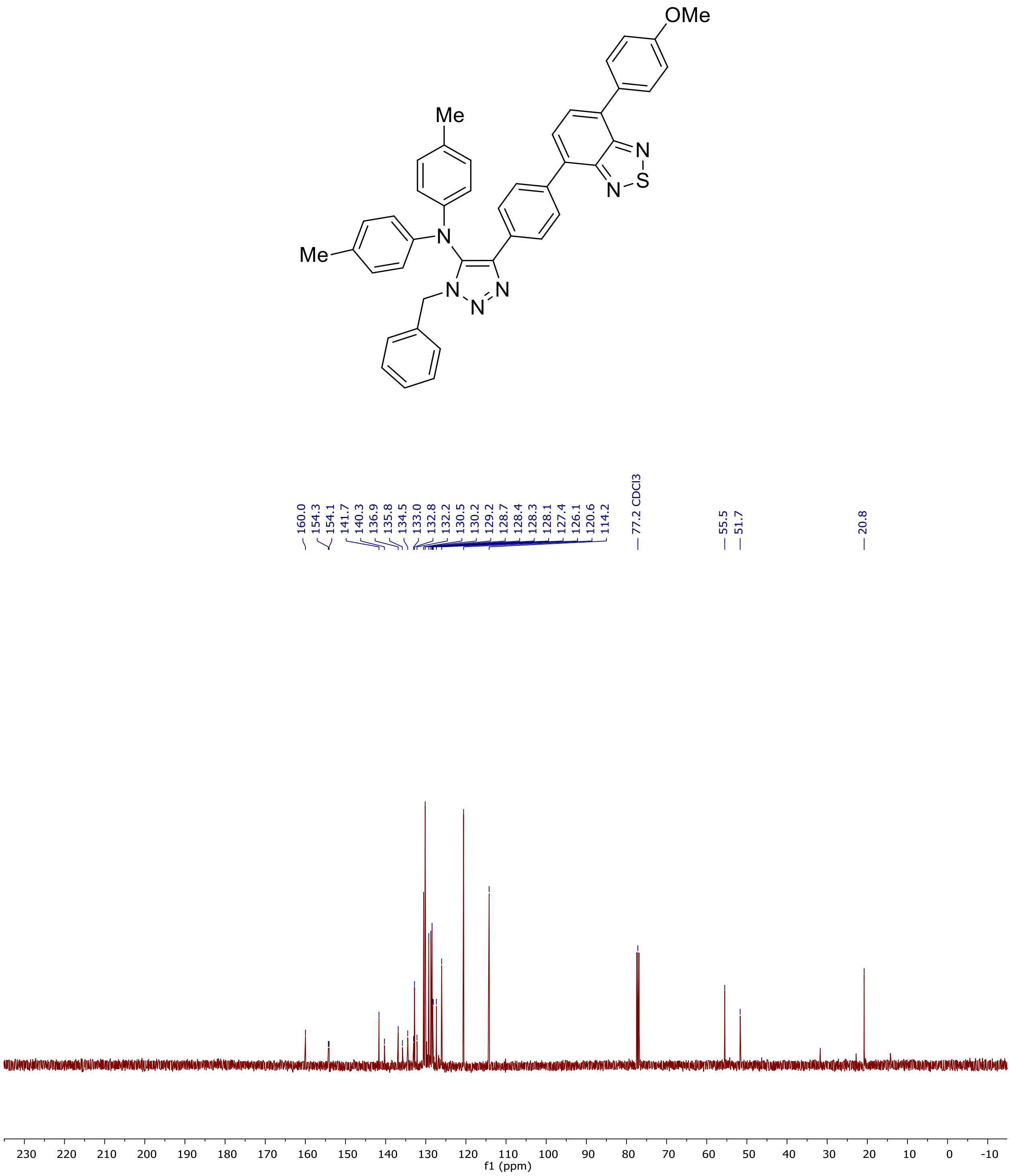

Figure S42. ^{13}C NMR (101 MHz, chloroform-*d*) spectrum of compound **6e**

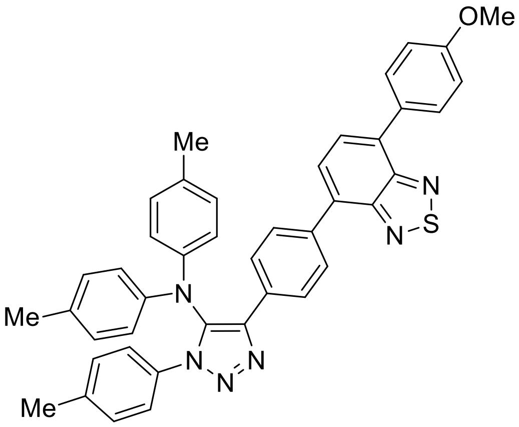

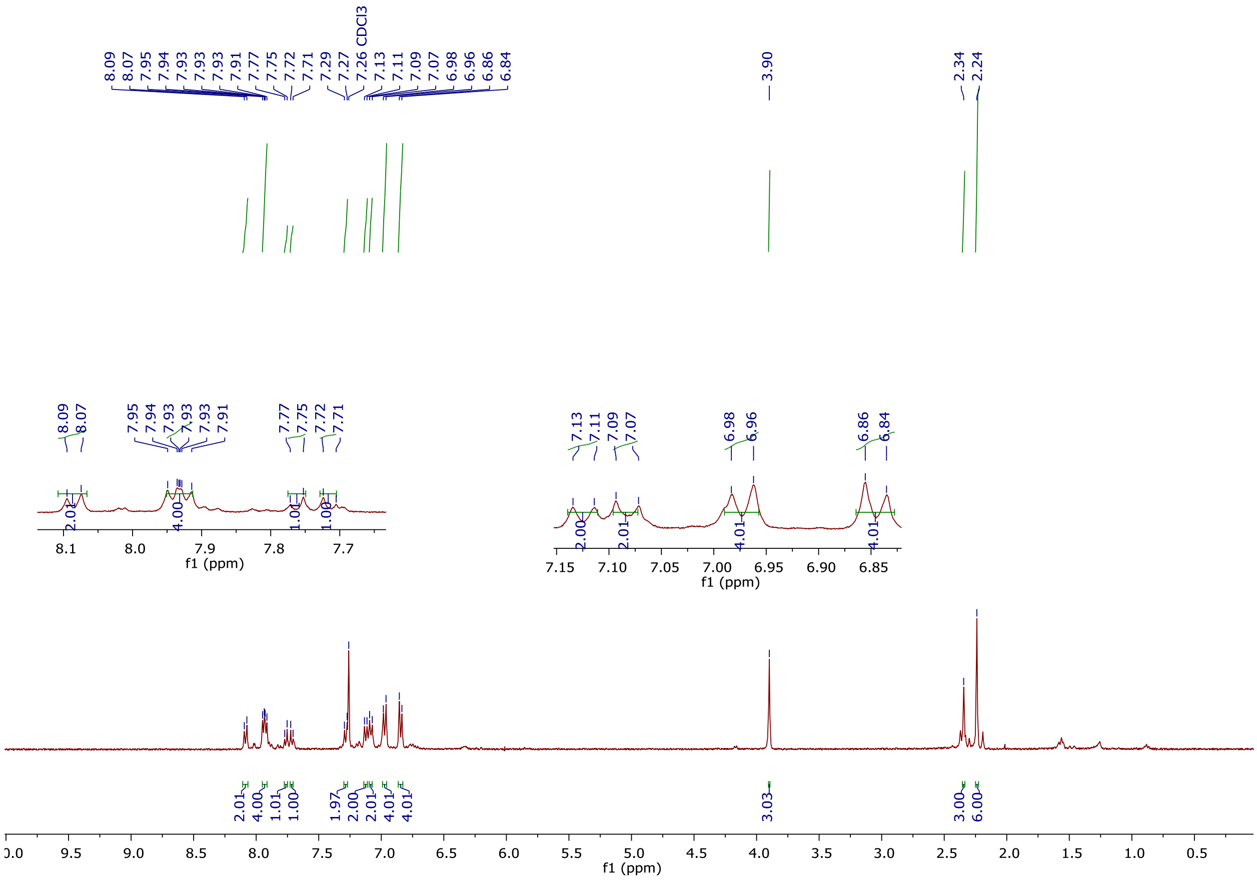

Figure S43. ^{1}H NMR (400 MHz, chloroform-*d*) spectrum of compound **6f**

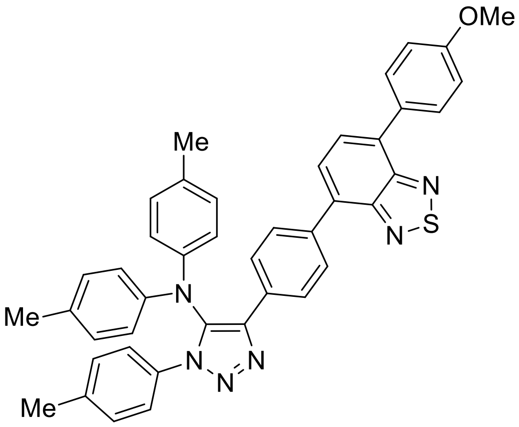

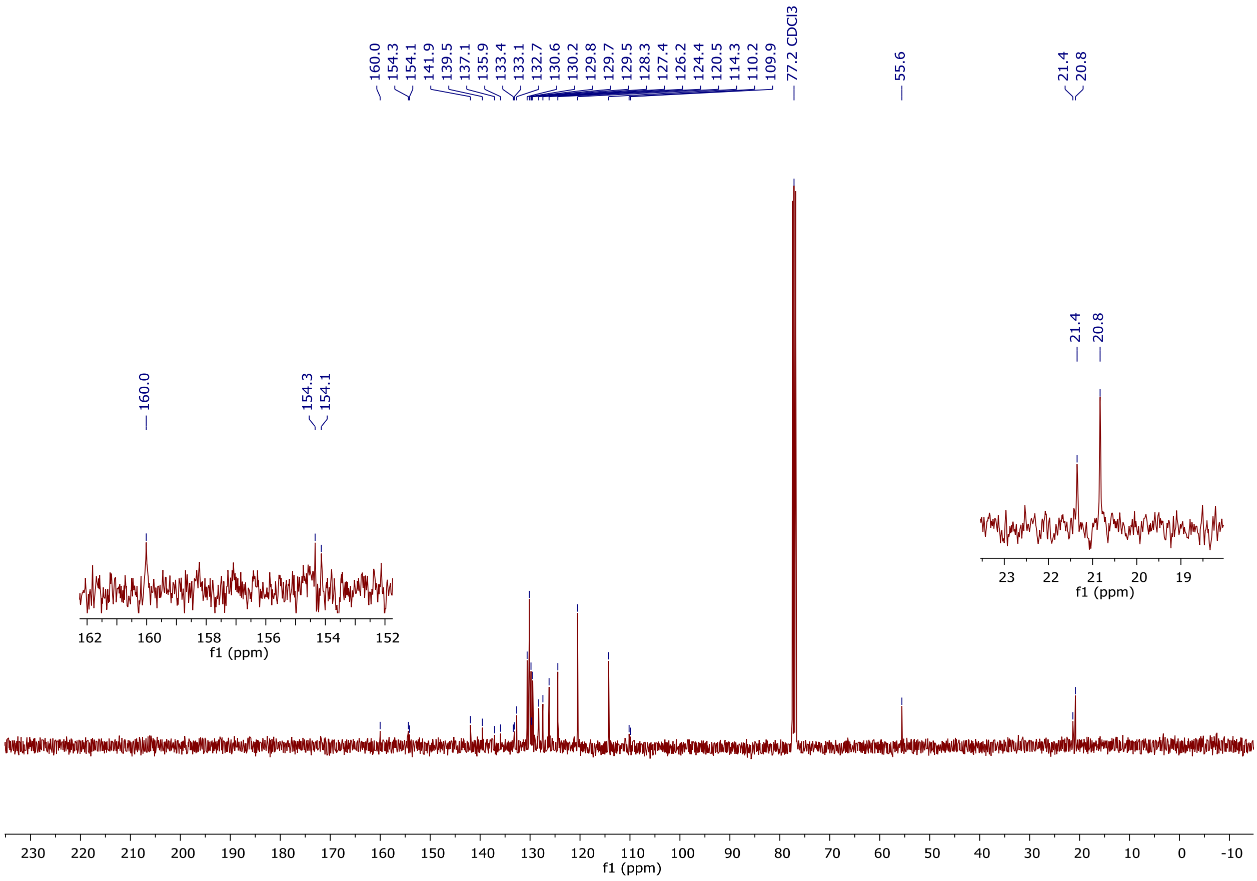

Figure S44. ^{13}C NMR (101 MHz, chloroform-*d*) spectrum of compound **6f**

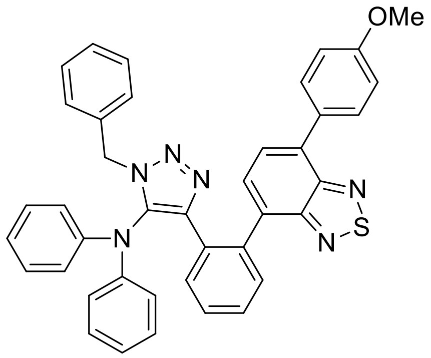

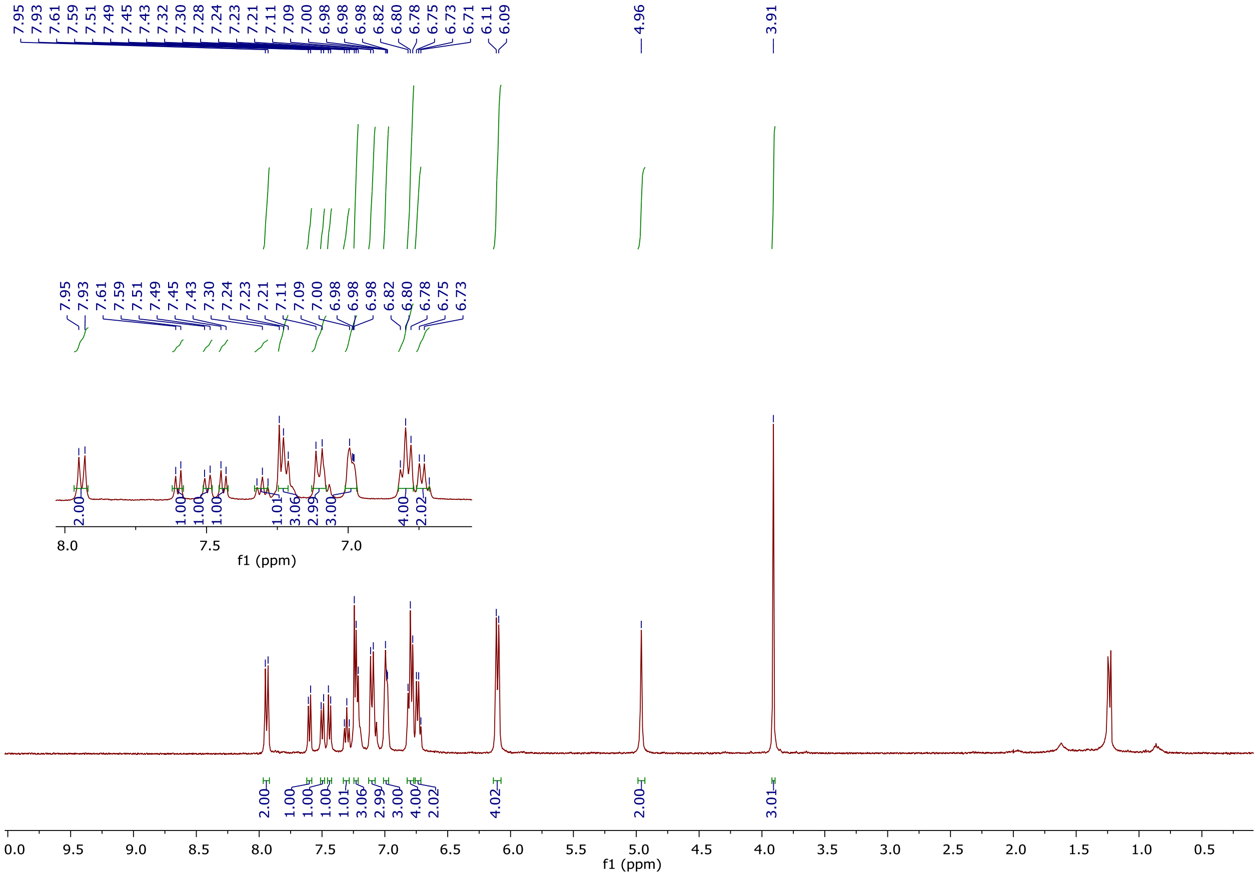

Figure S45. ^{1}H NMR (400 MHz, chloroform-*d*) spectrum of compound **6g**

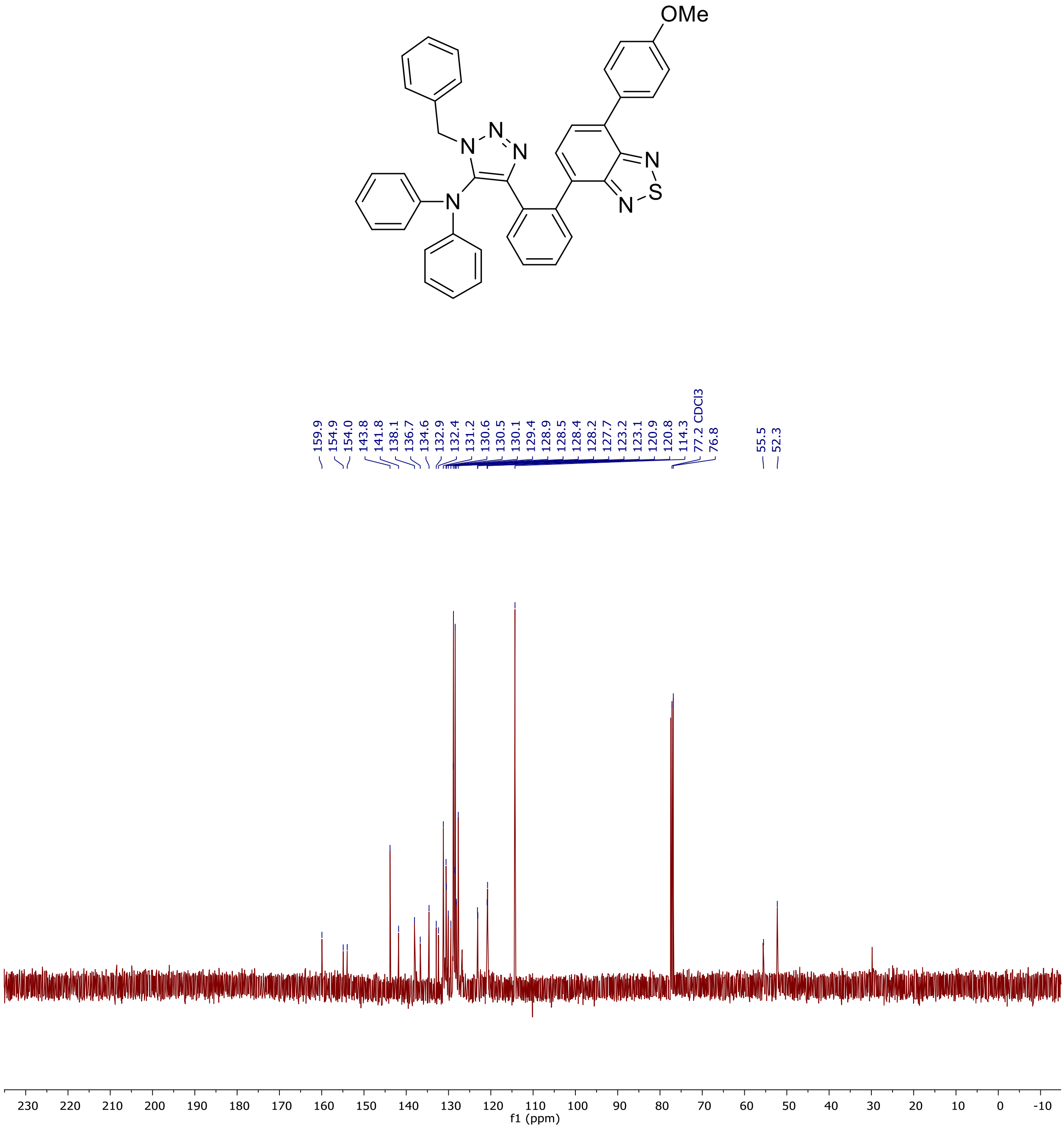

Figure S46. ^{13}C NMR (101 MHz, chloroform-*d*) spectrum of compound **6g**

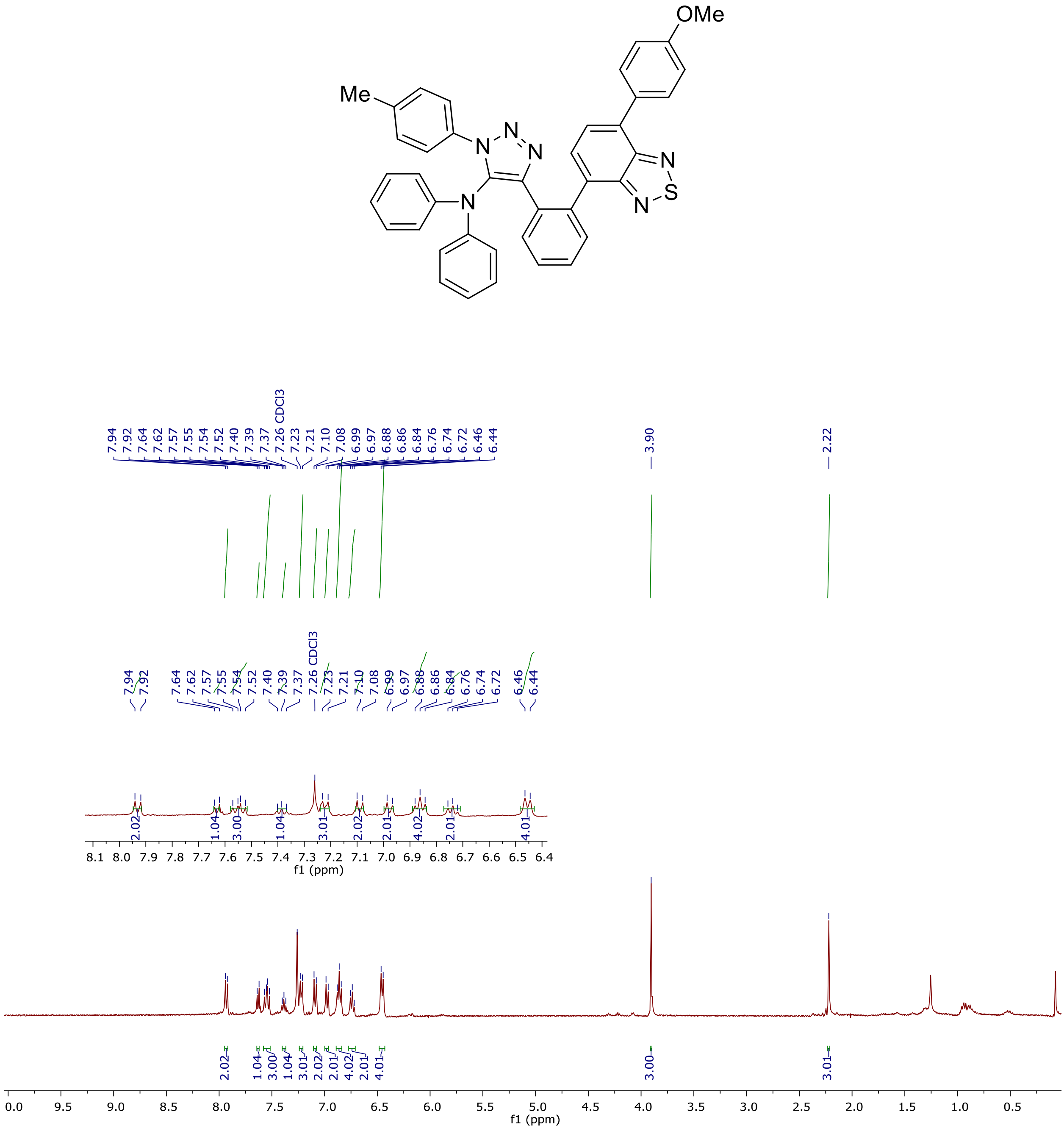

Figure S47. ^{1}H NMR (400 MHz, chloroform-*d*) spectrum of compound **6h**

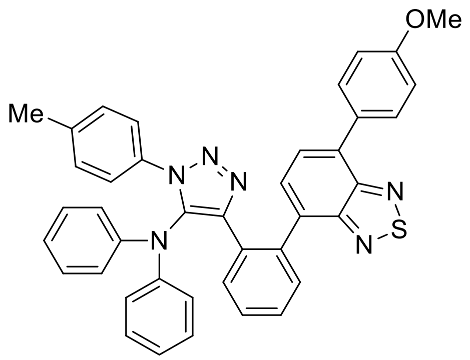

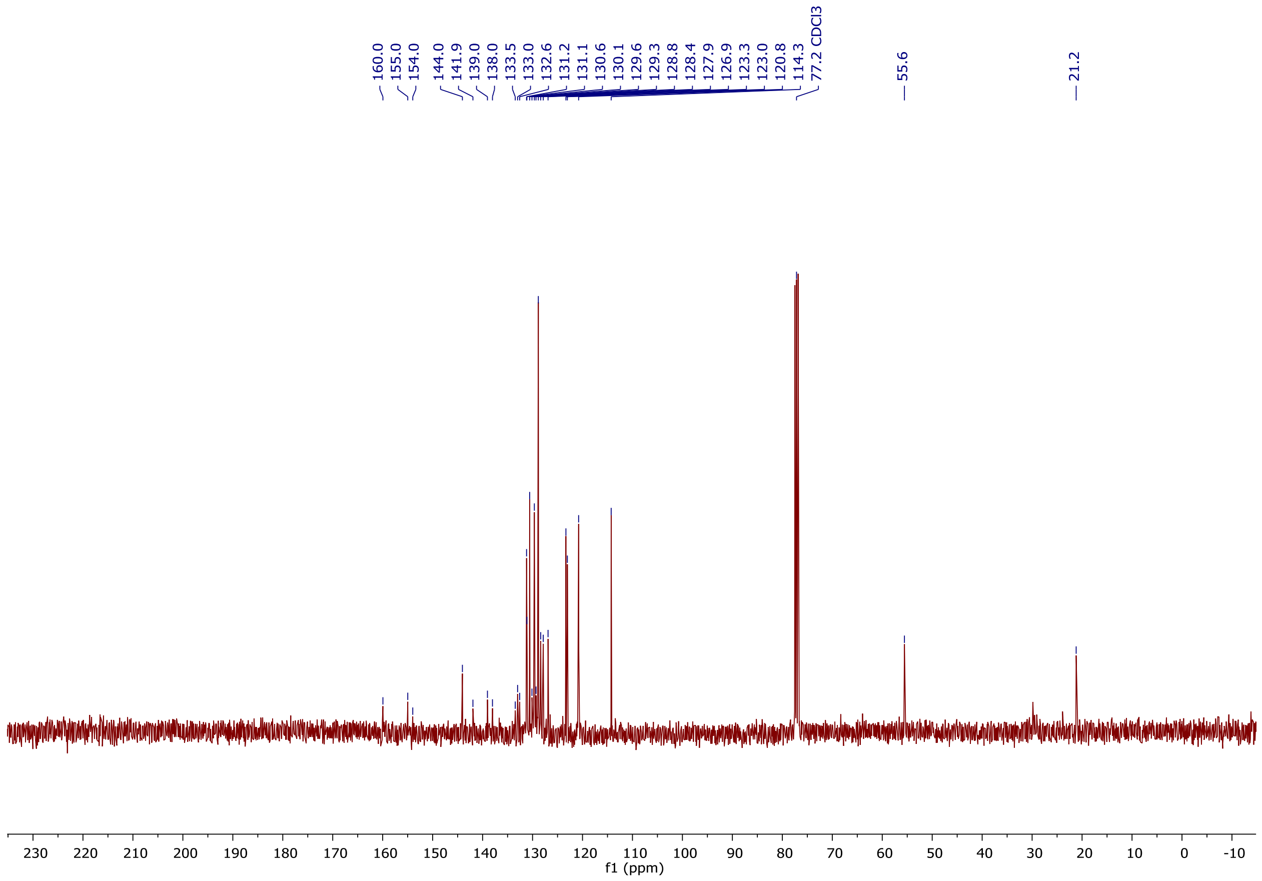

Figure S48. ^{13}C NMR (101 MHz, chloroform-*d*) spectrum of compound **6h**

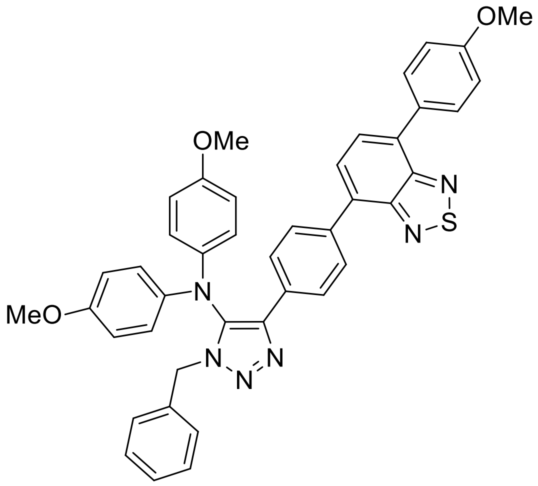

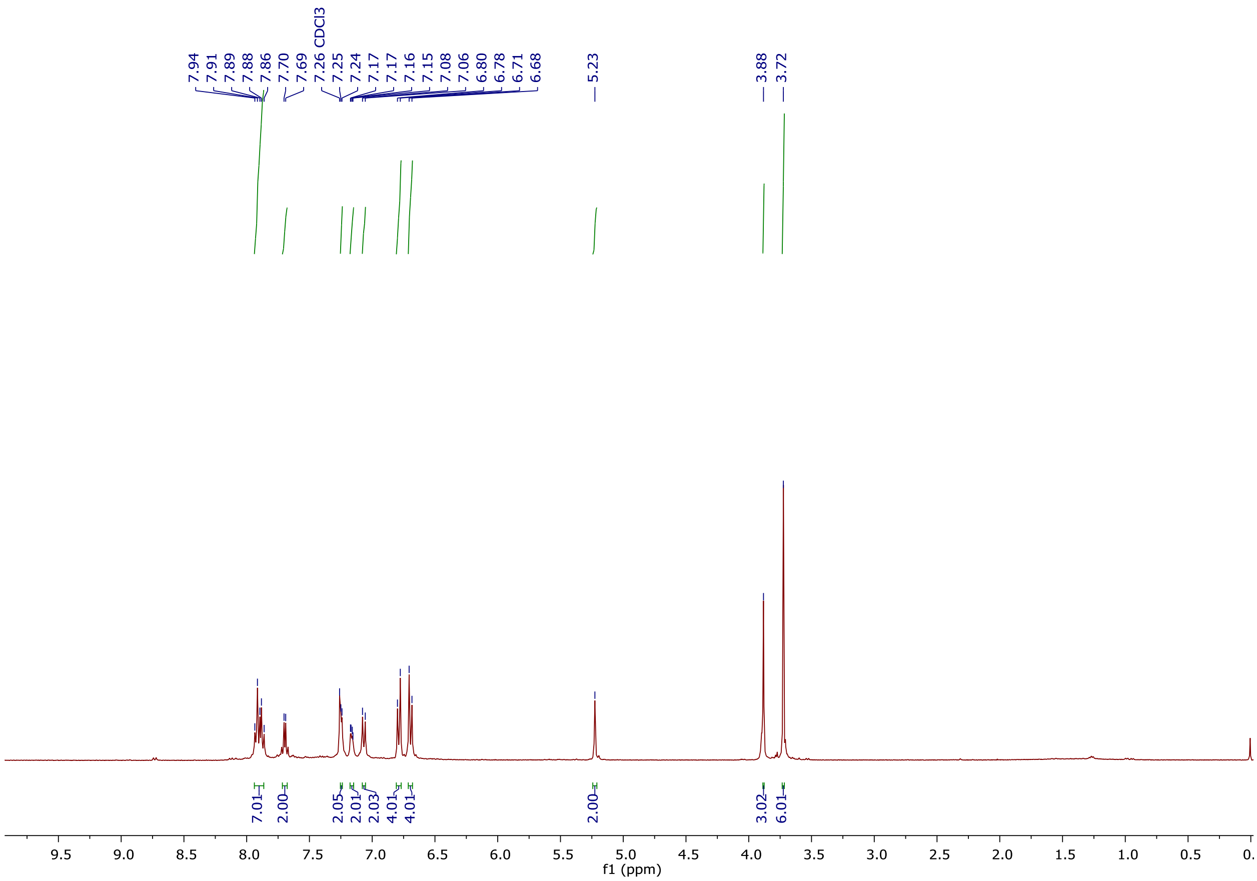

Figure S49. ^{1}H NMR (400 MHz, chloroform-*d*) spectrum of compound **6i**

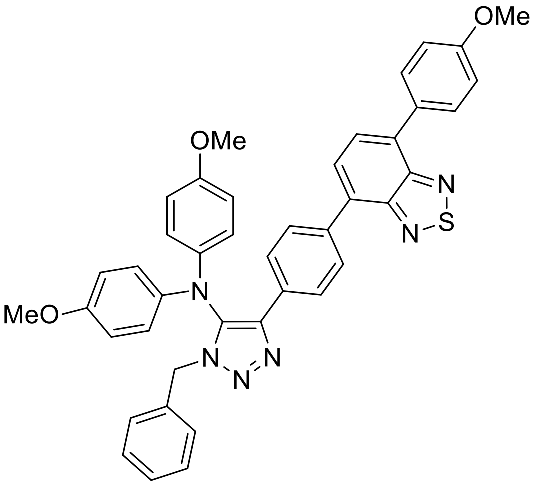

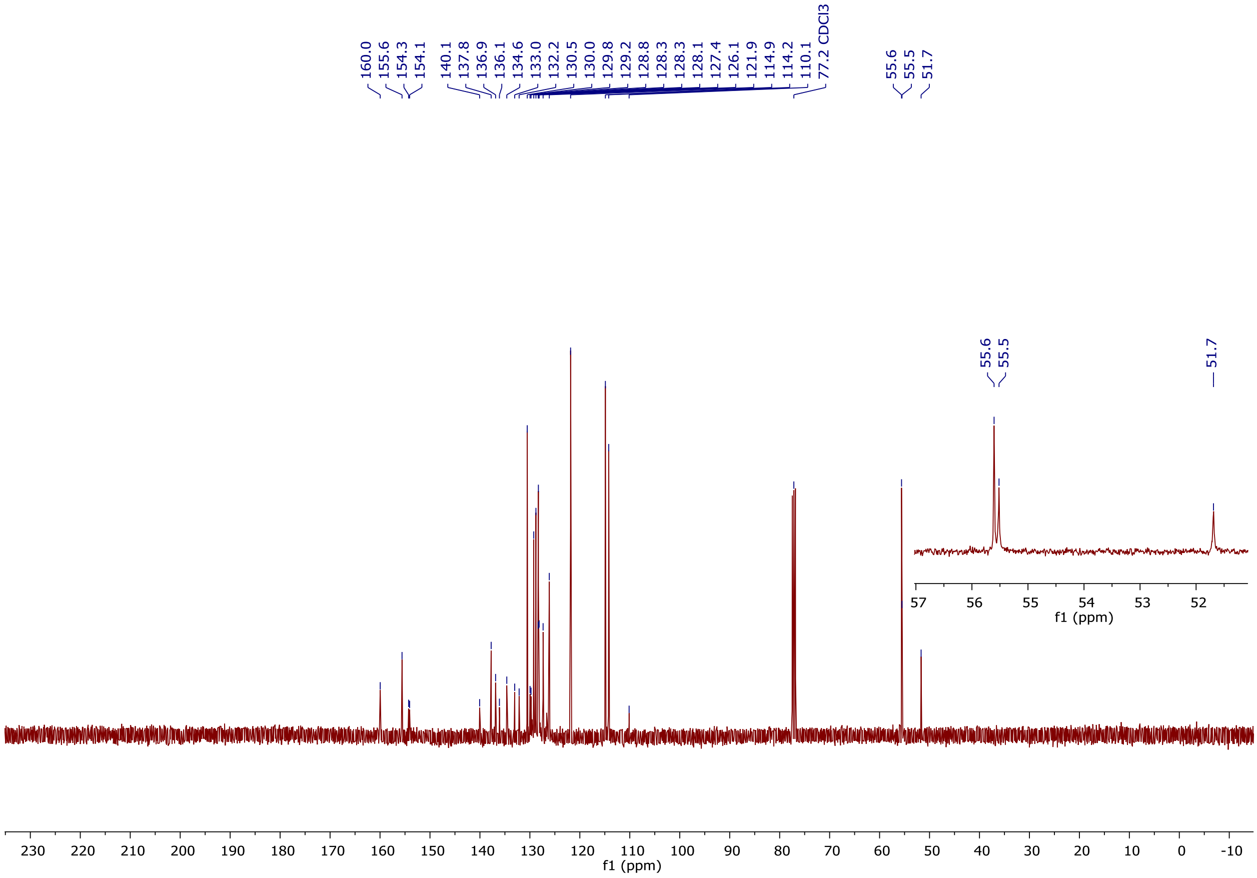

Figure S50. ^{13}C NMR (101 MHz, chloroform-*d*) spectrum of compound **6i**

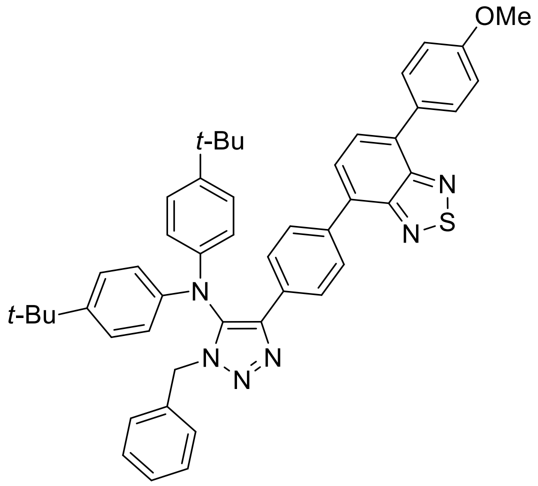

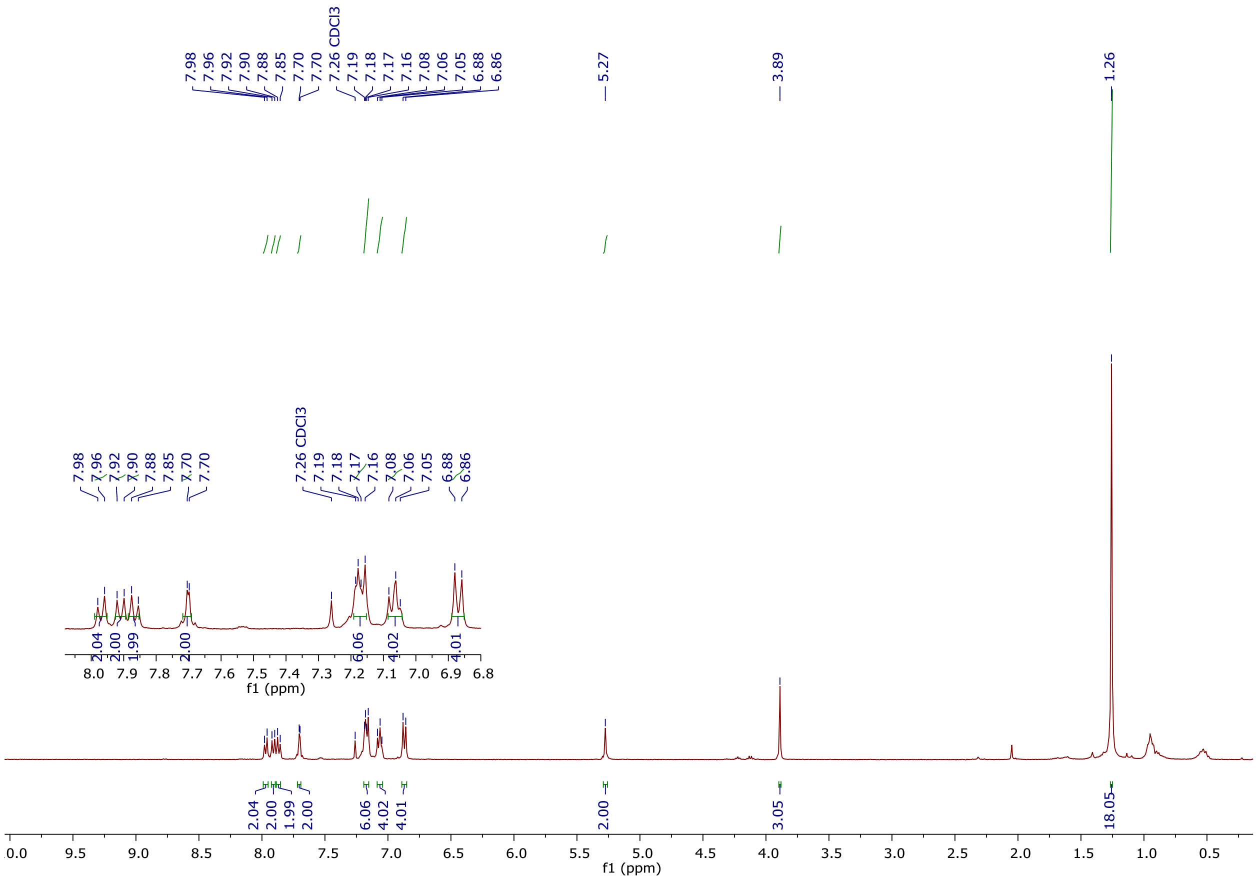

Figure S51. ^{1}H NMR (400 MHz, chloroform-*d*) spectrum of compound **6j**

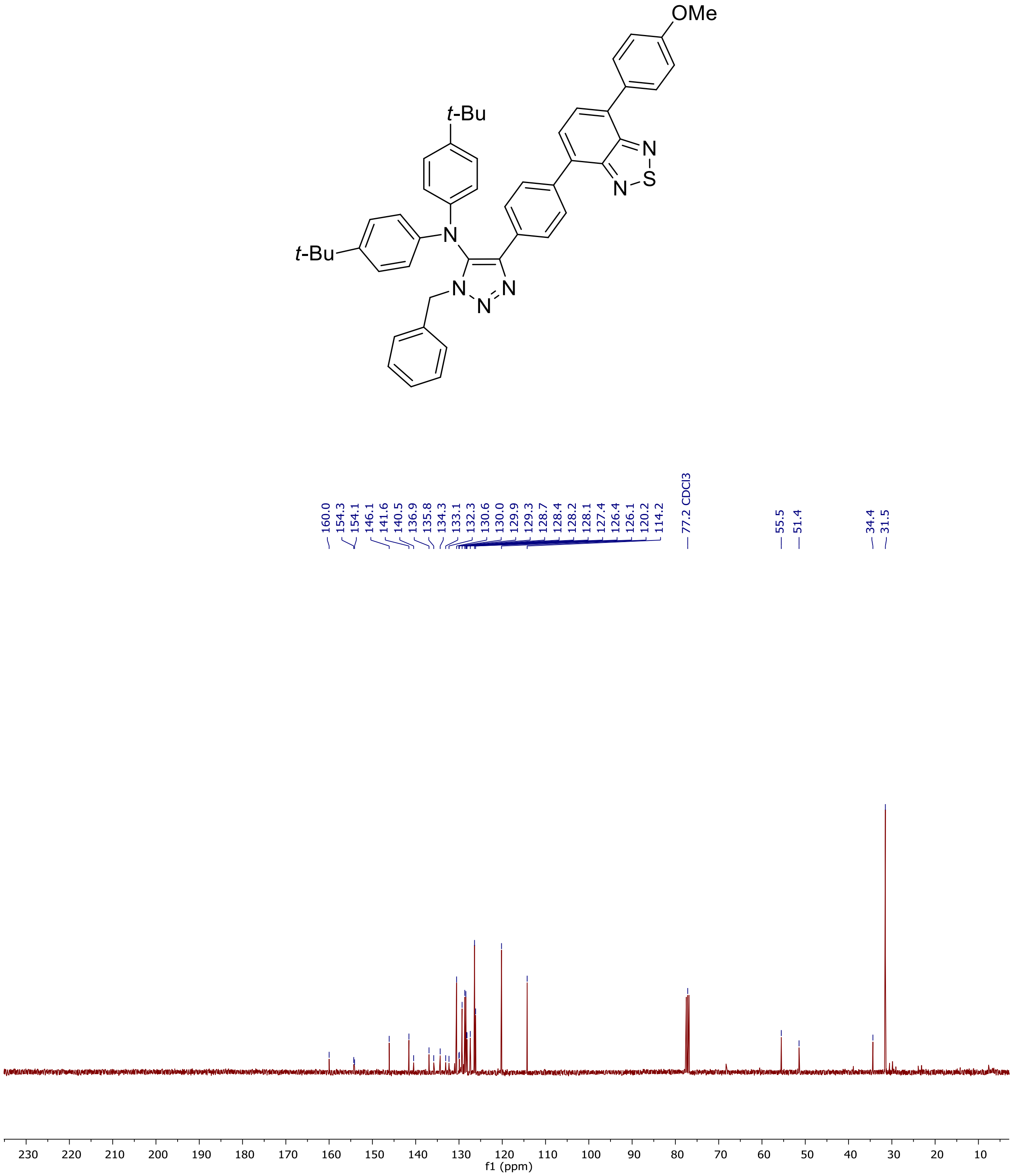

Figure S52. ^{13}C NMR (101 MHz, chloroform-*d*) spectrum of compound **6j**

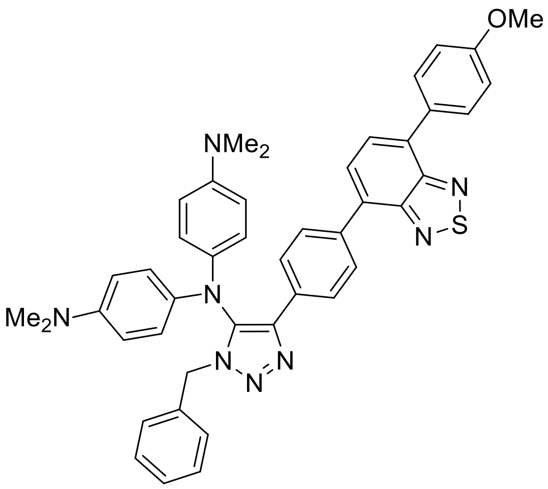

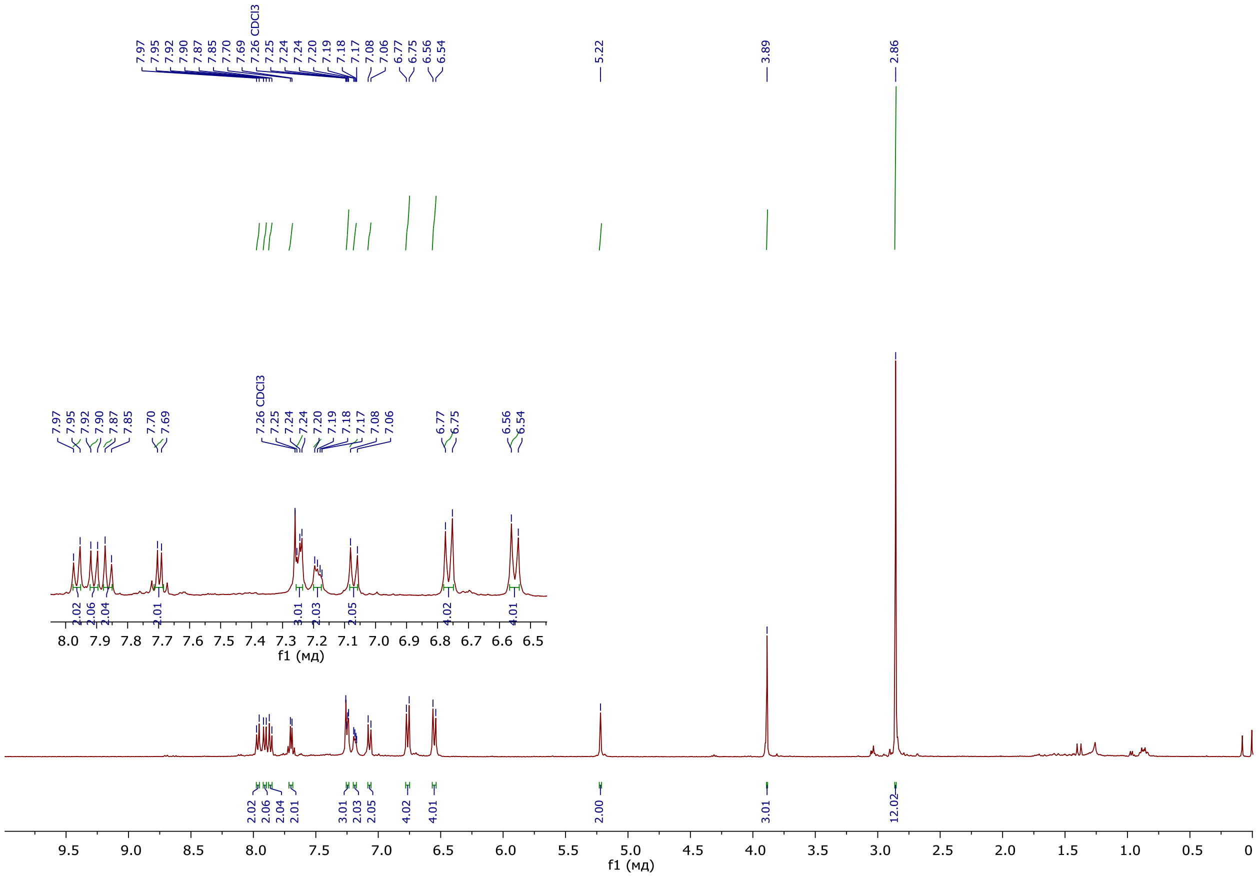

Figure S53. ^{1}H NMR (400 MHz, chloroform-*d*) spectrum of compound **6k**

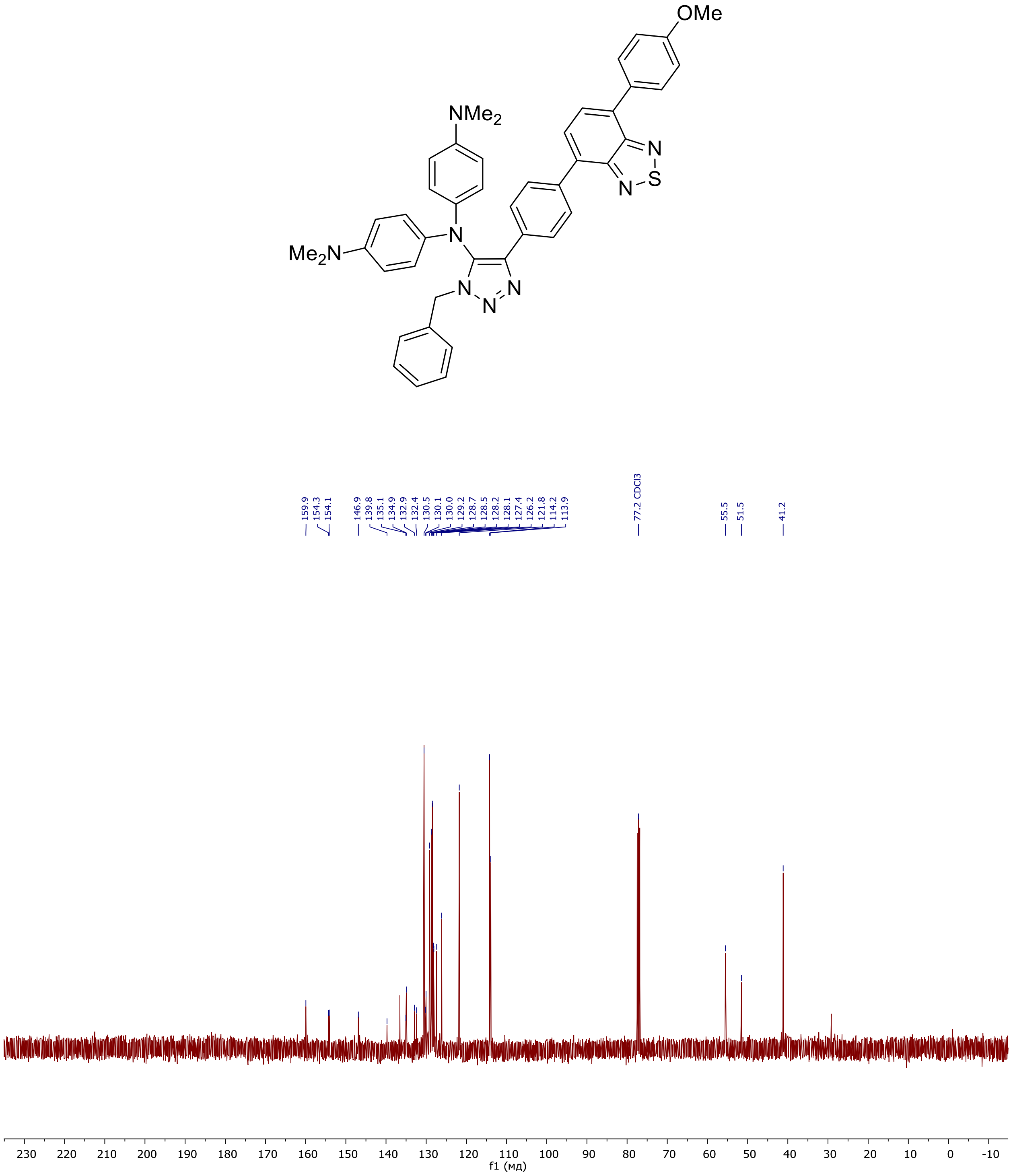

Figure S54. ^{13}C NMR (101 MHz, chloroform-*d*) spectrum of compound **6k**

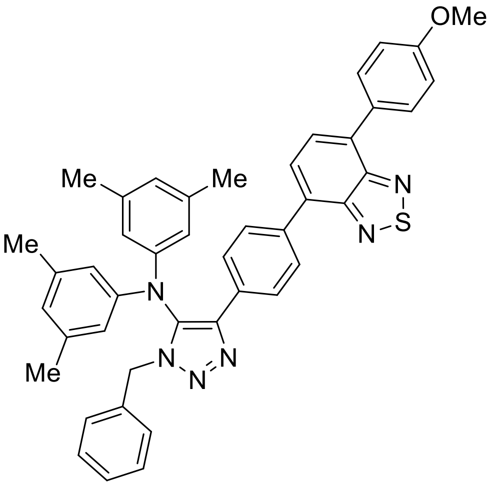

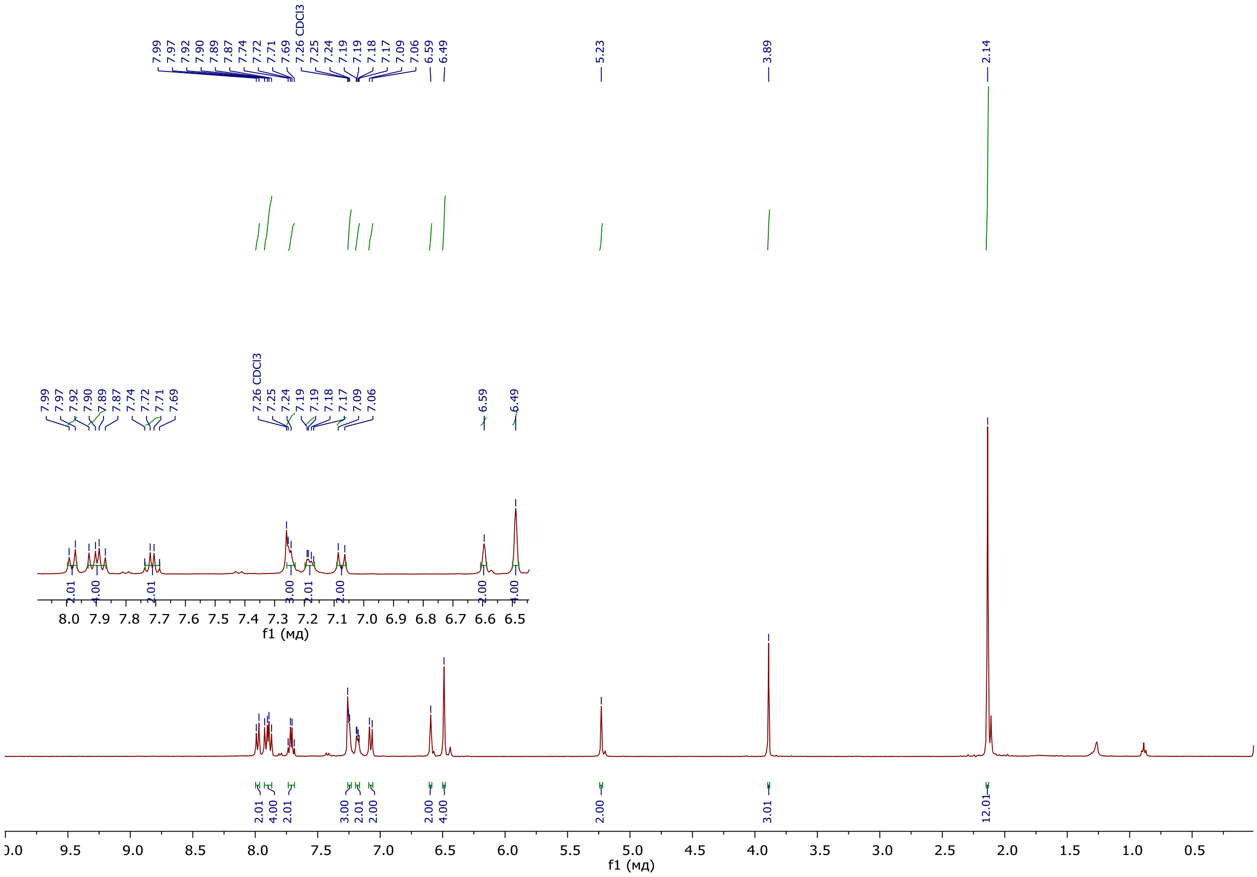

Figure S55. ^{1}H NMR (400 MHz, chloroform-*d*) spectrum of compound **6l**

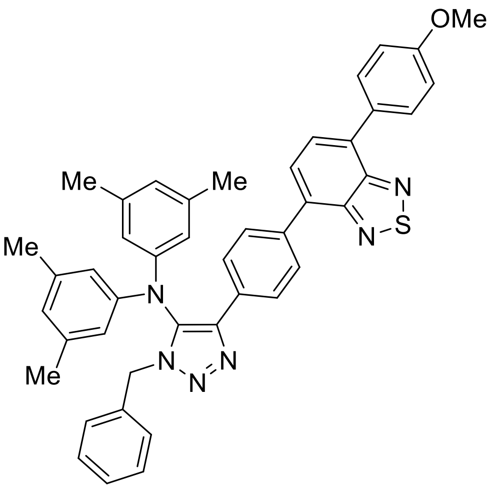

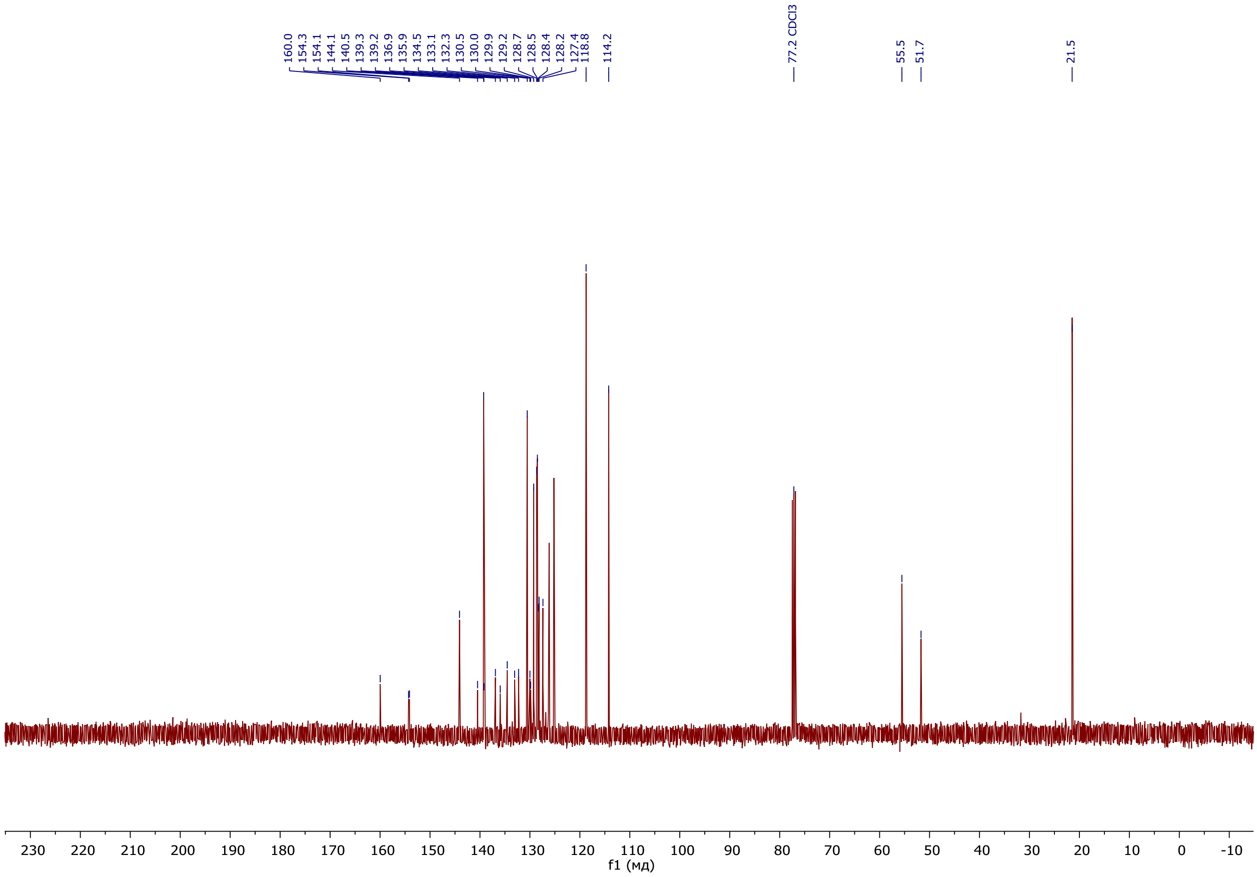

Figure S56. ^{13}C NMR (101 MHz, chloroform-*d*) spectrum of compound **6l**

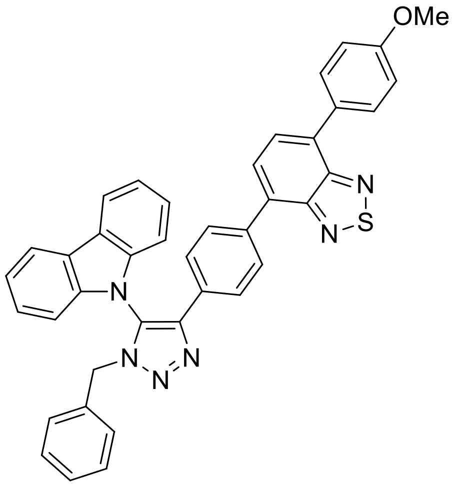

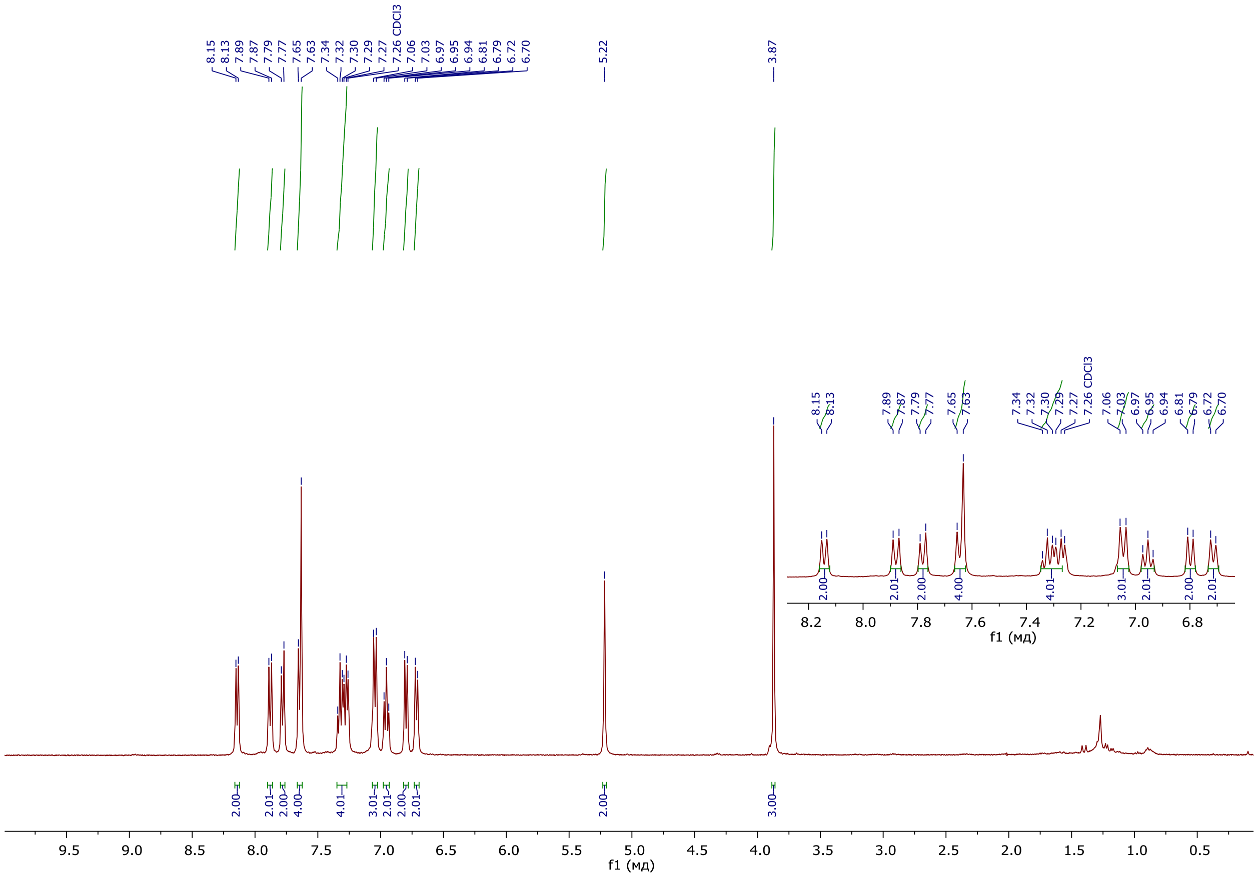

Figure S57. ^{1}H NMR (400 MHz, chloroform-*d*) spectrum of compound **7a**

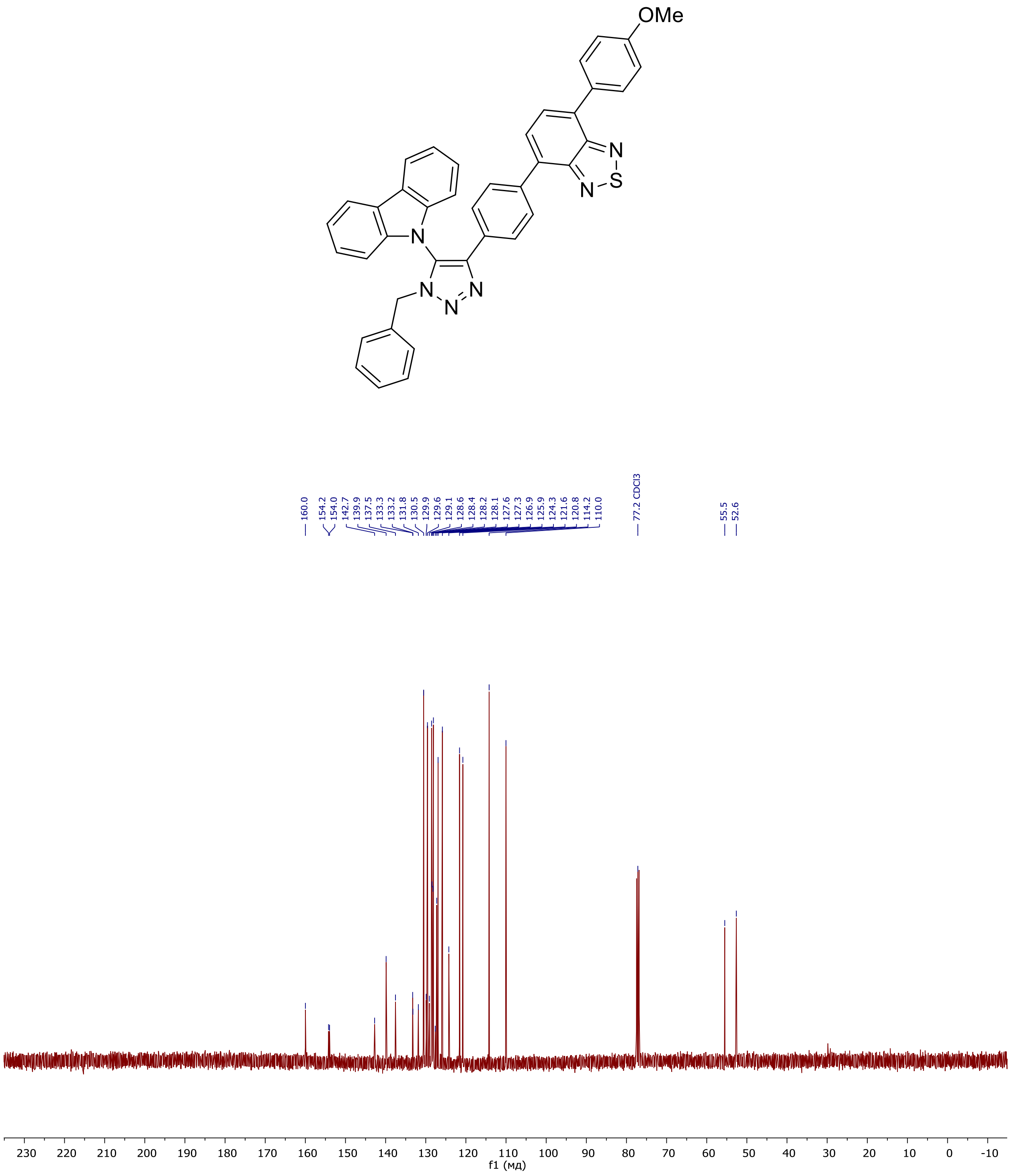

Figure S58. ^{13}C NMR (101 MHz, chloroform-*d*) spectrum of compound **7a**

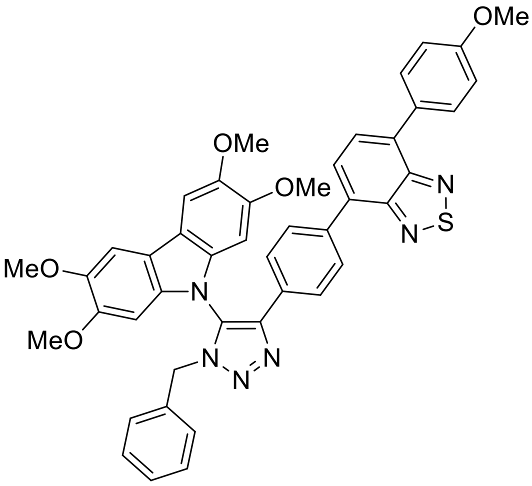

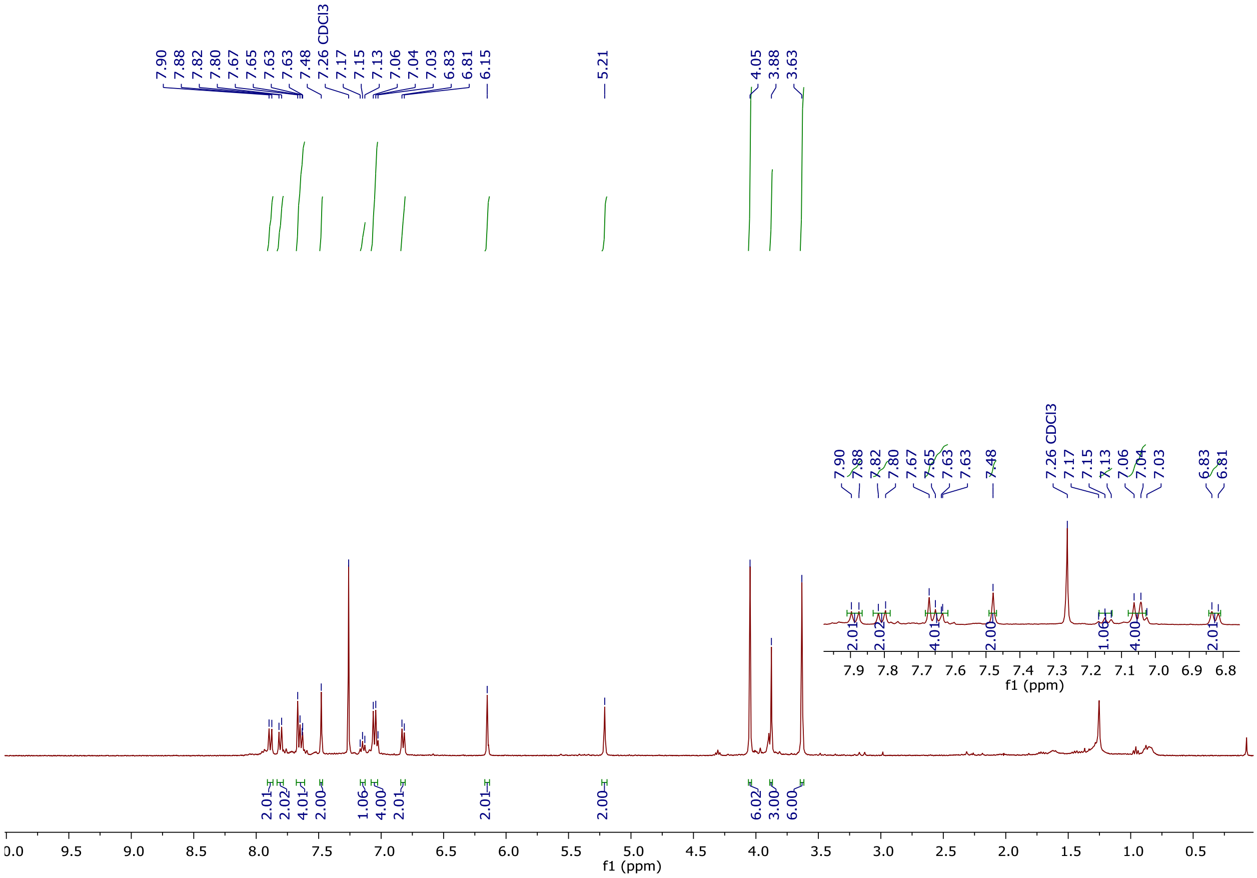

Figure S59. ^{1}H NMR (400 MHz, chloroform-*d*) spectrum of compound **7b**

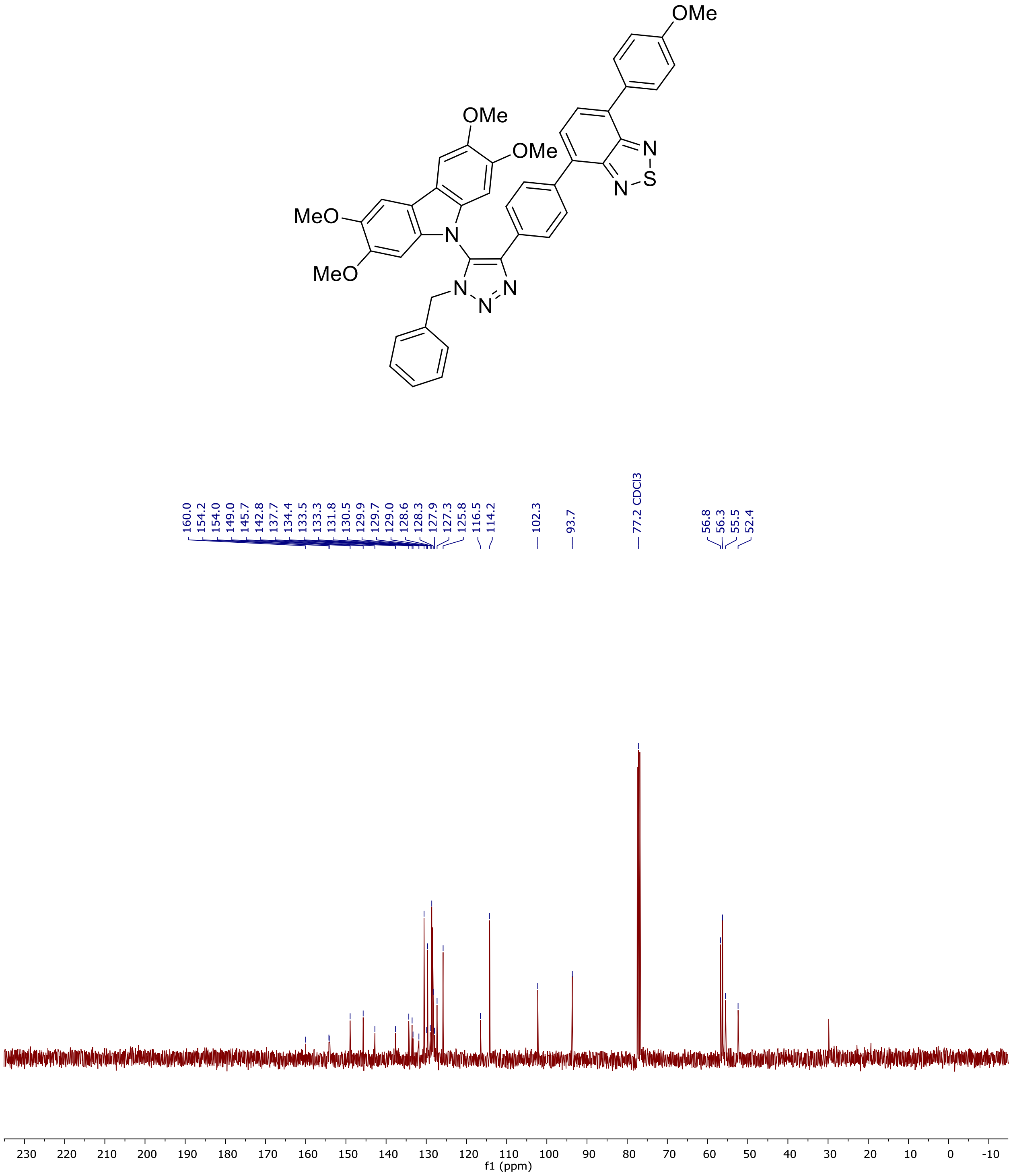

Figure S60. ^{13}C NMR (101 MHz, chloroform-*d*) spectrum of compound **7b**

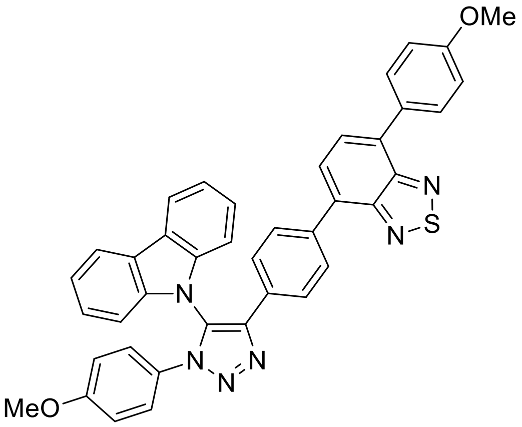

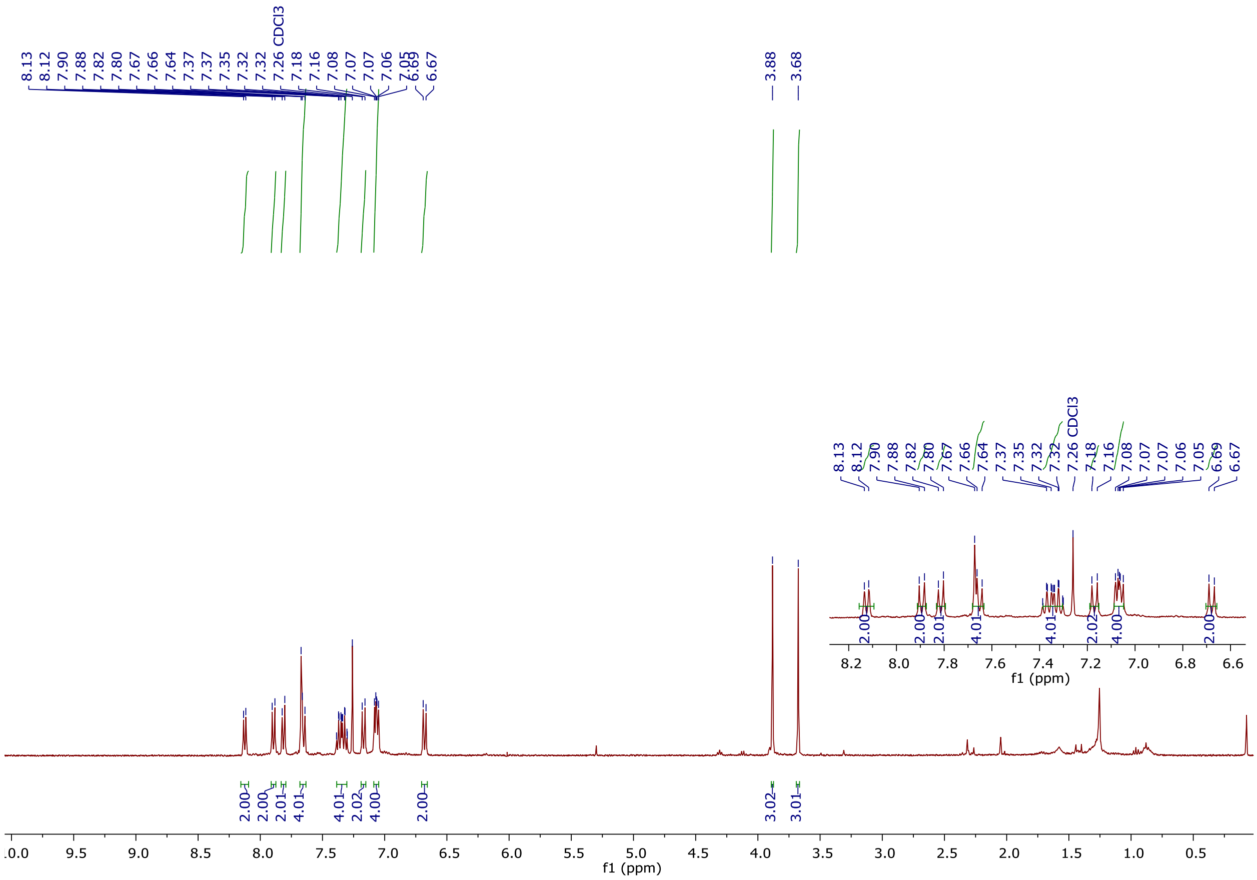

Figure S61. ^{1}H NMR (400 MHz, chloroform-*d*) spectrum of compound **7c**

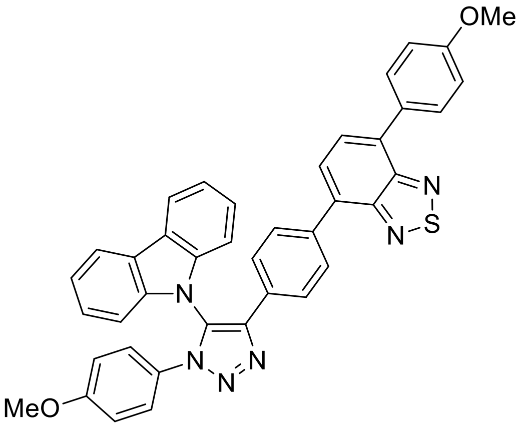

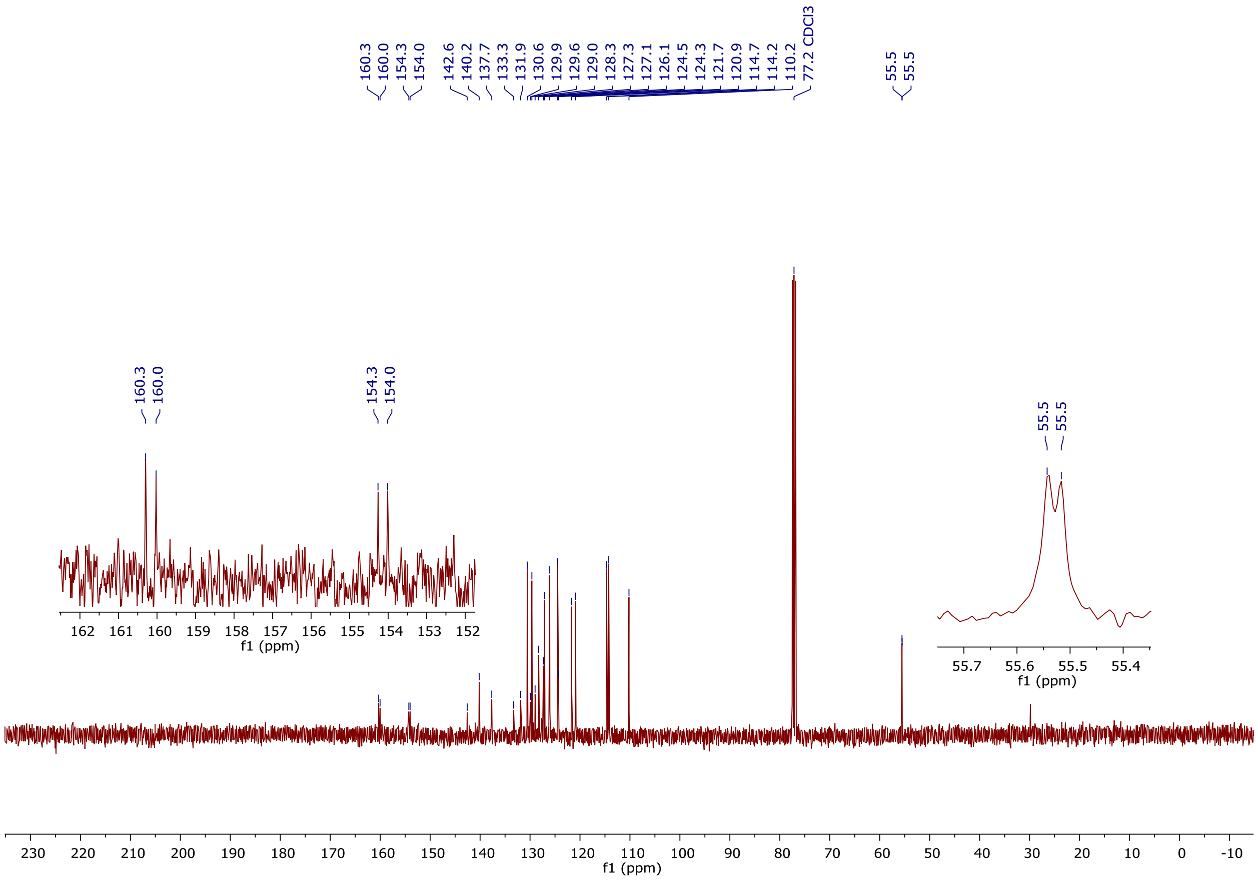

Figure S62. ^{13}C NMR (101 MHz, chloroform-*d*) spectrum of compound **7c**

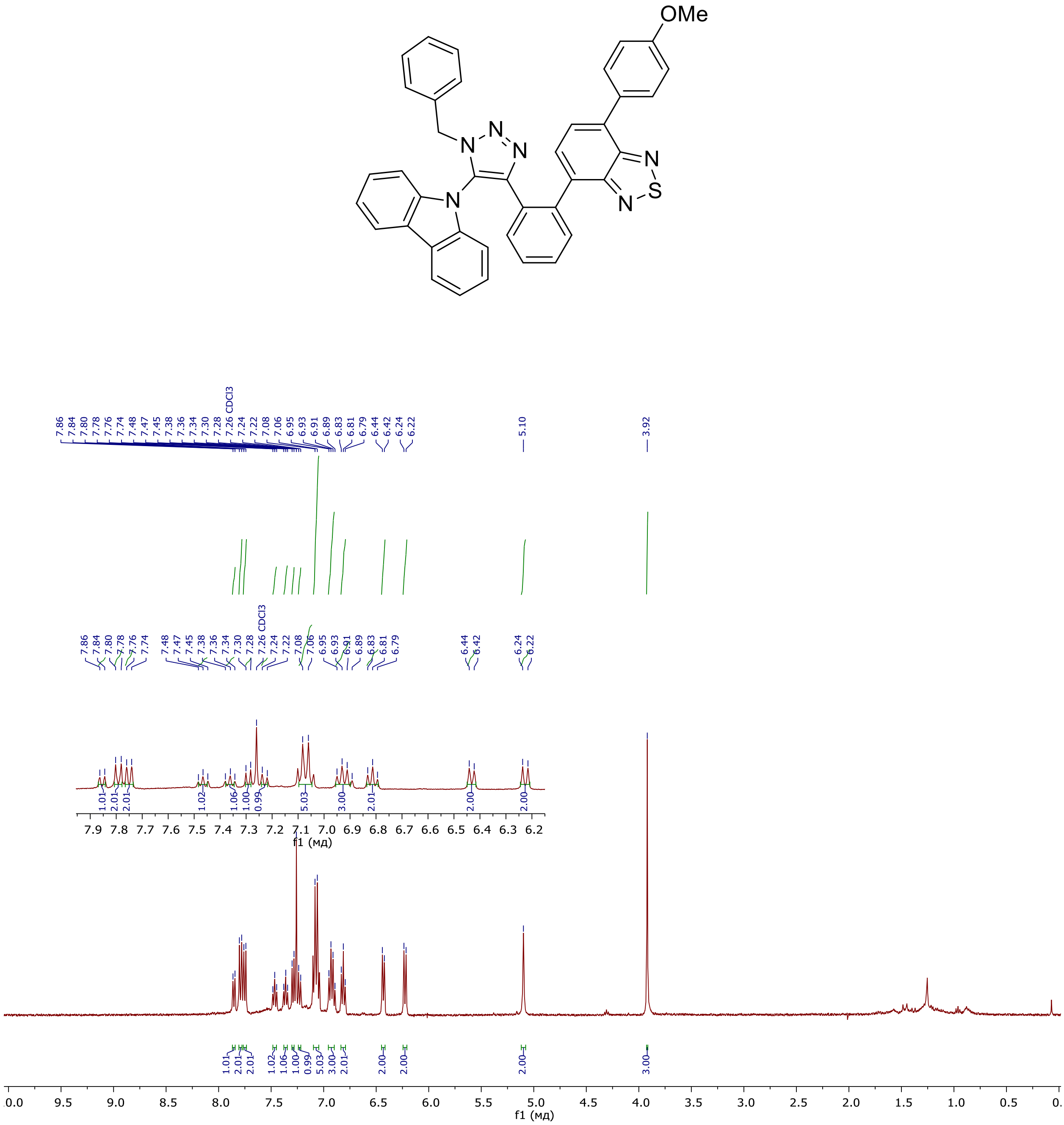

Figure S63. ^{1}H NMR (400 MHz, chloroform-*d*) spectrum of compound **7d**

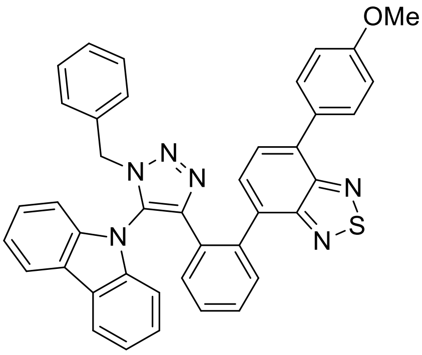

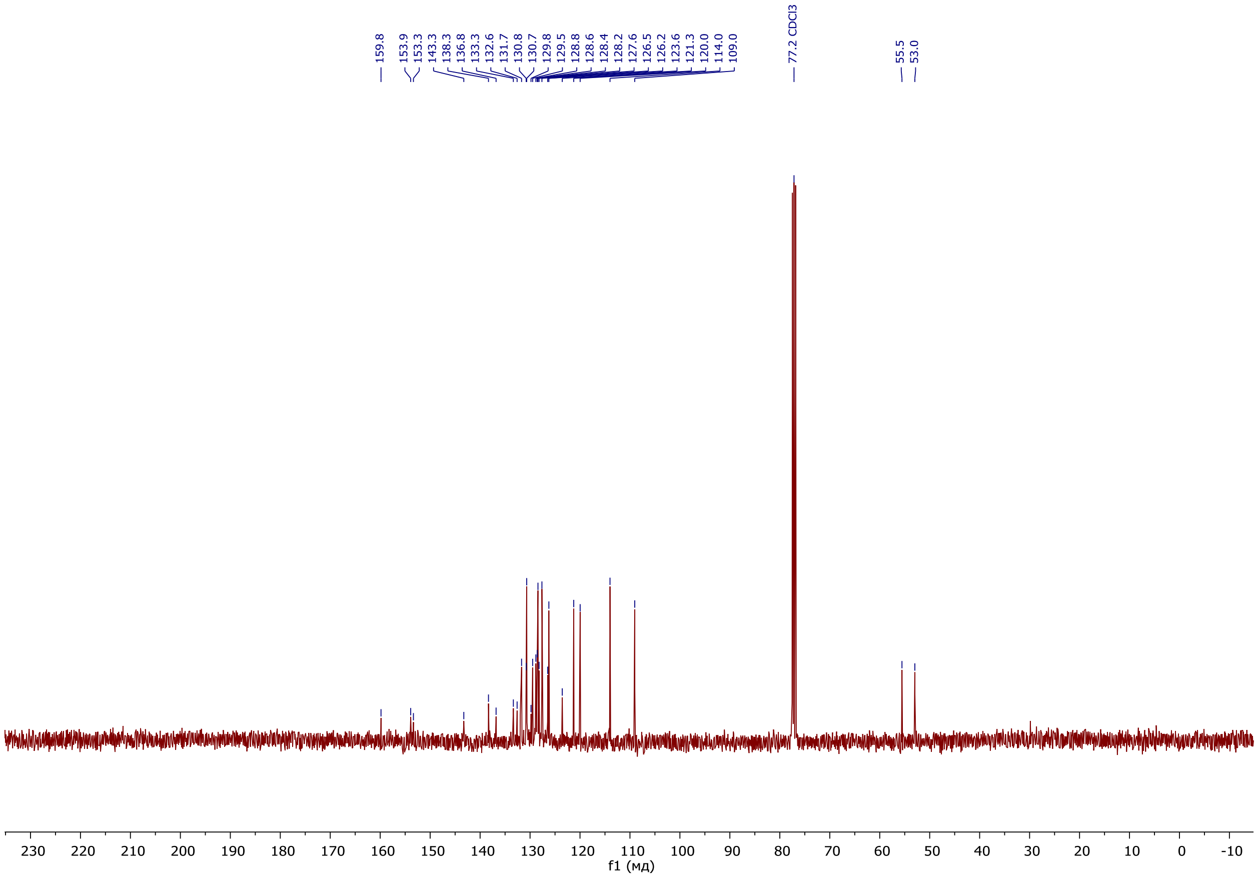

Figure S64. ^{13}C NMR (101 MHz, chloroform-*d*) spectrum of compound **7d**

Electrochemistry

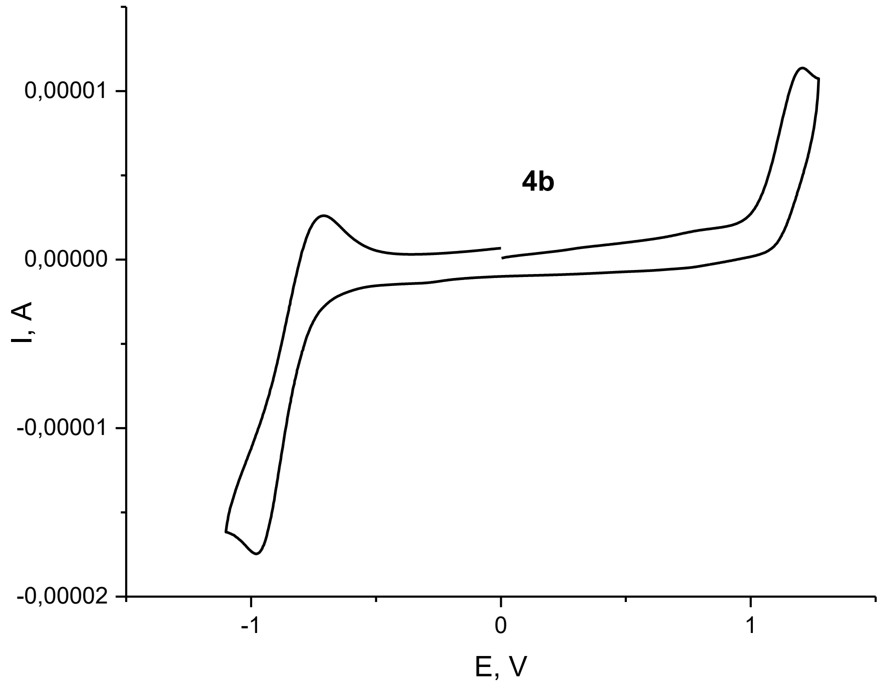

Figure S65. Cyclic voltammogram of **4b**

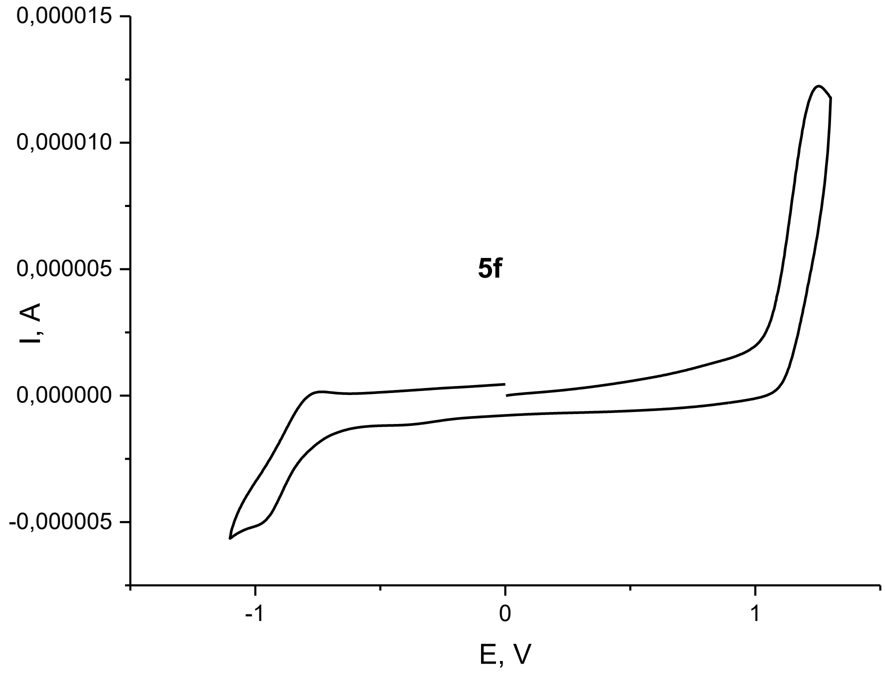

Figure S66. Cyclic voltammogram of **5f**

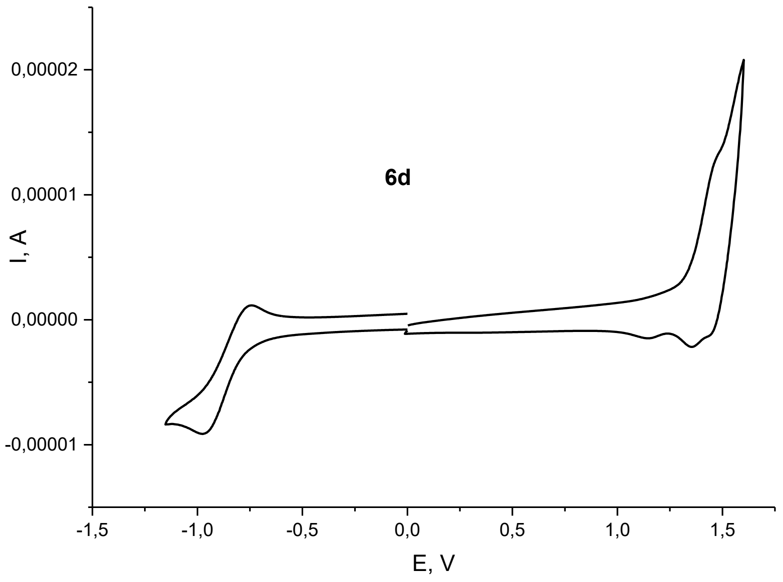

Figure S67. Cyclic voltammogram of **6d**

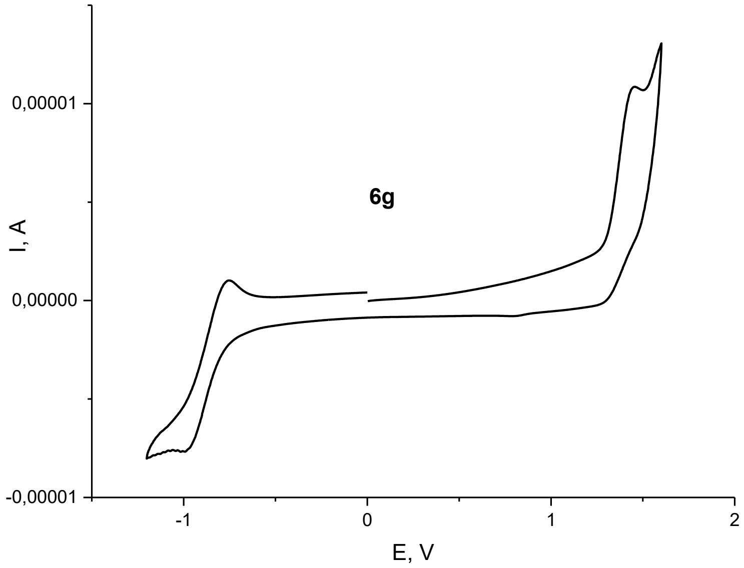

Figure S68. Cyclic voltammogram of **6g**

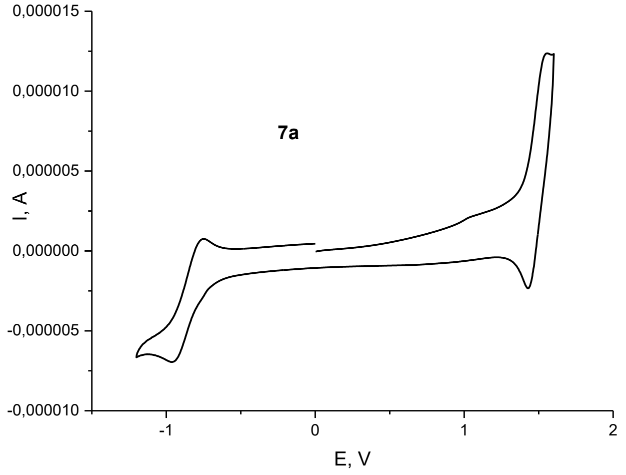

Figure S69. Cyclic voltammogram of **7a**
